# Can incontinence be cured? A systematic review of cure rates

**DOI:** 10.1186/s12916-017-0828-2

**Published:** 2017-03-24

**Authors:** Rob Riemsma, Suzanne Hagen, Ruth Kirschner-Hermanns, Christine Norton, Helle Wijk, Karl-Erik Andersson, Christopher Chapple, Julian Spinks, Adrian Wagg, Edward Hutt, Kate Misso, Sohan Deshpande, Jos Kleijnen, Ian Milsom

**Affiliations:** 1 Kleijnen Systematic Reviews Ltd, Unit 6, Escrick Business Park, Riccall Road, Escrick, York, YO19 6FD UK; 2 NMAHP Research Unit, Glasgow Caledonian University, Glasgow, UK; 3 Neuro-Urologie, Clinic of Urology, University Clinic Friedrich-Wilhelms-University Bonn and Neurologic Rehabilitation Center Godeshoehe, Bonn, Germany; 4 Florence Nightingale Faculty of Nursing and Midwifery King’s College London, London, UK; 5 Centre for Person-Centred Care (GPCC), Institution for Health and Care Science, Sahlgrenska Academy, University of Gothenburg, Gothenburg, Sweden; 6 Clinical Medicine, Aarhus University, Aarhus, Denmark; 7 Department of Urology, Sheffield Teaching Hospitals NHS Foundation Trust, Royal Hallamshire Hospital, Sheffield, UK; 8 Court View Surgery, Strood, UK; 9 Division of Geriatric Medicine, University of Alberta, Edmonton, Canada; 10 Medica Market Access Ltd, Tonbridge, UK; 11 Department of Family Medicine, School for Public Health and Primary Care (CAPHRI), Maastricht University, Maastricht, The Netherlands; 12 Department of Obstetrics & Gynecology, Institute of Clinical Sciences, Sahlgrenska Academy at Gothenburg University, Gothenburg, Sweden

**Keywords:** Incontinence, Systematic review, Cure rates

## Abstract

**Background:**

Incontinence constitutes a major health problem affecting millions of people worldwide. The present study aims to assess cure rates from treating urinary (UI) or fecal incontinence (FI) and the number of people who may remain dependent on containment strategies.

**Methods:**

Medline, Embase, PsycINFO, Cochrane Central Register of Controlled Trials (CENTRAL), CINAHL, and PEDro were searched from January 2005 to June 2015. Supplementary searches included conference abstracts and trials registers (2013–2015). Included studies had patients ≥ 18 years with UI or FI, reported treatment cure or success rates, had ≥ 50 patients treated with any intervention recognized in international guideline algorithms, a follow-up ≥ 3 months, and were published from 2005 onwards. Title and abstract screening, full paper screening, data extraction and risk-of-bias assessment were performed independently by two reviewers. Disagreements were resolved through discussion or referral to a third reviewer where necessary. A narrative summary of included studies is presented.

**Results:**

Most evidence was found for UI: Surgical interventions for stress UI showed a median cure rate of 82.3% (interquartile range (IQR), 72–89.5%); people with urgency UI were mostly treated using medications (median cure rate for antimuscarinics = 49%; IQR, 35.6–58%). Pelvic floor muscle training and bulking agents showed lower cure rates for UI. Sacral neuromodulation for FI had a median cure rate of 38.6% (IQR, 35.6–40.6%).

**Conclusions:**

Many individuals were not cured and hence may continue to rely on containment. No studies were found assessing success of containment strategies. There was a lack of data in the disabled and in those with neurological diseases, in the elderly and those with cognitive impairment. Surgical interventions were effective for stress UI. Other interventions for UI and FI showed lower cure rates. Many individuals are likely to be reliant on containment strategies.

**PROSPERO Registration:**

PROSPERO registration number: CRD42015023763.

**Electronic supplementary material:**

The online version of this article (doi:10.1186/s12916-017-0828-2) contains supplementary material, which is available to authorized users.

## Background

Urinary incontinence (UI) constitutes a major health problem affecting the lives of an estimated 400 million persons worldwide [1–6]. The prevalence of UI increases with age and is highly prevalent in the elderly and those with cognitive impairment [5]. Fecal incontinence (FI) occurs in up to 6% of those younger than 40 years increasing to 15% in older individuals [1, 2, 4]. Combined FI and UI has been reported in 10% of women and in 6–10% of men living in the community, increasing to almost 50% in nursing home residents [7–9]. Incontinence is also more prevalent in men and women affected by various neurological illnesses, e.g., multiple sclerosis, spina bifida, Parkinson’s disease, and stroke [5]. The World Health Organization has acknowledged incontinence as a set of diseases (International Classification of Diseases ICD-10) and the International Classification of Functionality recognizes the associated extreme disablement [5]. Global demographic trends suggest that the incidence of both UI and FI will rise in the coming years, with a significant health and social burden as well as an increased economic cost for both patients and health service payers [5].

Incontinence has a profound impact on well-being and quality of life, causing social embarrassment, reduced employment and work productivity, and preventing many people from participating in paid or unpaid activities [5, 10, 11]. Further, it has a negative influence on sexual health [11–13]. There is also a significant impact on caregivers and, for older people, incontinence significantly increases the likelihood of institutionalization [5]. Incontinence presents a significant health and economic burden comparable with common major diseases such as arthritis and pneumonia [14–17].

Various management options are available for UI, many of which are given in combination, including behavioral techniques, pelvic floor muscle training (PFMT), medication, surgery, nerve stimulation, and containment products such as pads and catheters [18]. Treatment for FI includes diet adaptation, medication, biofeedback, PFMT, surgery, sacral or tibial nerve stimulation, and containment products [19].

It is important to guide health service payers and providers as to how continence care might best be configured to deliver efficient, guideline-compliant, high-quality patient care [12]. It has previously been suggested that the provision of continence management be considered for four patient profiles, namely those with (1) UI, (2) FI, (3) disabled/neurological illnesses, or (4) the elderly/cognitively impaired [20].

Given the impact of incontinence, it is important to know how large the residual problem is following treatment. Although there are numerous systematic reviews of the relative effects of various treatments, there is no review which addresses cure rates following treatment. The aim of this systematic review was to establish cure rates based on extent of leakage after treating incontinence for each of the four pre-defined patient profiles and to assess the number of people still dependent on containment (including behavioral strategies and containment products) following treatment in order to live a normal life.

## Methods

### Objectives

The objectives of this review were to describe cure rates of treatment in patients with UI or FI and estimate how many people remained dependent on containment strategies. A further objective was to determine the extent to which containment strategies facilitated individuals leading a normal life. Pre-specified subgroups were:

- UI: Defined as involuntary loss of urine according to International Continence Society/International Urogynecological Association terminology [21]. This profile included people experiencing stress (SUI), urgency (UUI), and mixed (MUI) UI not covered by the other categories.
- FI: Defined as loss of control of liquid or solid stool.
- Disabled persons and those with neurological problems or diseases: Conditions including Parkinson’s disease, multiple sclerosis, multiple system atrophy, stroke, and spina bifida.
- Elderly or cognitively impaired: Defined as those ≥ 65 years and those with, among other conditions, Alzheimer’s disease.

Cure was defined as no leakage (UI) and/or no episodes of FI at trial specified time points, of at least 3 months. Success rates for containment were defined as the percentage of patients with no limitations to activities of daily living, quality of life, or social interaction.

### Data sources and searches

#### Inclusion criteria

Studies of any design which included adult patients (≥18 years) with UI or FI, reporting cure or success rates, with ≥ 50 patients, evaluating any intervention in line with the 5th International Consultation on Incontinence (ICI) treatment algorithms [22] (which includes primary, secondary and additional lines of therapy), a follow-up time ≥ 3 months, and published between January 2005 and June 2015 were included. Medline, Embase, PsycINFO, the Cochrane Central Register of Controlled Trials (CENTRAL), CINAHL, and PEDro were searched. Supplementary searches were undertaken for conference abstracts and trials registers published in 2014 and 2015 (Additional file 1).

### Study selection

Titles and abstracts of retrieved references were screened for relevance independently by two reviewers. Disagreements were resolved through discussion or referral to a third reviewer where necessary. References requiring further scrutiny were ordered and full papers were screened for relevance independently by two reviewers. Disagreements were resolved as for titles/abstracts.

### Data extraction and quality assessment

Data extraction was performed independently by two reviewers using an a priori designed data extraction form. Disagreements were resolved through discussion or referral to a third reviewer where necessary. The Downs and Black checklist was used for quality assessment [23]. The checklist has 28 questions that can each be answered with ‘Yes’ or ‘No’, indicating high or low risk of bias; eight questions were not applicable, for example, as we were only interested in the number of patients with a certain outcome, variability is not an issue (Questions 7 and 10). Questions that were of specific relevance to the current review were Q2 – outcome description, Q3 – description of patients included, Q4 –description of the intervention, and Q12 – representativeness of patients included. Quality assessment was independently conducted by two reviewers. Disagreements were resolved through discussion or referral to a third reviewer where necessary.

### Data synthesis and analysis

A narrative summary of all included studies, including a summary of the characteristics (e.g. study design, sample size, geographical location, year, baseline population characteristics, outcome definition and assessments) and methodological quality of the studies is presented. As this review focuses on cure rates of different interventions and subsequent dependence on containment products, and not the relative effectiveness of interventions compared with each other, we included cure rates from individual study arms. Cure rates are expressed in percentages and are calculated as follows: number of patients who had no leakage and/or no episodes of FI divided by the total number of patients in the study population at a given time point. Where possible, results in the text are pooled by expressing the results as medians with interquartile ranges (IQR) including results at all follow-up points (as reported in the summary tables for each population), unless described otherwise. If there were insufficient data to report medians, results for individual studies are presented. In studies including different populations, for instance, people with SUI and UUI, cure rates for the specific population (UUI or SUI) have been reported where possible; if this was not possible, the cure rate for the whole population was reported in the SUI and UUI sections.

## Results

**Fig. 1:**
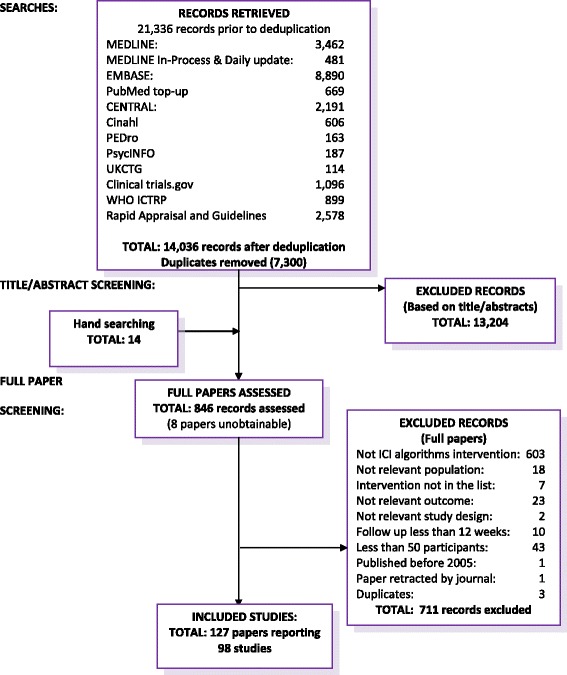
Summary of study flow

The searches retrieved 14,036 records. After title and abstract screening, 846 references remained; these were ordered for full review and 8 were unobtainable. The reasons for exclusion are listed in the flow chart (Fig. 1). The most common reason for exclusion was that the treatment was not deemed ‘according to the ICI algorithms’ (603 records excluded); often because the intervention was a second or third line treatment and there was no description of previous interventions in first or second line.

In total, 127 papers reporting on 98 individual studies were included. Initially, 30 papers (24 studies) with a follow-up of 12 weeks were excluded in accordance with the protocol. However, after discussions with clinicians, it was concluded that readers with a clinical perspective would most likely consider 12 weeks and 3 months to be equivalent, and therefore these 30 papers were included.

Forty-six studies were performed in Europe, 20 were from North America and 15 were from centers on more than one continent (Europe and America or Australia and America). Forty-four studies included only female patients and another 32 studies included more than 60% female patients. The 98 studies included 150 interventions (Additional file 2: Table S1). Most interventions were surgically inserted tapes and slings (n = 45) or pharmacological treatments (n = 45), 18 were PFMT interventions. None of the included studies examined success rates for containment strategies.

Most studies had at least 10 out of 20 items with low risk of bias (Additional file 2: Table S2). Five studies had more than 17 items with low risk of bias, and nine studies had fewer than 10 items with low risk of bias. All studies clearly described aims and objectives and, hence, were at low risk of bias for question 1. Items with the most studies scoring high risk of bias were Q15 – blinding of outcome assessors, Q23 – random patient selection, and Q26 – loss to follow-up explained. For all other items more than 80% of studies were at low risk of bias.

Results are reported by patient profile (UI, FI, Disabled and neurological problems or diseases, and Elderly or cognitively impaired).

### Studies in patients with UI

Interventions for SUI reported a median cure rate of 32% (IQR, 28–36%) for open colposuspension and 82.3% (IQR, 72–89.5%) for other surgical techniques. UUI, mostly treated by pharmacological means, had a median cure rate of 45.8% (IQR, 35.6–57.8%) for drug treatments, depending on the drug used. MUI demonstrated intermediate cure results, falling between those for SUI and UUI, depending on the nature of the underlying condition and the intervention employed.

#### SUI

Forty-four studies looked at 67 interventions in patients with SUI. Thirty-four studies were predominantly in women, eight studies were predominantly in men; in two studies, gender was not reported (Additional file 2: Tables S3 and S4). Thirty-five studies were specifically in patients with SUI, seven studies included patients with MUI and SUI, one study included patients with UUI and SUI, and one study included patients with UUI, MUI and SUI.

Results are reported in Additional file 2: Table S5 and an overview of the summary of cure rates is reported in Table 1. All cure rates for all follow-up points within each study reported in the included studies are listed in Additional file 2: Table S5. The summary table (Table 1) lists all cure rates at the final follow-up for each study. In the text below, pooled results for each intervention are reported as specified in the Methods section.

**Table 1:** Cure rates^a^ in studies for female and male patients with stress urinary incontinence

Follow-up	3 m	6 m	12 m	18 m	2 y	3 y	5 y
Treatment – WOMEN							
TVT	78%	50%	53%; 90.1% W			89.5%	81% W
		92%					
TVT-O			82.3%; 94.1%; 92.3%; 76%; 88.4% W; 88.8%		92.6%	86.4%; 87% 72%	74%
TVT Surgery/Individually tailored/MiniTape/SPARC	83.1% W (Primus)	27.7% W (North)	85%; 82% (VL)		31.6% W (North)		
		82.3% W (Primus)	32.3% W (North)				
			84.4% W (Primus)				
TOT	82.8%	84.3%	63.4%; 74%; 88.6%; 93.3%			65%	
Retropubic TVT		89.7%	86.1%; 65.5%; 81%	80.1%	77.4%		
Sling	94.1%	92.2% W; 81.3%	81.2%; 90.8%; 90.1% W; 63.7%; 90.2%; 92.2%; 41%	52.8%	83.7%; 50.5%		
		89.8%; 81%; 44%					
Colposuspension		22%/32%					90% W
		28%/36%					
Other (surgery)			79%				
PFMT – supervised	52.9%; 5%		58.8%				
Vaginal cone therapy	9%						
Lifestyle advice – unsupervised	8%						
Injectable bulking agents			36.9%; 24.8%				
Duloxetine	NR						
Treatment – MEN							
Male sling	80%	55.8% W	58% W; 51.4% W; 53.8%	42.5%	48% W; 54% W	40% W; 53%	
PFMT – supervised	51.9%	78%					
PFMT – unsupervised			NR				
PFMT – supervised + drug	78%	62%					

^a^Cure defined as ‘cure’, ‘objective cure’, or ‘success (dry)’

*BI* behavioral interventions; *NR* not reported; *PFMT* pelvic floor muscle training; *TOT* transobturator tape; *TVT* tension-free vaginal tape; *TVT–O* tension-free vaginal tape–obturator; *W* with containment products; *VL* Van Leijsen 2013*; North *North 2010; *Primus *Primus 2006

The majority of the studies evaluating different interventions for patients with SUI reported follow-up at 3, 6 and 12 months. At 12 months, the median cure rate for surgical interventions for women was 84.4% (IQR, 74–90.1%). For men, results for male slings were reported for up to 3 years’ follow-up, with a median cure rate over all follow-up periods of 53% (IQR, 48–54%). One study examined supervised PFMT for women and found a cure rate of 58.8% at 12 months. For men, the cure rates for PFMT at 3 and 6 months were 51.9% and 78%, respectively. Treatment with injectable bulking agents showed cure rates of 24.8% and 36.9% at 12 months’ follow-up.

#### UUI

Thirty-two studies looked at 54 interventions for patients with UUI (Additional file 2: Tables S6 and S7). Eight of the 32 studies were in women only, 22 of the remaining 24 studies included more than 60% women. The remaining two studies only included men. Two studies included patients with MUI and UUI [24, 25], one study included UUI and SUI [26], and one study included SUI, MUI and UUI [27].

Results are reported in Additional file 2: Table S8 and an overview of the summary of cure rates is reported in Table 2. All cure rates for all follow-up points within each study reported in the included studies are listed in Additional file 2: Table S8. The summary table (Table 2) lists all cure rates at the final follow-up for each study. In the text below, pooled results for each intervention are reported as specified in the Methods section.

**Table 2:** Cure rates^a^ in studies for female and male patients with urgency urinary incontinence

Follow-up:	3 m	6 m	12 m	18 m	2 y	3 y	5 y
Treatment – WOMEN							
Antimuscarinic		21%					
Darifenacin^b^	38%	41%	42%		43.8%		
Fesoterodine	63%; 62%; 64%; 49.2%; 57.8%						
Oxybutynin	25.2%; 20%						
Solifenacin	58%; 59%; 56.2%; 59.6%	11%	58%				
Tolterodine	13%; 56% 57.2%; 49%	70%	45.1%				
Tolterodine + BI		NR					
Trospium	35.6%; 20.5%						
Adrenergic drugs (Mirabegron)	47.1%		43.4%; 45.8%				
Neuromodulation		39%					15% (10 y: 17%)
Neuromodulation + PFMR	93%	39%					15% (10 y: 17%)
TVT		92%					
Botulinum toxin	35%; 22.9% 15.9–50.9%	31.3%					
Treatment – MEN							
Solifenacin + Tamsulosin	NR						
PFMT – supervised			24%; 35%				
Lifestyle advice – unsupervised			23%; 38%				

^a^Cure defined as ‘cure’, ‘objective cure’, ‘success (dry)’, or complete continence

^b^Reductions of ≥ 90% from feeder-study baseline in incontinence episodes/week

*BI* behavioral interventions; *NR* not reported; *PFMT* pelvic floor muscle training; *TVT* tension-free vaginal tape

Most studies for patients with urgency UI evaluated pharmacological interventions and had follow-up of less than 1 year (Table 2). The median cure rate for antimuscarinics was 49% (IQR, 35.6–58%). For mirabegron, cure rates of 47.1% were found at 3 months’ follow-up and of 43.4% and 45.8% at 12 months. Cure rates of 15% and 17% were seen at 5 and 10 years, respectively, for sacral neuromodulation for women with UUI. OnabotulinumtoxinA achieved cure rates ranging from 15.9% for 50 U to 50.9% for 300 U at 3 months in one study, and 31.1% for 200 IU at 6 months in another study. These studies did not include patients with neurogenic incontinence.

Supervised PFMT interventions were only evaluated in men with UUI showing cure rates of 24% and 35% at 12 months.

#### MUI

Sixteen studies examined 23 interventions for patients with MUI (Additional file 2: Tables S9 and S10), with 10 studies in women only, one including 38% men, one including 20% men, and four including only male patients. Seven studies included patients with SUI and MUI, two studies included patients with MUI and UUI, and one study included patients with all three types of UI; the remaining six studies included patients with MUI.

Results are reported in Additional file 2: Table S11 and an overview of the summary of cure rates is reported in Table 3. All cure rates for all follow-up points within each study reported in the included studies are listed in Additional file 2: Table S11. The summary table (Table 3) lists all cure rates at the final follow-up for each study. In the text below, pooled results for each intervention are reported as specified in the Methods section.

**Table 3:** Cure rates^a^ in studies for female and male patients with mixed urinary incontinence

Follow-up	3 m	6 m	12 m	18 m	2 y	3 y	5 y
Treatment – WOMEN							
TVT		NR					
Retropubic TVT		89.7%	86.1%	80.1	77.4%		
SPARC	83.1% W	82.3% W	84.4% W				
Sling	94.1%	81.3%; 89.8%	63.7%; 90.2%	52.8%	50.5%; 83.7%;		
PFMT – supervised	5%				NR		8 y: NR
PFMT + neuromodulation^b^	93%						
PFMT + lifestyle advice	25%	28%					
Vaginal cone therapy	9%						
PFMT – supervised + delivery			17%				
Lifestyle advice (supervised)	8%						
Solifenacin			52%				
Duloxetine	NR						
Treatment – MEN							
Solifenacin	26.5%						
PFMT – supervised	44%; 46.3%	47%; 66.7%	24%; 35%; 60%; 83.4%				
PFMT – unsupervised	40%	50%	64%				
Lifestyle advice – unsupervised			23%; 38%				

^a^Cure defined as ‘cure’, ‘objective cure’, ‘success (dry)’ or complete continence

^b^Success, defined as ‘An absence of incontinent episodes (dry) and an OAB-V8 score < 8, indicating no OAB’

*NR* not reported; *PFMT* pelvic floor muscle training; *TVT* tension-free vaginal tape; *W* with containment products

The median cure rate for surgical interventions for women with MUI was 82.3% (IQR, 77.4–89.7%). The median cure rate for supervised PFMT interventions in men was 47% (IQR, 35–66.7%); for women, cure rates of 25% at 3 months and 28% at 6 months were reported.

No studies meeting inclusion criteria were found for the following interventions for UI: scheduled voiding (bladder training), continence products, artificial urinary sphincter, α-blockers, 5-alpha-reductase inhibitors (5ARI), correcting anatomic bladder outlet obstruction, intermittent catheterization, or bladder augmentation.

### Studies in patients with FI

Eleven studies examined 12 interventions for patients with FI (Additional file 2: Table S12 and S13), one study in women only, nine with more than 77% women, and one where the majority of included participants was male (85%).

Results are reported in Additional file 2: Table S14 and an overview of the summary of cure rates is reported in Table 4. All cure rates for all follow-up points within each study reported in the included studies are listed in Additional file 2: Table S14. The summary table (Table 4) lists all cure rates at the final follow-up for each study. In the text below, pooled results for each intervention are reported as specified in the Methods section.

**Table 4:** Cure rates^a^ in studies for female and male patients with fecal incontinence

Follow-up:	3 m	6 m	12 m	18 m	2 y	3 y	5 y
Treatment – WOMEN							
Biofeedback		NR					
Sacral neuromodulation	38.6%; 38.9%	31.7%; 39.3%	41.5%/47.2%; 40.6%	26%	37.3%	41.7% 4 y: 35.6%	36.1%
Peripheral stimulation	NR						
Methylcellulose + loperamide	46%						
Injectable bulking agents		NR	NR				
Standard conservative treatment	NR						
Treatment – MEN							
Biofeedback		40.8%				35.8%	29%

^a^Cure defined as ‘cure’, ‘objective cure’, or ‘100% improvement in incontinence episodes per week’

*NR* not reported

The median cure rate for female patients with FI following sacral neuromodulation was 38.6% (IQR, 35.6–40.6%). Methylcellulose plus loperamide was assessed in one study, with a cure rate of 46% at 3 months. In men, cure rates for biofeedback were 40.8% at 6 months, 35.8% at 3 years and 29% at 5 years’ follow-up.

No studies meeting inclusion criteria were found for the following interventions for FI: education of patient and/or caregiver, diet and eating pattern modifications, dietary fiber supplements, bowel habit training, rectal irrigation, continence products such as pads or anal plug for containment, PFMT, sphincteroplasty, artificial bowel sphincter, dynamic graciloplasty, antegrade continence enema, colostomy, magnetic anal sphincter, and puborectal sling.

### Studies in patients with incontinence due to neurological problems or diseases

Four studies were identified in patients with neurological problems or diseases. Of these, two studies included neurological patients with UI and the remaining two studies included those with FI (Additional file 2: Table S15 and S16).

Results are reported in Additional file 2: Table S17 and an overview of the summary of cure rates is reported in Table 5. All cure rates for all follow-up points within each study reported in the included studies are listed in Additional file 2: Table S17. The summary table (Table 5) lists all cure rates at the final follow-up for each study. In the text below, pooled results for each intervention are reported as specified in the Methods section.

**Table 5:** Cure rates^a^ in studies for female and male patients with neurological problems or diseases

Follow-up:	3 m	6 m	12 m	18 m	2 y	3 y	5 y
Treatment – WOMEN							
Botulinum toxin	NR						
Peripheral stimulation	NR						
Transanal irrigation				9%			
Voiding program	41%; 31%						
Usual continence care	30%						
Treatment – MEN							
None							

^a^Cure defined as ‘cure’, ‘objective cure’, ‘success (dry)’, or ‘100% improvement in incontinence episodes per week’

*NR* not reported

Only one study reported a cure rate beyond 3 months, showing 9% cure for FI at 18 months with transanal irrigation. Cure rates for urinary voiding programs (VP) were 41% (VP alone) and 31% (VP and supported implementation) at 3 months’ follow-up.

No studies were found for the following interventions for UI in patients with neurological problems or diseases: behavioral modification, external appliances, intermittent catheterization (with or without antimuscarinics), behavioral modification, antimuscarinics, continence products, indwelling catheter, alpha 1 adrenergic blockers, straining, triggered voiding, artificial sphincter, bladder neck sling, autologous sling, bladder neck closure, intraurethral stents, transurethral incision sphincter, sacral deafferentation, and enterocystoplasty.

No studies were found for the following interventions for FI in patients with neurological problems or diseases: containment products (such as anal plugs), patient education, adequate fiber diet and fluid intake, manual evacuation, mini-enema, digital rectal stimulation, chemical stimulant, fecal disimpaction, antegrade continence enema, graciloplasty, artificial anal sphincter, sacral anterior root stimulation, botulinum toxin, and neuromodulation.

### Studies in elderly or cognitively impaired patients with incontinence

Four trials were identified in elderly patients with incontinence; no studies were found for patients with cognitive impairments (Additional file 2: Table S18 and S19).

Results are reported in Additional file 2: Table S20 and an overview of the summary of cure rates is reported in Table 6. All cure rates for all follow-up points within each study reported in the included studies are listed in Additional file 2: Table S20. The summary table (Table 6) lists all cure rates at the final follow-up for each study. In the text below, pooled results for each intervention are reported as specified in the Methods section.

**Table 6:** Cure rates^a^ in studies for elderly or cognitively impaired patients with incontinence

Follow-up	3 m	6 m	12 m	18 m	2 y	3 y	5 y
WOMEN & MEN – Antimuscarinics							
Fesoterodine	50.8%	NR					
Darifenacin	48.5%; 32.7%						
WOMEN & MEN – Botulinum toxin							
Onabotulinumtoxin A	NR	NR	NR				

^a^Cure defined as ‘cure’, ‘objective cure’, ‘success (dry)’, or ‘100% improvement in incontinence episodes per week

*NR* not reported

None of the studies reported cure rates beyond 3 months’ follow-up. The only cure rates reported were 50.8% for fesoterodine and 33% and 49% for darifenacin for UI at 3 months’ follow-up.

No studies were found for the following interventions for UI or FI in older men and women: continence products, lifestyle interventions, behavioral therapies, and biofeedback. None of the included studies looked at cure or success rates for containment strategies.

## Discussion and conclusions

### Summary of main results

We included 98 individual studies evaluating 150 interventions. Five studies had more than 17 items with low risk of bias, indicating these are probably the most reliable studies; nine studies had fewer than 10 items with low risk of bias, probably indicating that these are the least reliable studies. However, these assessments were based on the available information for the study; a low score may reflect poor reporting (for example, due to lack of space in conference abstracts) rather than a poor study.

Surgical techniques for SUI resulted in a median cure rate of 82.3% (IQR, 72–89.5%). Patients with UUI were mostly treated pharmacologically, with a median cure rate for antimuscarinics of 49% (IQR, 35.6–58%), depending on the medicine used. MUI demonstrated results between SUI and UUI, depending on the nature of the underlying condition and the intervention employed.

For patients with FI, most studies evaluated sacral neuromodulation, which showed a median cure rate of 38.6% (IQR, 35.6–40.6%).

For patients with neurological problems or diseases, very few interventions were evaluated, with cure rates for FI of 9% for transanal irrigation at 18 months’ follow-up, and 31% and 41% for VPs.

For elderly patients with incontinence, only antimuscarinics were evaluated at 3 months’ follow-up, with cure rates of 50.8% for fesoterodine, and 32.7% and 48.5% for darifenacin. No studies were found for patients with cognitive impairments.

No studies were found that assessed the ability of containment strategies to support people with incontinence in leading normal lives.

### Treatment complications and adverse events

This systematic review focused on cure rates for the treatment of incontinence. However, it is also important to recognize that there are unintended consequences of the evaluated interventions, especially in light of the current debate of surgical meshes.

The European Union’s Scientific Committee on Emerging and Newly Identified Health Risks (SCENIHR) published an Opinion on the safety of surgical meshes used in urogynecological surgery in December 2015 [28]. The SCENIHR acknowledged the efficacy and use of implanted meshes for SUI in the majority of patients with moderate to severe SUI. It considered that the associated risk was limited, but recognized the absence of long-term data. Further, it commented that the risk of severe side effects (e.g. mesh exposure, shrinkage, pain) increases with the surface area of synthetic non-absorbable meshes, so that vaginally-implanted mesh for pelvic organ prolapse is associated with increased risks compared with mesh implantation for SUI.

Treatment of UUI was mainly with antimuscarinic drugs. These may cause a range of side effects such as dry mouth, gastrointestinal disturbances including constipation and flatulence, taste disturbances, blurred vision, dry eyes, drowsiness, dizziness, fatigue, difficulty in micturition (less commonly urinary retention), palpitation, and skin reactions [29]. Different antimuscarinic drugs differ in the level of adverse events seen [30]; only 14–35% of community dwelling patients still take antimuscarinic treatments 12 months after starting treatment [31].

Non-pharmacological treatments for UUI included PFMT. A Cochrane review [32] found only one trial reporting adverse events [33], comprising pain (one participant), uncomfortable feeling during exercise (three participants) and ‘not wanting to be continuously bothered with the problem’ (two participants).

Sacral neuromodulation for UUI can also cause adverse events, with 53% of patients in one trial [34] experiencing a range of adverse events. While this is a high rate of adverse events, it should be noted that the other studies cited in this discussion section examined different patient populations, making a direct comparison problematic.

Information on adverse events in treatments for FI was less extensive than for UI. Christensen et al. [35] carried out an audit of bowel perforations related to transanal irrigation using the Peristeen Anal Irrigation® system. A total of 49 such reports were found, corresponding to an average risk of bowel perforation of 6 per million procedures. In a review of 10 years of experience in a tertiary referral center between 2005 and 2015, out of 58 patients undergoing sacral neuromodulation system implantation Koh et al. [36] reported four postoperative complications (7%) including three wound infections and one lead migration, of which three required sacral nerve stimulation reinsertion. Adverse events of injectable bulking agents have also been described, including injection-site infection, fever, pain/proctalgia, prolonged defecation, and rectal hemorrhage [37].

### Strengths and limitations, including lack of evidence for missing interventions

The main strength of this review was an extensive search of the most relevant databases, conference proceedings and trial registers for a wide range of populations and interventions. A wide range of different outcomes on cure at different follow-up times, ranging from 3 months to 10 years was extracted making this one of the most complete overviews of cure rates for incontinence interventions to date.

Limitations included the lack of evidence for the specified populations for specific interventions (for instance, artificial sphincter for patients with UI) and for long-term follow-up (beyond 1 year). In some areas, evidence may have seemed limited as the bulk of relevant research was carried out before 2005 and was therefore excluded. In particular for FI, few studies could be included because trials are typically small (fewer than 50 participants), or ‘cure’ is often not reported, especially as most FI scores are composite and include flatus incontinence.

For patients with UI, the success of treatment depended greatly on the type of incontinence experienced, with treatment methods playing a significant part. For instance, for patients with SUI, the most common treatment were tapes and slings, with cure rates mostly between 60% and 90%, and for patients with UUI, the most common treatment was antimuscarinics, with cure rates mostly between 30% and 60%.

Furthermore, a critical factor to consider is the study population, for example, whether the population is treatment naïve or has failed prior therapy. In this review, all treatments were evaluated in line with their place in the treatment algorithms of the 5th ICI [22]. Therefore, for any second line therapies, such as botulinum toxin and sacral neuromodulation, patients will have failed primary therapy. Additionally, the diagnosis of mixed incontinence is often left to the discretion of the investigator, which may have led to differences between studies.

Additional limitations were the wide range of populations included in the studies, even within specific groups of patients, and the differences between interventions and differences between outcome measures reported. Where possible, we focused on reported objective cure rates. However, studies used different definitions of objective cure. For example, ‘completely dry’, ‘a negative cough stress test’, and ‘normal detrusor function during filling cystometry’ were all definitions used in patients with SUI.

### Overall conclusions

Most evidence was found for UI with a median cure rate of 82.3% (IQR, 72–89.5%) for surgical interventions for SUI. Cure rates with PFMT (ranging from 5% to 83.4% in all populations) and injectable bulking agents (24.8% and 36.9%) were lower. Most of the studies reporting cure with drugs were found for patients with UUI, almost exclusively with short-term follow-up (3 months) (median cure rate for antimuscarinics: 49%; IQR, 35.6–58%). For patients with FI, most studies evaluated sacral neuromodulation, revealing a median cure rate of 38.6% (IQR, 35.6–40.6%).

Thus, based on this systematic review, a large proportion of individuals treated for incontinence were not cured and may continue to rely on containment strategies, such as behavioral strategies and containment products. There was an absence of studies assessing the extent to which different types of behavioral and containment strategies supported people in leading a normal life. A number of pharmacological studies in UUI assessed reduction in pad use as a secondary outcome but none reported the extent of this reduction in the exposed population.

### Research/policy recommendations

Blinding of outcome assessors, random patient selection, and loss to follow-up were most often poorly reported, leading to risk of bias in the included studies. Future studies should report clearly on these items.

Very few combinations of interventions were found. Further evaluation of combination interventions for both UI and FI is warranted.

Evidence is lacking for interventions in people with incontinence due to neurological problems or diseases and for older adults and those persons with cognitive impairment, and research in these populations is therefore warranted.

Cure is important but there needs to be a better understanding of what types of containment strategies can best support a normal social life when cure is not achieved, and which types of containment strategy will be most beneficial for which type of person with incontinence. Further research regarding the ability of containment to improve the daily lives of individuals with incontinence is therefore required.

## Additional files

Additional file 1: Embase search strategy. (PDF 61 kb)

Additional file 2: Online tables. **Table S1.** Characteristics of included studies. **Table S2.** Risk of bias of included studies. **Table S3.** Study characteristics for female and male patients with stress urinary incontinence (SUI). **Table S4.** Treatment characteristics in studies for female and male patients with SUI. **Table S5.** Results of interventions for patients with SUI. **Table S6.** Study characteristics for patients with urgency urinary incontinence (UUI). **Table S7.** Treatment characteristics in studies for patients with UUI. **Table S8.** Results of interventions for patients with UUI. **Table S9.** Study characteristics for patients with mixed urinary incontinence (MUI). **Table S10.** Treatment characteristics in studies for patients with MUI. **Table S11.** Results of interventions for patients with MUI. **Table S12.** Study characteristics for patients with fecal incontinence (FI). **Table S13.** Treatment characteristics in studies for patients with FI. **Table S14.** Results of interventions for patients with FI. **Table S15.** Study characteristics for patients with neurological problems or diseases. **Table S16.** Treatment characteristics in studies for patients with neurological problems or diseases. **Table S17.** Results of interventions for patients with neurological problems or diseases. **Table S18.** Study characteristics for elderly or cognitively impaired patients with incontinence. **Table S19.** Treatment characteristics in studies for elderly or cognitively impaired patients with incontinence. **Table S20.** Results of interventions for elderly or cognitively impaired patients with incontinence. (PDF 4723 kb)
