# Supplementary material for: Can incontinence be cured? A systematic review of cure rates
Source: BMC Med. 2017 Mar 24;15:63. doi: 10.1186/s12916-017-0828-2 (PMC5364653; doi:10.1186/s12916-017-0828-2)
Supplement: Supplementary file 1 — Embase search strategy. (PDF 61 kb) [file 12916_2017_828_MOESM1_ESM.pdf]

Appendix – Embase Search Strategy

Embase (Ovid): 2005-2015/05/29
Searched 1.6.15

1 incontinence/ (11019)
2 continence/ (8496)
3 (incontinen$ or continen$).ti,ab,ot. (85054)
4 or/1-3 (88927)
5 urine incontinence/ or mixed incontinence/ or stress incontinence/ or urge incontinence/ (50343)
6 ((Urine$ or urinary or urinat$ or micturat$ or bladder$) adj4 (leak or leakage or leaks or leaking or seep or seepage or seeps or seeping or accident$ or escap$ or escaping or uncontrolled or trickl$ or "lack of control" or "no control" or "out of control" or "not voluntary" or involuntary or wetting or leaked or seeped)).ti,ab,ot. (5297)
7 (bladder$ adj3 control$).ti,ab,ot. (2941)
8 SUI.ti,ab,ot. (8640)
9 "giggle enuresis".ti,ab,ot. (2)
10 "enuresis risoria".ti,ab,ot. (10)
11 (incontinentia urinae or enuresis ureterica or ureter enuresis or enuresis diurnal).ti,ab,ot. (57)
12 ((Unable or inabilit$ or abilit$ or able) adj3 control$ adj3 (urine$ or urinat$ or urinary or micturat$)).ti,ab,ot. (29)
13 or/5-12 (60006)
14 neurogenic bladder/ (8712)
15 ((neurogenic$ or neurologic$ or spinal or spastic$) adj4 bladder$).ti,ab,ot. (6820)
16 neurogenic vesical dysfunct$.ti,ab,ot. (61)
17 (Bladder sphincter dys?ynergia or detrusor sphincter dys?ynergia or neurogenic detrusor overactiv$).ti,ab,ot. (1311)
18 or/14-17 (11895)
19 feces incontinence/ (14998)
20 (Encopresis or incontinentia alvi).ti,ab,ot. (816)
21 ((bowel$ or rectum or rectal$) adj4 (leak or leakage or leaks or leaking or seep or seepage or seeps or seeping or accident$ or escap$ or uncontrolled or trickl$ or "lack of control" or "no control" or "out of control" or "not voluntary" or involuntary or control$)).ti,ab,ot. (4581)
22 ((Unable or inabilit$ or abilit$ or able) adj3 control$ adj3 (faeces or faecal$ or feces or fecal$ or stool$ or rectum or rectal$ or bowel$ or bladder$ or anal$ or anus or urine or urinary or diarrh$ or soiling)).ti,ab,ot. (123)
23 ((feces or faeces or fecal$ or faecal$ or stool or stools or defecat$ or soiling) adj4 (leak or leakage or leaks or leaking or seep or seepage or seeps or seeping or accident$ or escap$ or escaping or uncontrolled or trickl$ or "not voluntary" or involuntary or control$)).ti,ab,ot. (3055)
24 ((diarrh$ or Pseudodiarrh$ or Pseudo-diarrh$) adj4 (leak or leakage or leaks or leaking or seep or seepage or seeps or seeping or accident$ or escap$ or uncontrolled or trickl$ or "not voluntary" or involuntary or control$)).ti,ab,ot. (2250)
25 ((Unable or inabilit$ or abilit$ or able) adj3 control$ adj3 (diarrh$ or Pseudodiarrh$ or Pseudo-diarrh$)).ti,ab,ot. (1)
26 or/19-25 (24037)
27 or/4,13,18,26 (132591)
28 diaper/ or absorbent pad/ or hygiene product/ or adult diaper/ or (protective$ adj3 (underwear$ or under-wear$ or underpant$ or under-pant$ or shield or shields or undergarment$ or under-garment$ or briefs or brief or pant or pants or panties or panty$ or leaf or leaves or pouch$ or guard or guards or system$ or slip or slips)).ti,ab,ot. (3905)

29 ((incontinen$ or Continen$) adj3 (product$ or aid$ or accessory or accessories or underwear$ or under-wear$ or knicker$ or pant or pants or underpant$ or shield or shields or undergarment$ or under-garment$ or briefs or brief or device$ or strateg$)).ti,ab,ot. (793)
30 ((Pant or pants or underwear$ or under-wear$ or underpant$ or shield or shields or undergarment$ or under-garment$ or briefs or brief) adj3 (liner$ or pad or pads or absorben$)).ti,ab,ot. (78)
31 (diaper$ or underpad$ or under-pad$ or pull-up or pull-ups or T-shaped pad$ or belted pad$ or belted slip$ or sanitary pad$ or menstrual pad$ or pantliner$ or pant-liner$).ti,ab,ot. (2965)
32 ((throwaway$ or throw-away$ or flushable$ or disposable or single-us$ or reuse$ or re-use$ or washable or absorbent$) adj3 (pad or pads or padding or diaper$ or insert or inserts or underpad$ or liner$ or product$ or underwear$ or under-wear$ or underpant$ or under-pant$ or shield or shields or undergarment$ or under-garment$ or briefs or brief)).ti,ab,ot. (1038)
33 exp Incontinence aid/ (201)
34 ((incontinen$ or Continen$) adj3 (aid or aids or aiding or helping or help or assist$ or alleviat$ or remed$ or benefit$ or relief or reliev$ or comfort$)).ti,ab,ot. (801)
35 ((Collecting or collection or fixer-occluder) adj2 (system$ or device$ or product$ or appliance$ or apparatus)).ti,ab,ot. (9017)
36 (((intermittent$ or Indwell$ or In-dwell$) adj2 catheter$) or foley$).ti,ab,ot. (15233)
37 (personal urine adj4 (drain$ or system$ or device$ or product$ or appliance$ or apparatus)).ti,ab,ot. (0)
38 (U-Drain or (containment$ adj2 (strateg$ or device$ or product$))).ti,ab,ot. (607)
39 InterStim.ti,ab,ot. (265)
40 sacral nerve stimulation/ (1174)
41 (Sacral nerve stimulat$ or Percutaneous tibial nerve stimulat$ or posterior tibial nerve stimulat$).ti,ab,ot. (1470)
42 (SNS or SNM or PTNS).ti,ab,ot. (5088)
43 (sacral adj3 (neuromodulat$ or neuro-modulat$ or deafferent$ or de-afferent$)).ti,ab,ot. (1039)
44 (medical electrical stimulation therap$ or Enterocystoplast$ or Entero-cystoplast$ or ((bladder$ or sacral$) adj2 (Autoaugment$ or Auto-augment$))).ti,ab,ot. (744)
45 (PTQ implant$ or ((bio-compatible or biocompatible or inject$) adj2 bulking agent$)).ti,ab,ot. (284)
46 muscle training/ or muscle strength/ or pelvic floor muscle training/ (43460)
47 exp feedback system/ or Electrostimulation Therapy/ or Electrostimulation/ (164186)
48 behavior therapy/ (38263)
49 (perineomet$ or biofeedback or bio-feedback or kegel$ or myofeedback or myo-feedback).ti,ab,ot. (7962)
50 (feedback adj2 (sensory or delay$ or control$ or loop or loops or regulat$ or biochemical$ or bio-chemical$ or mechanism$ or physiolog$)).ti,ab,ot. (33377)
51 (Electromyograph$ or Electro-myograph$ or EMG).ti,ab,ot. (58479)
52 ((pelvi$ adj3 rehab$) or ((rectal$ or rectum or anus or anal$ or transanal$ or trans-anal$ or transrect$ or trans-rect$) adj1 (irrigat$ or stimulat$)) or (manual$ adj3 evacuat$) or (minienema$ or mini-enema$)).ti,ab,ot. (1743)
53 (electrostimulat$ or electrotherap$ or electro-therap$ or electro-stimulat$ or galvanostimulat$ or galvano-stimulat$).ti,ab,ot. (5708)
54 ((pelvi$ or muscle$ or bladder$) adj4 (train$ or exercis$ or educat$ or reeducat$ or rehab$ or therap$ or retrain$ or relax$)).ti,ab,ot. (54732)
55 ((behavio?r$ or cognit$ or exercis$ or lifestyle$ or life-style$ or educational$) adj4 (therap$ or train$ or treat$ or strateg$ or interven$ or method$ or manag$ or program$ or regime$)).ti,ab,ot. (235778)
56 ((urg$ adj3 suppress$) or assisted toilet visit$).ti,ab,ot. (111)

57 (frequenc$ adj4 strateg$).ti,ab,ot. (901)
58 ((time or timed or timing or schedule$) adj2 (void$ or urinat$)).ti,ab,ot. (660)
59 ((weight$ or device$ or cone$) adj4 vagin$).ti,ab,ot. (1198)
60 (pfmt or pfe or pfx).ti,ab,ot. (885)
61 (physical therap$ or physiotherap$ or physio-therap$).ti,ab,ot. (47798)
62 ((conservative or educat$) adj5 (treat$ or therap$ or program$ or manag$ or strateg$ or interven$ or method$)).ti,ab,ot. (179216)
63 ((liquid$ or fluid$ or drink$) adj4 (intak$ or manipulat$ or reduc$ or control$)).ti,ab,ot. (32493)
64 duloxetine/ or (Duloxetine or Cymbalta or duzela or ly-248686 or ly248686 or 116539-59-4 or 136434-34-9).ti,ab,ot,hw,tn,rn. (7446)
65 Fesoterodine/ or (Fesoterodine or Toviaz or spm907 or spm-907 or 286930-02-7).ti,ab,ot,hw,tn,rn. (587)
66 Tolterodine/ or (Tolterodine or Detrol or Detrusitol or pnu-200583 or pnu200583 or 124937-51-5).ti,ab,ot,hw,tn,rn. (3161)
67 Oxybutynin/ or (Oxybutynin or Anturol or cystonorm or cystrin or delifon or ditropan or Ditropan-xl or diutropin or dridase or driptane or esoxybutynin or frenurin or gelnique or iliaden or kentera or kl-007 or kl007 or lenditro or lyrinel or mj-4309-1 or mj-43091 or mj43091 or mj4309-1 or mutum-cr or nefryl or novitropan or oxibutinin or oxyban or oxybutynin or oxytrol or oyrobin or pollakis or reteven or tropan or uricont or uroflax or urotrol or zatur-ge or 119618-22-3 or 1508-65-2 or 230949-16-3 or 5633-20-5).ti,ab,ot,hw,tn,rn. (5138)
68 muscarinic receptor blocking agent/ or (Antimuscarinic$ or Anti-muscarinic$ or muscarinolytic$).ti,ab,ot. (8685)
69 (Muscarinic adj3 (antagonist$ or blocker$ or blocking)).ti,ab,ot. (6606)
70 Dicycloverine/ or (Dicycloverine or Atumin or Byclomine or babyspasmil or balacon or benacol or bentyl or bentylol or benulone or clomin or cyclominol or dibent or diclomin or dicomin or dicyclocot or dicyclomine or dicymine or dilomin or Di-Spaz or Dilomine or diocyl or dyspas or esentil or formulex or incron or jl998 or jl-998 or kolantyl or lomine or m-33536 or m33536 or magesanp or magesan-p or medicyclomine or merbentyl or neoquess or nomcramp or notensyl or panakiron or respolimin or spasmotine or swityl or wyovin or 50815-09-3 or 67-92-5 or 77-19-0).ti,ab,ot,hw,rn,tn. (1402)
71 Flavoxate/ or (Ak-123 or ak123 or baduson or bladderon or bladuril or cleanxate or dw-61 or dw61 or flavate or flavo-spa or flavorin or flavoxate or fucotin or genurin or harnin or nsc114649 or nsc-114649 or rec-7-0040 or rec7-0040 or rec70040 or rec-70040 or spagerin or spasdic or spasuret or spasuri or tonlin or urispadol or Urispas or uritac or uronid or uropeace or uroxate or voxate or yungken or 15301-69-6 or 3717-88-2).ti,ab,ot,hw,tn,rn. (789)
72 Solifenacin/ or (solifenacin or vesicare or vesikur or ym53705 or ym-53705 or ym-905 or ym905 or 180272-14-4 or 180272-16-6 or 180468-39-7).ti,ab,ot,hw,tn,rn. (1529)
73 Darifenacin/ or (darifenacin or emselex or enablex or uk-88525 or uk-88525-04 or uk88525-04 or uk88525 or uk-88525-04 or 133099-04-4 or 133099-07-7).ti,ab,ot,hw,tn,rn. (1176)
74 Hyoscyamine/ or (a-spas or anaspaz or buwecon or cystospaz or cytospaz or daturine or donnamar or duboisine or egacen or egazil or hyomax or hyoscamine or hyosciamine or hyoscyamine or hyoscyanin or hyosyne or ib-stat or levbid or levsin or levsinex or neosol or neoquess or nulev or spacol or spasdel or symax or tropine-l-tropate or 101-31-5 or 306-03-6).ti,ab,ot,hw,tn,rn. (1735)
75 Trospium chloride/ or ("Trospium chloride" or Regurin or sanctura or spasmex or spasmo-urgenin or spasmo-lyt or spasmolyt or spasmourgenin or trosec or flotros or 10405-02-4).ti,ab,ot,hw,tn,rn. (1235)
76 Mirabegron/ or (mirabegron or betanis or betmiga or myrbetriq or sc-211912 or sc211912 or ym-178 or ym178 or 223673-61-8).ti,ab,ot,hw,tn,rn. (453)
77 Imidafenacin/ or (Imidafenacin or Krp-197 or krp197 or ono-8025 or ono8025 or 170105-16-5).ti,ab,ot,hw,tn,rn. (146)

78 Loperamide/ or (Loperamide or immodium or imodium or lopex or acanol or adl-2-1294 or adl2-1294 or adl21294 or adl-21294 or amerol or betaperamide or binaldan or colifilm or colodium or desitin or diacure or diadium or diapen or diarem or diarent or diarex-lactab or diarin or diarlop or diarodil or diarr-eze or diarreze or diarreeze or diarstop or diasolv or dicap or dissenten or donafan or elcoman or ercestop or fortasec or gastro-stop or glubemide or imosec or imosen or imossel or imotril or lenide or lodia or loniper or lop-dia or lopamid or lopamide or lopedin or lopemid or lopemin or loperacap or loperamil or loperastat or loperhoe or loperid or loperium or lopermide or loperol or loperyl or lopetrans or lorpa or nabutil or nimaz or oramide or orulop or pangetan or perasian or pramidal or prodium or r-18-553 or r-18553 or r18-553 or r18553 or raxedin or regulane or rexamide or sanpo or seldiar or stopit or suprasec or tanitril or tebloc or top-dal or undiarrhea or undiarrhoea or vacontil or 53179-11-6 or 34552-83-5).ti,ab,ot,hw,tn,rn. (7216)
79 botulinum toxin A/ or (((botulin$ or abobotulinumtoxin or BoNT or evabotulinum$ or incobotulinum$ or onabotulinum$) adj3 "A") or abobotulinumtoxinA or azzalure or bocouture or botox or BTXA or "clostridium botulinum a toxin" or "clostridium botulinum endotoxin" or dyslor or dysport or "toxin A" or incobotulinumtoxinA or nt-201 or nt201 or oculinum or onabotulinumtoxinA or onaclostox or prosigne or purtox or reloxin or vistabel or vistabex or xeomin or 1309378-01-5 or 93384-43-1).ti,ab,ot,tn,rn. (20527)
80 exp suburethral sling/ (4934)
81 ((Sling or slings or minisling$) adj2 (surg$ or procedure$ or interven$ or device$ or system$ or appliance$ or apparatus or tape$ or male$)).ti,ab,ot. (2530)
82 (TVT or REMEEX).ti,ab,ot. (2378)
83 (("one cut" or "one incision" or single incision or single cut or needleless or needle-less or needlefree or needle-free or procedure$ or sling$ or technique$) adj3 TOT).ti,ab,ot. (426)
84 (Re-adjustable Mechanical External or Readjustable Mechanical External).ti,ab,ot. (0)
85 (Bladder adj2 (reposition$ or re-position$ or realign$ or re-align$)).ti,ab,ot. (26)
86 (Marshall-Marchetti-Krantz or MMK or retropubic suspen$ or retro-pubic suspen$ or bladder neck suspension surger$ or colposuspen$ or colpo-suspen$).ti,ab,ot. (1564)
87 ((urethropex$ or urethrocystopex$ or urethra-pex$ or urethra-cystopex$) adj4 (tension or tape$)).ti,ab,ot. (9)
88 ((vagina$ or transvagina$ or trans-vagina$) adj2 (tape$ or tension$ or sling or slings)).ti,ab,ot. (2284)
89 ((tension adj3 tape$) or slingplast$ or sling-plast$).ti,ab,ot. (1918)
90 ((suburethral$ or sub-urethral$ or midurethral$ or mid-urethral$ or tension-free or tensionfree or retropubic$ or retro-pubic$ or suprapubic$ or supra-pubic$ or trans-obturator$ or transobturator$) adj3 (tape$ or sling$)).ti,ab,ot. (4487)
91 (SPARC adj3 (tape$ or procedure$ or interven$ or sling or slings)).ti,ab,ot. (90)
92 (Monarc or Obtape or Uratape).ti,ab,ot. (241)
93 exp feces incontinence device/ (32)
94 sphincteroplasty/ (842)
95 (rectamid or Sphincteroplast$ or Sphinctero-plast$ or sphincter repair$).ti,ab,ot. (1232)
96 (antegrade colonic irrigat$ or antegrade continence enema$ or Malone procedure$ or percutaneous cecostom$ or antegrade incontinence enema$).ti,ab,ot. (305)
97 (Anal adj2 (plug$ or encircl$)).ti,ab,ot. (267)
98 (Thiersch adj3 (procedure$ or operat$ or surger$ or interven$ or method$)).ti,ab,ot. (76)
99 (((nondynamic or non-dynamic or dynamic) adj3 (graciloplast$ or gracilo-plast$)) or "bio-Thiersch").ti,ab,ot. (159)
100 ((Post-anal$ or Postanal$) adj3 (repair$ or restor$)).ti,ab,ot. (110)
101 bladder sphincter prosthesis/ or urinary prosthesis/ (1303)
102 (artificial$ adj3 (bladder$ or sphincter$ or neosphincter$ or neo-sphincter$)).ti,ab,ot. (2073)
103 "AMS Sphincter".ti,ab,ot. (8)
104 ((bladder$ or sphincter$) adj3 prosthe$).ti,ab,ot. (185)

105 ((Radiofrequen$ or Radio-frequen$) adj3 ablat$).ti,ab,ot. (20663)
106 SECCA.ti,ab,ot. (57)
107 ((diversion$ or divert$ or stoma or stomas or ileostom$ or colostom$) adj3 (faeces or feces or Fecal$ or faecal$ or stool or stools)).ti,ab,ot. (1104)
108 or/4,13,18,26,29,33-35,37-39,45,80-104,106 (145084)
109 or/28,30-32,36,40-44,46-79,105,107 (856072)
110 27 and 109 (25059)
111 108 or 110 (145084)
112 animal/ or animal experiment/ (3498566)
113 (rat or rats or mouse or mice or murine or rodent or rodents or hamster or hamsters or pig or pigs or porcine or rabbit or rabbits or animal or animals or dogs or dog or cats or cow or bovine or sheep or ovine or monkey or monkeys).ti,ab,ot,hw. (5932991)
114 or/112-113 (5932991)
115 exp human/ or human experiment/ (15965554)
116 114 not (114 and 115) (4703807)
117 111 not 116 (135160)
118 treatment response/ (147362)
119 "evaluation and follow up"/ (2679)
120 minimal residual disease/ (14211)
121 ((Success$ adj2 manag$) or (Continu$ adj3 pad$ adj3 (use$ or usage$ or using))).ti,ab,ot. (20736)
122 recurrence risk/ (40944)
123 (Rehab$ adj2 outcome$).ti,ab,ot. (3180)
124 ((Treatment$ or intervention$ or therap$ or procedur$) adj2 (success or unsuccessful$ or fail$ or ineffective$ or resolv$ or resolution$ or solv$ or solution$)).ti,ab,ot. (104805)
125 ((cure$ or Success$ or fail$ or recur$ or Relaps$ or resolv$ or resolut$ or rehabil$ or unsuccessful$ or resolution$ or solv$ or solution$ or ineffect$) adj2 (rate or rates)).ti,ab,ot. (164156)
126 ((dysfunction$ or Function$) adj1 (outcome$ or effect$)).ti,ab,ot. (56014)
127 or/118-126 (504949)
128 117 and 127 (11702)
129 limit 128 to yr="2005 -Current" (8890)

Selected TVT terms based on:

Cody J, Wyness L, Wallace S, Glazener C, Kilonzo M, Stearns S, et al. Systematic review of the clinical effectiveness and cost-effectiveness of tension-free vaginal tape for treatment of urinary stress incontinence. Health Technol Assess. 2003/09/19 ed, 2003: iii, 1-189.
