# Supplementary material for: Can incontinence be cured? A systematic review of cure rates
Source: BMC Med. 2017 Mar 24;15:63. doi: 10.1186/s12916-017-0828-2 (PMC5364653; doi:10.1186/s12916-017-0828-2)
Supplement: Supplementary file 2 — Online tables. Table S1. Characteristics of included studies. Table S2. Risk of bias of included studies. Table S3. Study characteristics for female and male patients with stress urinary incontinence (SUI). Table S4. Treatment characteristics in studies for female and male patients with SUI. Table S5. Results of interventions for patients with SUI. Table S6. Study characteristics for patients with urgency urinary incontinence (UUI). Table S7. Treatment characteristics in studies for patients with UUI. Table S8. Results of interventions for patients with UUI. Table S9. Study characteristics for patients with mixed urinary incontinence (MUI). Table S10. Treatment characteristics in studies for patients with MUI. Table S11. Results of interventions for patients with MUI. Table S12. Study characteristics for patients with fecal incontinence (FI). Table S13. Treatment characteristics in studies for patients with FI. Table S14. Results of interventions for patients with FI. Table S15. Study characteristics for patients with neurological problems or diseases. Table S16. Treatment characteristics in studies for patients with neurological problems or diseases. Table S17. Results of interventions for patients with neurological problems or diseases. Table S18. Study characteristics for elderly or cognitively impaired patients with incontinence. Table S19. Treatment characteristics in studies for elderly or cognitively impaired patients with incontinence. Table S20. Results of interventions for elderly or cognitively impaired patients with incontinence. (PDF 4723 kb) [file 12916_2017_828_MOESM2_ESM.pdf]

Table S1: Characteristics of included studies

STUDY ID	Population	N	Country	Intervention(s)
#15549. Wagg 2014[23]	Elderly patients with overactive bladder	305	EU (Portugal; Finland; Germany; Slovakia; UK; Denmark; Belgium; Spain; Italy; Austria; Sweden)	Fesoterodine (antimuscarinic)
#11920 Chapple 2007a[24]	Elderly patients with overactive bladder	266	USA, Poland, South Africa, Hungary, Sweden, UK, Germany	Darifenacin (antimuscarinic)
# 15558 Dubeau 2014[25]	Elderly	562	USA	Fesoterodine (antimuscarinic)
#15559 Liao 2013[26]	Elderly, Frail elderly and younger than 65y (UUI)	166	Taiwan	OnabotulinumtoxinA
#2918. Wexner 2010[27]	Faecal incontinence	129	NR	Sacral nerve stimulation
#6392. Paquette 2014[28]	Faecal incontinence	111	NR	Sacral nerve stimulation
#10132. Graf 2011[29]	Faecal incontinence	206	Sweden, USA, Germany	Dextranomer (biomaterial to inject in anal submucosal)
#9773. Leroi 2012[16]	Faecal incontinence (neurological and non-neurological)	73	France	Transcutaneous electrical tibial nerve stimulation
#2779. Lacima 2010[30]	Faecal incontinence in non-neurological patients	79	Spain	Biofeedback
#3161. Sze 2009[31]	Faecal incontinence in non-neurological patients	59	USA	Methylcellulose and loperamide
#3856. Byrne 2007[32]	Faecal incontinence in non-neurological patients	385	Australia	Biofeedback
#11675. Tjandra 2008[33]	Faecal incontinence in non-neurological patients	120	Australia	Sacral Nerve Stimulation; Optimal medical therapy (bulking agents, pelvic floor exercises with a team of dedicated physiotherapists, and dietary management on fluid and fibres with a team of dieticians)

STUDY ID	Population	N	Country	Intervention(s)
#1373. Damon 2014[34]	Faecal incontinence non-neurological	157	France	Standard conservative treatment; perineal retraining (biofeedback plus standard conservative treatment)
#2304. Uludag 2011[35]	Faecal incontinence non-neurological	50	Netherlands	Sacral neuromodulation
#2320. Boyle 2011[36]	Faecal incontinence non-neurological	50	UK	Sacral Nerve Stimulation
#13213. Faaborg 2009[37]	Faecal incontinence in neurological patients	211	Denmark	Transanal colonic irrigation
#15557Thomas 2014[38]	Urinary incontinence in neurological patients	330	UK	Systematic voiding programme (SVP), SVP and supported implementation and Usual continence care
#11682Sussman 2013[39]	Urinary incontinence in neurological patients	166	USA; Italy; Canada	OnabotulinumtoxinA 200U or 300U
#11558. Staskin 2006[40]	MUI and overactive bladder	319	USA	Solifenacin 5mg or 10mg (antimuscarinic)
#13156. Glazener 2014[41]	Unspecified postnatal UI, MUI	230	UK	Pelvic floor muscle training + bladder training
#11474. Moore 2008[42]	Unspecified UI postprostatectomy, MUI	106	Canada	Weekly postoperative pelvic floor muscle training (PFMT)
#11477. Manassero 2007[43]	Unspecified UI postprostatectomy, MUI	152	Italy	Early, intensive, prolonged pelvic floor exercises (PFE)
#11505. Williams 2005[44]	Unspecified urinary incontinence, MUI	2240	UK	Nurse-led continence Service
#13488 Astellas Pharma[45]	MUI (post-prostatectomy)	313	USA, Canada	Solifenacin (antimuscarinic)
#4495. Abdel-Hady 2005[46]	MUI, UUI, SUI	454	UK	Tension-free vaginal tape
#1199. Schweitzer 2015[47]	SUI	156	Netherlands	Adjustable Single-Incision sling; Transobturator Sling
#1334. Zuckerman 2014[48]	SUI	102	USA	Transobturator sling (AdVance)
#1538. Mostafa 2013[49]	SUI	137	UK	Single-incision Mini-sling; Tension-free Vaginal Tape-obturator
#1571. Serati 2013[50]	SUI	263	Italy	Tension Free Vaginal Tape (Obturator) (TVT-O);

STUDY ID	Population	N	Country	Intervention(s)
				antimuscarinic (unspecified)
#1710. Tommaselli 2013[51]	SUI	110	Italy	Traditional vs. modified technique for TVT-O
#1728. Tommaselli 2013[52]	SUI	154	Italy	Tension-Free Vaginal Tape-O (TVT-O);
#1904. Abdel-Fattah 2012[53]	SUI	341	UK	Inside-out (TVT-O) vs outside-in (TOT-Aris) transobturator tension-free vaginal tape (TO-TVT)
#1969. Cornu 2012[54]	SUI	95	France, USA	Adjust single incision transobturator sling procedure
#2258. Neuman 2011[55]	SUI	162	Israel	Tension-free vaginal tape–obturator (TVT-O) suburethral or mid-urethral tape operation.
#2490. Bochove 2011[56]	SUI	95	Netherlands	Argus adjustable sling
#2702. Guerrero 2010[57]	SUI	201	UK	TVT-TM, and autologous fascial slings
#2950. Houwert 2010[58]	SUI	437	Netherlands	Retropubic tension-free vaginal tape (TVT); transobturator tape (TOT) [including 2: Monarc, and tension-free vaginal tape obturator].
#2953. Krofta 2010[59]	SUI	300	Czech Republic	Tension-free vaginal tape (TVT); inside-out transobturator midurethral sling TVT-O
#2955. North 2010[60]	SUI	60	UK	Single incision sling procedure (Minitape-TM)
#3257. Liebergall 2009[61]	SUI	245	Israel	Circular muscle exercises (Paula method); Pelvic Muscle Floor Training
#3620. Barry 2008[62]	SUI	187	Australia	Transobturator tape (Monarc®); tension-free vaginal tape (TVT®)
#3630. Ward 2008[63]	SUI	344	UK	Tension-free vaginal tape ; colposuspension
#3914. Harms 2007[64]	SUI	177	Germany	Tension-free vaginal tape (TVT)
#5274. Lee 2015[65]	SUI	225	Australia	Single incision midurethral sling (Miniarc); outside in transobturator midurethral sling (Monarc)
#9658. Van Leijsen 2013[66]	SUI	126	Netherlands	Immediate midurethral sling surgery; individually tailored therapy ("state-of-the-art")
#9689. Serra 2013[67]	SUI	61	Spain	Advance and Advance XP transobturator male sling
#9798. Masata 2012[68]	SUI	197	Czech Republic	Tension-free vaginal tape obturator (TVT-O)
#11686. Carey 2006[69]	SUI	200	Australia	Laparoscopic Burch Colposuspension; Open Burch colposuspension

STUDY ID	Population	N	Country	Intervention(s)
#13090. Groutz 2011[70]	SUI	61	Israel	Tension-free vaginal tape
#13133. Labrie 2013[71]	SUI	460	Netherlands	Pelvic floor muscle training; midurethral sling surgery
#2539. Freeman 2011[72]	SUI	193	UK	Retropubic (TVT) versus trans-obturator (TOT) mid-urethral slings
#15548. Sjostrom 2015[73]	SUI	155	Sweden	Internet-based PFMT<br>Postal PFMT
#14621. NCT01272284 2011[74]	Female SUI	112	USA, Canada	Altis® Single Incision Sling System (SIS)
#1656 Lee 2013[75]	SUI	56	South Korea	Extracorporeal biofeedback combined with pelvic floor muscle training (PFMT)
#10965 Viktrup 2007[76]	SUI	958	Africa, Australia, Europe, North and South America	Duloxetine
#10419. Lanoe 2009[77]	Male SUI	84	France	Male sling (InVance)
#3326. Ghoniem 2009[78]	SUI + intrinsic sphincter deficiency	147	USA, Canada	Transurethral injection of bulking agents Macroplastique or Contigen®.
#2053. Rehder 2012[79]	SUI postprostatectomy	156	Austria, France, Italy, Germany	Transobturator Retroluminal Repositioning Sling Suspension (AdVance)
#2979. Bauer 2009[80]	SUI postprostatectomy	113	Germany	Functional Sling Suspension AdVance
#11513. Filocamo 2007[81]	SUI postprostatectomy	50	Italy	Duloxetine
#3822. Kondo 2007[82]	SUI, MUI	123	Japan	Pelvic Floor Muscle Training
#4000. Primus 2006[83]	SUI, MUI	103	Austria	The SPARC Female Sling
#4019. Williams 2006[84]	SUI, MUI	231	UK	pelvic floor muscle therapies (PFMT)
#4664. Natale 2014[85]	SUI, MUI	92	Italy	Single incision sling (AjustTM)
#4757. Palomba 2014[86]	SUI, MUI	209	Italy	Single-incision mini-sling; retropubic tension-free vaginal tape
#4991. Dias 2014[87]	SUI, MUI	50	Portugal	Altis® single-incision sling procedure
#11523 Schagen van Leeuwen 2008[88]	SUI/MUI (women of ≥65 years)	126	Germany; France; The Netherlands; Spain; Sweden; Switzerland;	Duloxetine

STUDY ID	Population	N	Country	Intervention(s)
			South-Africa.	
#11411. Marchiori 2010[89]	UI post prostatectomy, SUI	332	Italy	Post surgery tutored and personal trained pelvic floor re-educational program; pelvic floor exercises performed by patients on their own.
#11577. Haab 2006[90]	Overactive bladder, UUI	466	France, Canada, USA, Denmark, Australia, Switzerland.	Darifenacin (antimuscarinic)
#11438. Tincello 2012[91]	Overactive bladder and Refractory detrusor overactivity, UUI	100	UK	OnabotulinumtoxinA
#4293. Amundsen 2005[92]	UUI	55	USA	Sacral neuromodulation
#4552. Siegel 2015[93]	UUI	147	USA	Sacral neuromodulation (SNM); standard medical therapy with antimuscarinics (SMT)
#13577. New England Research Inst 2013[94]	UUI	118/119	USA	Tolterodine (antimuscarinic) + Behavioural training
#2358. Groen 2011[95]	UUI (refractory idiopathic)	60	Netherlands	Sacral Neuromodulation
#11496. Rogers 2009[96]	UUI and overactive bladder	161	USA	Tolterodine ER (antimuscarinic)
#10558. Surwit 2009[97]	UUI, MUI	256	USA	Percutaneous tibial nerve neuromodulation + pelvic floor muscle rehabilitation
#4217. Duckett 2006[98]	UUI, SUI	51	UK	Tension-free vaginal tape
#847. Glazener 2011[99]	Unspecified urinary incontinence, MUI	788	UK	PFMT (after Radical Prostatectomy)<br>Lifestyle advice (after Radical Prostatectomy)<br>PFMT (after TURP)<br>Lifestyle advice (after TURP)
#11434. Chapple 2013[100]	UUI	2382	Europe, United States, Canada, South Africa, Australia, and New Zealand	Mirabegron 50 mg and 100 mg (Adrenergic) and Tolterodine ER 4 mg (antimuscarinic)
#15546. Drake 2015[101]	UUI	1004	NR/Unclear	Solifenacin 6mg (antimuscarinic) and Tamsulosin 0.4mg

STUDY ID	Population	N	Country	Intervention(s)
#15547. Visco 2012[102]	UUI	119	USA	Anticholinergic drug
#9683 Nitti 2013[103]	UUI	280	USA; Canada	OnabotulinumtoxinA
#11452 Rovner 2011[104]	UUI	269	USA; Canada; Europe	OnabotulinumtoxinA 50U, 100U, 150U, 200U and 300U
#11509 Herschorn 2010[105]	UUI	1277	NR	Tolterodine ER and Fesoterodine (antimuscarinic)
#15555 Chapple 2014[106]	UUI	1955	Worldwide (27 countries)	Fesoterodine 4mg and 8mg (antimuscarinic)
#11120 Anderson 2006[107]	UUI	790	USA	Oxybutynin and Tolterodine ER (antimuscarinic); with or without prior anticholergic therapy
#15552 Herschorn 2013[108]	UUI	511	North America, Europe	Mirabegron 25 mg and 50 mg (Adrenergic)
#11576 Chapple 2005[109]	UUI	1177	Europe	Solifenacin and Tolterodine ER (antimuscarinic)
#10668 Chapple 2007[110]	UUI	824	Europe, South Africa, Australia, and New Zealand	Tolterodine ER and Fesoterodine 4mg and 8mg (antimuscarinic)
#14046 Janssen Korea 2013[111]	OAB+UUI	309	South Korea	Oxybutynin (antimuscarinic)
#6213 Sand 2012[112]	OAB +UUI	280	USA	OnabotulinumtoxinA
#10091 Staskin 2011[113]	UUI	168	Worldwide	Fesoterodine (antimuscarinic)
#10655 Karram 2009[114]	OAB+UUI	229	USA	Solifenacin (antimuscarinic)
#10579 Wyndaele 2009[115]	OAB+UUI	474	Asia, Europe, and North and Central America	Fesoterodine (antimuscarinic)
#11732 Yamaguchi 2007[116]	OAB+UUI	713	Japan	Solifenacin 5mg or 10mg (antimuscarinic)
#10812 Dmochowsk i 2008[117]	UUI	267	USA	Trospium (antimuscarinic)
#11473 Kaplan 2011[118]	UUI	1834	North America, South America, Europe, Asia and Africa	Tolterodine ER and Fesoterodine (antimuscarinic)
#15553 Staskin 2007[119]	UUI	292	USA	Trospium (antimuscarinic)

Table S2: Risk of bias of included studies

Study name	Endnote	Questions																				No. "L"	No. "H"	No. "U"
		1	2	3	4	5	6	8	9	11	12	13	14	15	16	19	20	23	25	26	28			
Abdel-fattah 2012[53]	#1904	L	L	L	L	U	U	U	H	L	U	L	U	H	L	U	L	U	U	H	L	9	3	8
Abdel-Hady 2005[46]	#4495	L	L	L	L	H	H	L	L	U	U	L	U	H	L	U	H	H	U	L	L	10	4	6
Amundsen 2005[92]	#4293	L	L	L	L	U	U	L	L	L	H	L	U	H	L	L	L	H	U	L	L	13	3	4
Anderson 2006[107]	#11120	L	U	L	L	L	H	L	L	U	L	L	U	U	H	U	U	U	U	H	L	9	3	8
Astellas Pharma 2014[45]	#13488	L	L	L	U	L	L	L	L	U	L	U	L	L	L	U	L	U	U	U	L	13	0	7
Barry 2008[62]	#3620	L	L	L	L	L	L	H	L	U	L	L	U	H	L	U	H	L	U	L	L	13	3	4
Bauer 2009[80]	#2979	L	L	L	L	H	L	H	L	U	L	L	U	H	L	U	L	H	U	L	L	12	4	4
Bochove-Overg. 2011[56]	#2490	L	L	L	L	U	L	L	L	U	L	L	U	H	L	U	L	H	U	L	L	13	2	5
Boyle 2011[36]	#2320	L	L	L	L	U	L	H	L	U	L	L	U	H	L	U	L	H	U	L	L	12	3	5
Byrne 2007[32]	#3856	L	L	L	L	L	L	H	L	U	L	L	U	L	L	L	H	H	U	L	L	14	3	3
Carey 2006[69]	#11686	L	L	L	L	L	L	L	L	U	L	L	L	L	L	L	L	L	L	L	L	19	0	1
Chapple 2005[109]	#11576	L	L	H	L	U	H	L	U	U	L	L	L	L	L	U	L	L	U	H	L	12	3	5
Chapple 2007a[24]	#11920	L	L	L	L	L	L	L	L	U	L	L	L	L	H	U	L	U	U	L	L	15	1	4
Chapple 2007b[110]	#10668	L	L	L	L	L	U	L	U	U	L	L	L	L	H	U	L	U	U	H	L	12	2	6
Chapple 2013[100]	#11434	L	L	L	U	L	L	L	L	U	L	L	L	L	L	L	L	L	L	L	L	18	0	2
Chapple 2014[106]	# 15555	L	L	L	L	L	L	L	L	U	L	U	L	L	L	U	L	L	U	H	L	15	1	4
Cornu 2012[54]	#1969	L	L	L	L	U	L	L	L	U	L	L	U	H	L	U	U	H	U	L	L	12	2	6
Damon 2014[34]	#1373	L	L	L	L	L	L	L	L	L	L	L	U	H	L	U	L	L	L	L	L	17	1	2
Dias 2014[87]	#4991	L	L	L	L	U	L	L	L	U	L	L	U	H	L	L	L	H	U	L	L	14	2	4
Dmochowski 2008[117]	#10812	L	L	L	L	L	L	L	H	U	L	L	L	L	L	L	L	U	U	H	L	15	2	3
Drake 2015[101]	#15546	L	L	L	L	L	L	L	L	U	U	L	U	H	L	U	L	H	U	L	L	13	2	5
DuBeau 2014[25]	#15558	L	L	L	L	L	L	L	L	U	L	L	L	U	L	U	L	L	U	L	L	16	0	4
Duckett 2006[98]	#4217	L	L	L	L	L	L	H	L	U	L	L	U	H	L	U	L	H	U	L	L	13	3	4
Faaborg 2009[37]	#13213	L	L	L	L	U	L	L	U	U	U	L	U	H	L	U	H	H	U	U	L	9	3	8
Filocamo 2007[81]	#11513	L	L	L	L	U	L	L	L	U	L	L	L	H	L	H	L	U	U	L	L	14	2	4

Study name	Endnote	Questions																				No. "L"	No. "H"	No. "U"
		1	2	3	4	5	6	8	9	11	12	13	14	15	16	19	20	23	25	26	28			
Freeman 2011[72]	#2539	L	L	L	U	U	L	L	L	U	L	L	U	H	L	U	U	L	U	L	L	12	1	7
Ghoniem 2009[78]	#3326	L	L	L	L	L	L	L	L	U	L	L	L	H	L	L	L	U	U	L	L	16	1	3
Glazener 2014[41]	#13156	L	L	L	L	L	L	L	L	L	H	L	U	H	L	L	H	L	U	H	L	14	4	2
Glazener 2011[99]	#847	L	L	L	L	L	L	L	L	L	L	L	U	H	L	L	L	L	L	L	L	18	1	1
Graf 2011[29]	#10132	L	L	L	L	L	L	L	L	U	L	L	L	U	L	L	L	L	U	L	L	17	0	3
Groen 2011[95]	#2358	L	L	L	L	U	L	L	L	U	L	L	U	H	L	U	L	H	U	L	L	13	2	5
Groutz 2011[70]	#13090	L	L	L	L	L	L	H	L	L	L	L	U	H	L	L	L	H	U	L	L	15	3	2
Guerrero 2010[57]	#2702	L	L	L	L	L	L	H	L	U	L	L	U	H	L	U	U	L	U	L	L	13	2	5
Haab 2006[90]	#11577	L	L	L	L	L	L	L	H	U	H	U	U	H	L	U	L	H	U	H	L	10	5	5
Harms 2007[64]	#3914	L	L	L	L	L	L	L	L	U	H	L	U	H	L	U	L	H	U	L	L	13	3	4
Herschorn 2010[105]	#11509	L	L	L	L	L	L	L	L	U	L	U	L	U	H	U	L	L	U	H	L	13	2	5
Herschorn 2013[108]	#15552	L	L	U	L	L	L	L	L	U	L	L	L	L	L	U	L	U	U	H	L	15	1	5
Houwert 2010[58]	#2950	L	L	L	L	L	L	L	L	U	L	L	U	H	L	U	U	H	L	L	L	14	2	4
Janssen Korea 2013[111]	#14046	L	L	U	L	H	L	L	L	U	H	L	H	H	L	U	L	U	U	H	L	10	5	5
Kaplan 2011[118]	#11473	L	L	L	L	L	H	L	L	U	L	L	L	L	L	U	L	U	U	H	L	14	2	4
Karram 2009[114]	#10655	L	L	U	L	H	L	L	H	U	U	L	L	L	H	U	L	U	U	L	L	11	3	6
Kondo 2007[82]	#3822	L	L	L	L	L	L	H	L	U	L	L	U	H	L	U	H	H	U	L	L	12	4	4
Krofta 2010b[59]	#2953	L	L	L	L	L	L	L	L	U	L	L	U	H	L	U	L	L	U	L	L	15	1	4
Labrie 2013[71]	#13133	L	L	L	L	L	L	L	L	L	L	L	U	H	H	L	L	L	U	L	L	16	2	2
Lacima 2010[30]	#2779	L	L	L	L	U	L	H	L	U	L	L	U	H	L	U	U	H	U	L	L	11	6	3
Lanoe 2009[77]	#10419	L	L	L	L	L	L	L	L	L	L	L	U	H	L	U	L	H	L	L	L	16	2	2
Lee 2013[75]	#1656	L	L	L	L	L	L	L	L	U	H	L	U	H	L	L	L	H	U	H	L	13	4	3
Lee 2015[65]	#5274	L	L	L	L	L	L	L	L	U	L	L	U	H	L	L	L	L	L	L	L	17	1	2
Leroi 2012[16]	#9773	L	L	L	L	L	L	L	L	L	L	L	L	L	L	L	L	U	U	L	L	18	0	2
Liao 2013[26]	#15559	L	L	L	L	L	L	L	L	U	L	L	H	H	L	U	L	H	U	L	L	14	3	3
Liebergall 2013[61]	#3257	L	L	L	L	L	L	H	L	U	L	L	U	H	L	U	H	H	U	L	L	12	4	4

Study name	Endnote	Questions																				No. "L"	No. "H"	No. "U"
		1	2	3	4	5	6	8	9	11	12	13	14	15	16	19	20	23	25	26	28			
Manassero 2007[43]	#11477	L	L	L	L	L	L	H	L	L	L	L	U	L	L	L	L	U	L	L	L	17	1	2
Marchiori 2010[89]	#11411	L	L	L	L	L	U	H	L	L	L	L	U	H	L	H	L	U	U	L	L	13	4	3
Masata 2012[68]	#9798	L	L	U	L	L	L	L	L	H	L	L	U	H	L	L	L	L	U	L	L	15	2	3
Moore 2008[42]	#11474	L	L	H	L	U	L	L	H	U	H	L	U	L	L	L	L	L	L	H	L	13	4	3
Mostafa 2013[49]	#1538	L	L	L	L	U	L	U	L	L	L	L	U	H	L	U	L	L	U	L	L	14	1	5
Natale 2014[85]	#4664	L	L	L	L	L	L	L	L	U	L	L	U	H	L	L	L	H	U	L	L	15	3	2
NCT00090584 2013[94]	#13577	L	L	L	L	U	L	U	L	L	L	U	U	H	L	U	L	L	U	L	L	13	1	6
Neuman 2011[55]	#2258	L	L	L	H	L	L	L	L	U	U	L	U	H	L	U	U	H	H	L	L	11	4	5
NCT01272284 2011[74]	# 14621	L	L	L	L	U	L	L	L	U	L	L	U	H	L	U	L	U	U	L	L	13	1	6
Nitti 2013[103]	#9683	L	L	L	L	L	L	L	L	U	L	L	L	L	L	L	L	U	U	L	L	17	0	3
North 2010[60]	#2955	L	L	L	U	H	L	H	L	U	L	L	U	H	L	U	L	H	U	H	L	10	5	5
Palomba 2014[86]	#4757	L	L	L	L	L	L	L	L	L	L	L	U	L	L	L	L	H	U	L	L	17	1	2
Paquette 2014[28]	#6392	L	H	L	L	U	L	L	U	L	U	U	U	U	L	L	L	H	U	U	U	9	2	9
Primus 2006[83]	#4000	L	L	L	L	L	L	L	L	U	L	L	U	H	L	U	L	H	U	L	L	14	2	4
Rehder 2012[79]	#2053	L	L	L	L	U	L	L	L	U	L	L	U	H	L	U	U	H	U	L	L	12	2	6
Rogers 2009[96]	#11496	L	L	L	L	U	L	L	L	U	L	L	U	U	L	L	L	H	U	L	L	14	1	5
Rovner 2011[104]	#11452	L	L	L	L	L	L	L	L	U	L	L	L	L	H	U	L	U	U	L	L	15	1	4
Sand 2012[112]	#6213	L	L	U	L	H	H	H	U	U	U	U	L	U	L	U	L	U	U	U	L	7	3	10
Schagen van Leeuwen 2008[88]	#11523	L	L	L	L	L	L	L	L	U	L	L	L	L	L	L	L	U	U	L	L	17	0	3
Schweitzer 2015[47]	#1199	L	L	L	U	U	L	L	U	L	U	L	L	H	L	U	L	L	U	H	L	12	2	6
Serati 2013[50]	#1571	L	L	L	L	U	L	U	L	L	L	L	U	H	L	U	L	L	U	L	L	14	1	5
Serra 2013[67]	#9689	L	L	L	L	L	L	L	L	L	L	L	U	H	L	L	L	H	U	L	L	16	2	2
Siegel 2015[93]	#4552	L	L	L	U	L	L	L	L	U	U	L	U	H	L	U	L	U	H	L	L	12	2	6
Sjostrom 2015[73]	#15548	L	L	L	L	L	L	L	L	U	H	L	U	H	L	U	L	L	U	H	L	13	3	4
Staskin 2006[40]	#11558	L	L	H	H	H	L	L	H	U	U	U	U	U	H	H	L	H	U	U	L	6	7	7

Study name	Endnote	Questions																				No. "L"	No. "H"	No. "U"
		1	2	3	4	5	6	8	9	11	12	13	14	15	16	19	20	23	25	26	28			
Staskin 2007[119]	#15553	L	L	U	L	U	L	L	L	U	L	L	L	L	L	U	L	L	U	H	L	14	1	5
Staskin 2011[113]	#10091	L	L	L	L	L	L	L	L	U	H	L	L	L	H	U	L	U	U	H	L	13	3	4
Surwitt 2009[97]	#10558	L	L	U	L	H	L	L	L	U	L	L	U	H	L	H	L	H	U	L	L	12	4	4
Sussman 2013[39]	#11682	L	L	L	U	L	L	H	L	U	U	L	L	L	L	U	L	U	U	L	L	15	1	6
Sze 2009[31]	#3161	L	L	L	L	H	L	H	H	U	L	L	U	H	L	U	U	H	U	H	L	9	5	6
Thomas 2014[38]	#15557	L	L	U	L	L	L	H	U	U	U	U	U	H	L	U	L	U	L	H	L	9	3	8
Tijandra 2008[33]	#11675	L	L	L	L	L	H	L	U	U	U	L	U	H	L	U	L	U	U	U	L	10	2	8
Tincello 2012[91]	#11438	L	L	L	L	L	L	L	H	U	H	L	L	L	L	L	L	L	U	H	L	15	3	2
Tommaselli 2013b[52]	#1728	L	L	L	U	U	L	L	L	L	U	L	L	L	L	U	L	U	U	H	L	13	2	5
Tommaselli 2013[51]	#1710	L	L	L	L	L	L	L	L	U	L	L	U	L	L	U	L	L	H	L	L	16	1	3
Uludag 2011[35]	#2304	L	L	H	H	U	L	H	H	U	L	L	U	H	L	U	L	H	U	H	L	8	7	5
Van Leijsen 2013[66]	#9658	L	L	L	U	U	L	H	L	L	H	L	U	L	L	L	L	L	U	H	L	13	3	4
Viktrup 2007[76]	#10965	L	L	H	L	H	L	L	L	U	U	L	U	U	H	U	L	H	U	L	L	10	4	6
Visco 2012[102]	#15547	L	L	L	L	L	L	L	L	U	L	L	L	L	L	U	L	L	L	L	L	18	0	2
Wagg 2014[23]	# 15549	L	L	L	L	U	U	L	L	U	U	L	H	H	L	U	L	U	U	H	L	10	3	7
Ward 2008[63]	#3630	L	L	H	H	H	L	L	L	U	L	L	U	H	L	U	L	L	U	L	L	12	4	4
Wexner 2010[27]	#2918	L	L	L	L	L	L	L	H	U	H	L	U	H	L	L	L	H	U	H	L	12	5	3
Williams 2005[44]	#11505	L	L	L	U	L	L	H	L	L	L	L	U	L	L	H	L	L	U	L	L	15	2	3
Williams 2006[84]	#4019	L	L	L	L	L	L	L	L	L	L	L	U	H	L	L	H	L	U	L	L	16	2	2
Wyndaele 2009[115]	#10579	L	L	L	L	L	L	L	L	U	L	L	H	H	H	U	L	H	U	L	L	13	4	3
Yamaguchi 2007[116]	#11732	L	L	L	L	L	L	L	L	U	L	L	L	L	L	U	L	U	U	L	L	16	0	4
Zuckerman 2014[48]	#1334	L	L	L	L	L	L	U	L	L	L	L	U	H	L	U	L	L	U	L	L	15	1	4

H=High Risk; L=Low Risk; U=Unclear Risk; No=Number

Table S3: Study characteristics for female and male patients with SUI

Study name	Endnote	N	Inclusion criteria	Mean/ Median age (yrs)	% of males	RoB No. Low *
**WOMEN**						
Carey 2006[69]	#11686	167	Women with USI; failed conservative therapy	52.3/ 51.0	0	19
Palomba 2014[86]	#4757	209	Diagnosis of SUI/MUI confirmed by urodynamic assessment; incontinent after conservative management, in MUI -persistent, clinically significant SUI under oral antimuscarinic therapy	64.1 /63.8	0	17
Lee 2015[65]	#5274	206	SUI or urodynamic SUI	51.6	0	17
Schagen van Leeuwen 2008[88]	#11523	126	Community-dwelling women of ≥65 years with symptoms of SUI or S-MUI for≥3 consecutive months and ≥7 incontinence episodes per week as determined by the stress/urge incontinence questionnaire S/UIQ were eligible. For patients in the S-MUI group, ≥50% of incontinence episodes had to be due to stress.	70.6	0	17
Ghoniem 2009[78]	#3326	247	SUI primarily due to ISD; no improvement with conservative treatments; viable mucosal lining and normal bladder capacity. Extension: p.1448: Reported success (Stamey grade improvement) from baseline; Primary diagnosis of SUI, primarily due to ISD; <90 cm H2O leak point pressure; >25 cm H2O detrusor pressure;SUI not improved by prior conservative therapy or surgery;Viable urethral mucosal lining; Normal bladder capacity	60.7 /61.3	0	16
Williams 2006[84]	#4019	238	Women with a urodynamic diagnosis of USI or mixed UI and DO who had already had an 8-week primary-care intervention.	56.7	0	16
Labrie 2013[71]	#13133	174	Age 35-80; SUI moderate or severe according to Sandvik index; in case of MUI SUI predominant (more SUI than UUI episodes); no previous treatment or physiotherapy > 6 months before randomization.	50.0	0	16
Tommaselli 2013[51]	#1710	55/ 55	SUI as diagnosed by clinical evaluation and urodynamics, age > 30 years, and previously failed pelvic floor muscle training.	59.6/ 62.5	0	16
Krofta 2010b[59]	#2953	149/ 151	All women who had urodynamically proven primary SUI including a positive stress test were candidates for inclusion. Surgery was only offered if conservative therapy was unsuccessful.	57.2/57.8	0	15
Masata 2012[68]	#9798	197	Age >18 years; urodynamic SUI; agreement with postoperative follow-up;	56.5	0	15
Natale 2014[85]	#4664	92	Females with primary stress urinary incontinence (SUI) or mixed urinary incontinence with predominant SUI, diagnosed using both the cough stress test and urodynamics. All had unsuccessfully undergone prior pelvic floor training	58	0	15

Study name	Endnote	N	Inclusion criteria	Mean/ Median age (yrs)	% of males	RoB No. Low *
Groutz 2011[70]	#13090	61	Women who underwent TVT-O during the year 2005 for urodynamically confirmed overt SUI or MUI with predominant SUI; failed conservative treatment	56.6	0	15
Houwert 2009b[58]	#2950	437	All women had symptoms of predominant SUI. Three hundred forty-three women (79%) had symptoms of pure SUI and 94 women (21%) had symptoms of mixed urinary incontinence (MUI). In women with MUI, anticholinergic medical treatment was given before surgical treatment was considered and did not alleviate their symptoms.	51.1	0	14
Serati 2013[50]	#1571	196	Women who complained of pure SUI symptoms without any other urinary disorder	55 (Med)	NR	14
Mostafa 2013[49]	#1538	137	All women admitted for surgical treatment of urodynamic SUI as a sole procedure were invited to participate in this study. All women have failed or declined pelvic floor muscle training.	51	0	14
Primus 2006[83]	#4000	103	Women who presented with urinary incontinence symptoms, including genuine, recurrent or mixed stress. All patients had unsuccessfully attempted treating their symptoms using pelvic floor exercises. Patients who indicated that these symptoms represented a significant negative impact on their life and who expressed a strong desire to have the operation were included.	60.2	0	14
Dias 2014[87]	#4991	50	Age >18 years; a history of clinically pure SUI or MUi with predominantly bothersome SUI symptoms; a positive cough stress test (CST) with clinical hypermobility of the urethra; previous failed or denied conservative therapy	53.2	0	14
Van Leijsen 2013[66]	#9658	508	Women with uncomplicated SUI (pure SUi or MUI with predominat SUI); failed conservative therapy; candidates for surgery; SUI on physical examination or indicated on bladder diary	55/54/52 /51	0	13
Tommaselli 2013[52]	#1728	128/ 120	SUI as diagnosed by clinical evaluation and urodynamics; age >30 year; previously failed pelvic floor muscle training	60.4/ 56.9	0	13
Guerrero 2010[57]	#2702	72/ 50/ 79	The women needed to be over 18 years of age with clinically and urodynamically diagnosed SUI.	54.3/52.4 /52.1	0	13
Harms 2007[64]	#3914	191	All women suffered from objectively verified stress urinary incontinence.	59 (Med)	0	13
Barry 2008[63]	#3620	107/ 80	Failed conservative management for symptomatic stress incontinence or required prophylactic incontinence surgery during prolapse repair for occult stress incontinence (no pre-operative subjective complaint of urinary stress leakage but found to have USI).	53.6/54.2	0	13
Sjostrom	#15548	155	Women; 18–70 years; SUI ≥ one time/week; Ability to read and write Swedish; Access to	47.9/49.4	0	13

Study name	Endnote	N	Inclusion criteria	Mean/ Median age (yrs)	% of males	RoB No. Low *
2015[73]			computer with Internet connection.			
NN. NCT0127228 4 2011[74]	# 14621	112	Female at least 18 years of age; able and willing to complete all procedures and follow-up visits indicated in the protocol; confirmed SUI through cough stress test or urodynamics; failed two non-invasive incontinence therapies (such as Kegel exercise, behaviour modification, pad use, biofeedback, etc) for >6 month.	54.5	0	13
Lee 2013[75]	#1656	56	Women with symptoms of SUI and more than 2 g of urine leakage on a standard pad test with a full bladder	52.2	0	13
Duckett 2006[98]	#4217	51	Women, who had symptoms of both stress and urge incontinence and were proven to have idiopathic DO and USI on urodynamics, undergo regular review.	52 (Med)	0	13
Ward 2008[63]	#3630	344	Women with urodynamic stress incontinence and who had failed to respond to pelvic floor muscle exercise.	NR	0	12
Freeman 2011[72]	#2539	93/ 100	Women over 21 years of age with USI, or mixed urinary incontinence where SUI was the predominant symptom, failed PFMT, and willing and able to complete a 4-day urinary diary.	50/54 (Med)	0	12
Schweitzer 2015[47]	#1199	156	Women; aged between 35 and 80 years; who had moderate to severe SUI, defined as a Sandvik score 3 or greater, and who had not experienced relief after pelvic floor muscle physiotherapy treatment.	49.6	NR	12
Liebergall-Wischnitzer 2013[61]	#3257	64/ 79	Women aged 20–65 with a history compatible with SUI and at least 1 g urinary leakage in a 1-hour clinic-based pad test and with the ability to understand instructions in Hebrew or English.	48.5/47.8	0	12
Cornu 2012[54]	#1969	95	Presence of SUI at clinical examination; clinical hypermobility of the bladder neck; previous failed conservative therapy for SUI (pelvic floor muscle training).	56	0	12
Kondo 2007[82]	#3822	79	In the present investigation a long-term assessment was performed through a questionnaire that was dispatched to 112 women who had been evaluated at the second, medium-term assessment.	53.2	0	12
Neuman 2011[55]	#2258	80/ 82	Diagnosis of SUI based on the patient's personal history and a positive cough test with the bladder holding 300 to 400 mL.	54/53	0	11
Viktrup 2007[76]	#10965	958	Women 18 years of age and older were enrolled in the studies, but the phase 2 study had an upper age limit of 65 years of age. To be enrolled, patients must have reported a predominant symptom of SUI with a weekly IEF of 4 in the phase 2 study and of 7 or greater in the phase 3	NR	0	10

Study name	Endnote	N	Inclusion criteria	Mean/ Median age (yrs)	% of males	RoB No. Low *
			studies.			
North 2010[60]	#2955	60	Female patient aged 18 years or over; Urodynamic stress incontinence on multichannel urodynamic; No desire for future pregnancy; Able and willing to give informed consent; All women had found initial treatment with pelvic floor muscle training ineffective and were considering surgical treatment with TVT	50 (Med)	0	10
Abdel-fattah 2012[53]	#1904	341	Women with urodynamic SUI who had previously failed or declined pelvic floor muscle treatment. Women with urodynamic mixed UI, however, with a predominant SUI complaint, were included.	NR	0	9
**MEN**						
Lanoe 2009[77]	#10419	84	Men treated with the InVance sub-urethral sling	68	84%	16
Serra 2013[67]	#9689	61	SUI; unsuccessful treatment with pelvic floor rehabilitation and pharmacologic therapy (duloxetine); undergone radical prostatectomy (established stress urinary incontinence) >1 year previously	65 (Med)	100%	16
Zuckerman 2014[48]	#1334	102	Preoperative patient evaluation was consistent with what we have previously reported, including documented stress urinary incontinence (SUI), a bladder with adequate capacity and compliance on urodynamic testing, and adequate sphincter contraction visualized cystoscopically.	66.1	100%	15
Filocamo 2007[81]	#11513	102	Predominant symptoms of postprostatectomy SUI; at least four stress incontinent episodes daily; a positive 1-h pad test	64.6/ 65.7	100%	14
Marchiori 2010[89]	#11411	332	Patients after radical prostatectomy; Moderate to severe incontinence at 30 days after catheter removal	67/66.5	100%	13
Bochove-Overgaauw 2011[56]	#2490	100	SUI as a result of open or laparoscopic radical prostatectomy, transurethral resection of the prostate or external radiotherapy for prostate cancer. For patients to be eligible for inclusion in this study SUI had to persist after more than 6 months of pelvic floor muscle training.	66	100%	13
Rehder 2012[79]	#2053	156	SUI following RP, transurethral resection of the prostate (TURP), or open adenectomy; who were stress incontinent for >1 yr after surgery; all patients had undergone failed conservative treatment (all patients had PFMT, and some in addition electrical stimulation therapy); and required more than one incontinence pad per day.	68 (Med)	100%	12
Bauer 2009[80]	#2979	124	Men with mild to severe SUI after RP were included in this study.	68.9	100%	12

*) Number of low risk items in the Risk of Bias assessment (out of 20 items in total – See Table 5)

Table S4: Treatment characteristics in studies for female and male patients with SUI

Study name	Endnote	Treatment	Further details of intervention	Duration of treatment
**WOMEN**				
**Women-Tapes**				
Guerrero 2010	#2702	TVT	The TVT-TM (Gynecare) tapes were inserted using the standard technique described by Ulmsten.	Surgery (35min)
Krofta 2010b	#2953	TVT	TVT technique described by Ulmsten et al. Specifically, the procedure was performed under local anaesthesia supplemented by intravenous analgosedation. Cystoscopy was routinely performed, and a cough test was performed with the patient coughing repeatedly with a bladder volume of 300 mL.	Surgery (33min)
Barry 2008	#3620	TVT	The TVT procedures were performed as described by Ulmsten except that the choice of anaesthesia was left to the surgeon.	Surgery (19min)
Harms 2007	#3914	TVT	TVT insertion (Gynecare TVT device; Gynecare, Ethicon, Norderstedt, Germany) under local anaesthesia, according to Ulmsten and Petros.	Surgery (NR)
Duckett 2006	#4217	TVT	The TVT procedure was performed as described by Ulmsten et al. and spinal anaesthesia was used.	Surgery (NR)
Ward 2008	#3630	TVT	NR	Surgery (NR)
Mostafa 2013	#1538	TVT-O	SMUS-tension-free vaginal tape-obturator (TVT-O)	Surgery (35 min)
Abdel-fattah 2012	#1904	TVT-O	Performed using TVT-O (Ethicon Inc., Somerville, NJ, USA) for the inside-out	NR
Serati 2013	#1571	TVT-O	Tension Free Vaginal Tape (Obturator) (TVT-O) procedure (Gynecare; Ethicon Inc., Somerville, NJ, USA). All of the TVT-O procedures were performed by two experienced surgeons	NR
Tommaselli 2013	#1728	TVT-O	No further info	Single procedure
Masata 2012	#9798	TVT-O	(Ethicon Women's Health, Sommerville, NJ, USA); according to original technique	Single procedure
Groutz 2011	#13090	TVT-O	GYNECARE TVT Obturator System, Somerville, NJ;	Single procedure

Study name	Endnote	Treatment	Further details of intervention	Duration of treatment
Tommaselli 2013	#1710	TVT-O	Established midurethral sling procedure using the TVT-O device (Ethicon Gynecare, Somerville, NJ, USA) originally described by de Leval.	Surgery (8min)
Neuman 2011	#2258	Tension-free vaginal tape–obturator (TVT-O)	The TVT surgical needle was inserted using the technique described by de-Leval for TVT-O, with the hammock method.	Surgery (17min)
Houwert 2010	#2950	TOT TVT-O	TVT-O (Gynecare), an "insideout" procedure, was performed as described by de Leval.	Surgery (16min)
Krofta 2010	#2953	TVT-O	The TVT-O procedure was performed according to the original technique described by de Leval. For the current study, the procedure was performed under spinal or local anaesthesia supplemented by intravenous analgosedation. Hydrodissection was performed routinely only in case of local anaesthesia.	Surgery (24min)
Tommaselli 2013	#1710	Modified TVT-O	Same surgical procedure using the modified less invasive technique with the same device	Surgery (7min)
Abdel-fattah 2012	#1904	TOT-Aris	Performed using TOT-Aris (Coloplast Corp., Minneapolis, MN, USA) for the outside-in approach	NR
Freeman 2011	#2539	Monarc TOT	Surgery was performed using standard techniques for both procedures, as agreed by all investigators. Patients had a choice of local, regional or general anaesthetic. Antibiotic and venous thromboembolism prophylaxes were provided in all cases.	Surgery (28min)
Houwert 2010	#2950	TOT MONARC	The Monarc tape was inserted through the "outside-in" route, using the technique recommended by the manufacturer.	Surgery (16min)
Barry 2008	#3620	Monarc TOT	The Monarc slings were performed as described in a previous publication, but to standardise sling tension, surgeons were requested to perform either a cough test or simulated cough (Crede manoeuvre) with 300 ml of water in bladder intra-operatively for both procedures. Catheters were not routinely placed post-operatively in patients undergoing sling surgery alone, unless intra-operative bladder injury occurred, whereby catheter would then be left in overnight.	Surgery (15min)
Schweitzer 2015	#1199	transobturator sling	No further details on the intervention	NR
Lee 2015	#5274	transobturator midurethral sling +/- POP repair	Monarc (American Medical Systems, Minnetonka, MN)	Single procedure

Study name	Endnote	Treatment	Further details of intervention	Duration of treatment
Van Leijsen 2013	#9658	Surgery	miduretheral sling	Single procedure
Van Leijsen 2013	#9658	Individually tailored treatment	midurethral sling, anticholinergics for detrusor overactivity, prolonged pelvic floor exercises or bladder training for dysfunctional voiding, a pessary, expectant management, intravesical botulinum toxin injections, pretibial nerve stimulation	various
Van Leijsen 2013	#9658	Individual treatment according to urodynamic findings	midurethral sling, anticholinergics for detrusor overactivity, prolonged pelvic floor exercises or bladder training for dysfunctional voiding, a pessary, expectant management, intravesical botulinum toxin injections, pretibial nerve stimulation	various
North 2010	#2955	Single incision sling procedure (Minitape-TM)	Mid-urethral tape sling which is inserted through a single vaginal incision with no abdominal or groin exit incisions. The tape is a monocomponent, polypropylene mesh, which is 200 lm thick and 14 cm long. The needle introducer is approximately half the diameter of the TVT needle introducer (2.5 mm in diameter). A terminal anchor on each end of the tape fixes it in the retropubic space.	Surgery (24min)
Primus 2006	#4000	SPARC sling system	The SPARC Female Sling System is a minimally invasive procedure designed to correct stress urinary incontinence in women. This system uses a suprapubic surgical approach to position the sling under the midurethra, employing two thin, curved stainless steel needles advanced via two small incisions above the pubic bone to a vaginal incision below the urethra. Only one cystoscopy is required, after both needles are positioned, to confirm the integrity of the urethra and bladder.	Surgery (42min)
Palomba 2014	#4757	Retropubic TVT	SPARC system (Tegea for AMS)	Single procedure
Freeman 2011	#2539	Retropubic TVT	Surgery was performed using standard techniques for both procedures, as agreed by all investigators. Patients had a choice of local, regional or general anaesthetic. Antibiotic and venous thromboembolism prophylaxes were provided in all cases.	Surgery (30min)
Houwert 2010	#2950	Retropubic TVT	The TVT (Gynecare, Ethicon Inc, Sommerville, NJ) procedure was performed as described by Ulmsten et al.	Surgery (NR)
**Women-Sling**				
Mostafa 2013	#1538	SIMS	adjustable anchored single incision mini-sling (SIMS-Ajust - C. R. Bard, Inc.)	Surgery (35 min)
Schweitzer 2015	#1199	adjustable single-incision sling	No further details on the intervention	NR

Study name	Endnote	Treatment	Further details of intervention	Duration of treatment
NCT01272284 2011	# 14621	Altis® Single Incision Sling	Coloplast Altis single incision sling system	NR
Natale 2014	#4664	Single-Incision-Sling	Single Incision Sling (AjustTM C.R. Bard Inc., New Providence, NJ, USA); surgical technique described on p. 49	Single procedure
Palomba 2014	#4757	single-incision mini-slings	Ajust (Bard SpA, Rome, Italy), MiniArc (Tegea for AMS, Bologna, Italy), or TVT Secur System (Johnson & Johnson, Rome, Italy)	Single procedure
Dias 2014	#4991	single-incision sling (SIS)	Altis® (Coloplast); full description of surgical techique at p.1091	Single procedure
Lee 2015	#5274	slingle incision sling +/-POP repair	MiniArc (American Medical Systems, Minnetonka, MN)	Single procedure
Cornu 2012	#1969	Ajust-TM single incision transobturator sling procedure	All patients were implanted with the AjustTM adjustable single incision sling (Bard Urological Division, Covington, GA, USA). All procedures were performed or supervised by one experienced surgeon. No associated procedures were performed, except for 1 patient presenting SUI associated with dysuria owing to previous transobturator sling misplacement. No prolapse surgery was conducted at the time of sling implantation.	Surgery (15min)
Guerrero 2010	#2702	Autologous fascial sling	The 'sling-on-a-string' technique as this had been shown to be equivalent to the standard longer sling with lower morbidity.	Surgery (54min)
**Women-Colposuspension**				
Carey 2006	#11686	Burch Colposuspension	Open, description of surgical procedure	Single procedure
Carey 2006	#11686	Burch Colposuspension	Laparoscopic, description of surgical procedure	Single procedure
Ward 2008	#3630	Burch Colposuspension	NR	Surgery (NR)
**Women-PFMT (Supervised)**				
Labrie 2013	#13133	Physiotherapy	PFMT according to the Dutch guidelines; A supervised program to build up to 8 to 12 maximal contractions three times per day; 1-2 wk interval, 9 sessions in 9-18 wks; biofeedback, electrostimulation	up to 4.2 mo

Study name	Endnote	Treatment	Further details of intervention	Duration of treatment
Liebergall-Wischnitzer 2013	#3257	Pelvic floor muscle training (PFMT)	Pelvic floor muscle training exercises the pelvic floor muscles, including levator ani, which acts as the main support for the bladder neck during periods of increased intra-abdominal pressure. The aim of PFMT is to both strengthen and improve the timing of pelvic floor muscle contractions.	12 wks (30 min, 6 sessions)
Kondo 2007	#3822	Pelvic floor muscle training (PFMT)	Essentially, the training comprised repeated muscle contractions of the pelvic floor and the rapid locking of the perineum just before the abdominal strain. In the training session experienced nurses and doctors supervised the exercises, and they taught how continence was maintained or lost and the treatment rationale was also explained. At home women were instructed to practice the exercise 30 times a day.	8 weeks (60–90 min once a week)
Williams 2006	#4019	Intensive PFMT	Training in PFMT was provided by specially trained nurses, after an initial digital assessment and perineometry. Women were taught how to do correct PF contractions and an individualized exercise regimen was given.	3m
Lee 2013	#1656	Extracorporeal biofeedback combined with pelvic floor muscle training (PFMT)	All participants visited the same physiotherapist twice a week for the first 4 weeks and then once a week for the next 8 weeks. The Hue & Joy extracorporeal biofeedback device (HnJ-5000; Furon Medical, Korea) was used in the study at an outpatient clinic. The Hue & Joy extracorporeal biofeedback device (HnJ-5000; Furon Medical, Korea) was used in the study at an outpatient clinic. Participants were advised to perform 30 sessions of PFMT (100 contractions) at home daily	12 wks
Liebergall-Wischnitzer 2013	#3257	Paula method (circular muscle strengthening)	The Paula method is a circular muscle exercise protocol that works on the premise that all sphincters in the body are synchronized, so that movement of one sphincter affects distant circular muscles.	12 wks (12 45min weekly sessions)
**Women-PFMT (Unsupervised)**				
Sjostrom 2015	#15548	Internet based PFMT	PFMT >=8 contractions three times per day; information on SUI, lifestyle advice and training reports. Asynchronous, individually tailored e-mail support from urotherapist during the treatment period. 8 escalating levels, and also included cognitive behavioural therapy assignments for lifestyle changes (if applicable) and for the identification and changes in behaviours of avoidance and/or redundant security measures (if applicable).	3m
Sjostrom 2015	#15548	Postal based PFMT	PFMT >=8 contractions three times per day; information on SUI, lifestyle advice and training reports. Participants trained on their own.	3m

Study name	Endnote	Treatment	Further details of intervention	Duration of treatment
**Women-Vaginal cone therapy**				
Williams 2006	#4019	Vaginal cone therapy	At the first clinical visit after randomization women were instructed how to insert the cones, supervised by the nurse; during this consultation the appropriate weight of cone for starting treatment was identified (Femina, Urohealth Systems Inc., Newport Beach, CA, USA).	3m
**Women-Lifestyle Advice (Unsupervised)**				
Williams 2006	#4019	Continued primary behaviour intervention	Advice on fluid intake, caffeine intake, bladder re-education, PF awareness (PFA) and weight loss. This was continuation of an 8-week period for all three arms.	3m
**Women-Bulking agents**				
Ghoniem 2009	#3326	Bulking agent	Macroplastique	Once or twice
Ghoniem 2009	#3326	Bulking agent	Contigen	Once or twice
**Women- Duloxetine**				
Viktrup 2007	#10965	Duloxetine	40mg bid	12 wks
Schagen van Leeuwen 2008	#11523	Duloxetine	Oral 20mg bid for 2 weeks and 40mg bid for 10 weeks	12 wks
**MEN**				
**Male Sling**				
Zuckerman 2014	#1334	AdVance transobturator male slings	The sling was fixed to the corpus spongiosum in each of the 4 corners, with the proximal aspect of the sling fixed to the previous site of attachment of the central tendon.	NR
Serra 2013	#9689	male sling	AdVance/AdVance XP	Single procedure
Bauer 2009	#2979	AdVance Sling	The AdVance sling is a polypropylene monofilament mesh that is selfanchoring due to its woven nature. It is implanted using a transobturator approach via a median perineal incision utilizing a trocar system. The procedure is performed as previously described with some minor changes. The middle part of the sling is fixed with long-term resorbable suture distally on the bulbus such that the bulbus appendix can rotate retrourethrally and cranially to avoid hypermobility.	Surgery (NR)

Study name	Endnote	Treatment	Further details of intervention	Duration of treatment
Rehder 2012	#2053	Transobturator Retroluminal Repositioning Sling Suspension (AdVance)	All patients underwent implantation of the AdVance male sling under general or locoregional anaesthesia. The transobturator sling was implanted using an operative technique previously described.	Surgery (NR)
Bochove-Overgaauw 2011	#2490	Argus Sling	The operation was performed as described by Romano et al with some minor modifications	Surgery (NR)
Lanoe 2009	#10419	InVance sub-urethral sling	Bone anchored sub- urethral sling was implanted surgically. They were hospitalised for average 3 days.	Surgery (69 mins)
**Men-PFMT (Supervised)**				
Filocamo 2007	#11513	PFMT	Verbal instructions and manual feedback about pelvic floor muscle contraction, written instructions to perform three sets of 10 contractions lasting 5 s with 10 s of muscular relaxation daily; visits every 15 days	3.7m
Marchiori 2010	#11411	PFMT guided+ biofeedback+ electrical stumulation	Instruction how to correctly continue Kegel exercises at home and intensive guided rehabilitation program - supervised and guided rehabilitation with biofeedback and electrical stimulation: 1 st session instructions an biofeedback, 2nd - 10 sets of pelvic floor electrical stimulation (PFES) lasting 15 minutes each; rehabilitation program daily for BF and instructions and a mean period of 2-3 weeks for PFES.	12m
**Men- PFMT (Unsupervised)**				
Marchiori 2010	#11411	PFMT	Kegel exercises at home by themselves after having received oral and written information from a urologist and a urodynamic advisor	12m
**Men-PFMT (Supervised) + duloxetine**				
Filocamo 2007	#11513	PFMT+duloxetine	Verbal instructions and manual feedback about pelvic floor muscle contraction, written instructions to perform three sets of 10 contractions lasting 5 s with 10 s of muscular relaxation daily; visits every 15 days; duloxetine 40 mg twice daily	3.7m

Table S5: Results of interventions for patients with SUI

Study name	Endnote	Intervention	N	Follow-up period	Type of outcome	Outcome description	With or without containment products	% of patients with outcome
**Women**								
**Women-TVT**								
Guerrero 2010	#2702	TVT	72	6m	Cure	Completely dry	NR	50%
Guerrero 2010	#2702	TVT	72	12m	Cure	Completely dry	NR	53%
Guerrero 2010	#2702	TVT	72	6m	Improvement	Either completely dry or who reported that their urinary incontinence was improved at 6 weeks	NR	90%
Guerrero 2010	#2702	TVT	72	12m	Improvement	Either completely dry or who reported that their urinary incontinence was improved at 6 weeks	NR	89%
Krofta 2010b	#2953	TVT	141	12m	Objective cure	A negative cough stress test with 300 mL of saline solution in the bladder during a multichannel urodynamic examination and a 1-h pad test weight of less than 1 g.	With	90.1%
Krofta 2010b	#2953	TVT	141	12m	Objective improvement	A negative cough stress test and a 1-h pad test weight of less than 5 g.	With	8.5%
Krofta 2010b	#2953	TVT	141	12m	Subjective cure	No leakage of urine after surgery (tick-box “never” was checked after surgery) in the ICIQ-UI SF questionnaire (scored item 1: assessment of frequency /0–5/).	Without	78.7%
Krofta 2010b	#2953	TVT	141	12m	Subjective improvement	If assessment of frequency of urine leakage after the surgery was lower than before in the ICIQ-UI SF questionnaire (scored item 1: assessment of frequency /0–5/).	Without	19.1%
Barry 2008	#3620	TVT	82	3m	Objective Cure	NR	NR	78%
Barry 2008	#3620	TVT	82	3m	Subjective Cure	NR	NR	86.6%
Harms 2007	#3914	TVT	171	36m	Cure (objective and	Dry, symptom-free patient without objective urine loss during vigorous coughing and other	NR	89.5%

Study name	Endnote	Intervention	N	Follow -up period	Type of outcome	Outcome description	With or without containment products	% of patients with outcome
					subjective)	provocative activities at a standard bladder filling of 300 ml and a demonstrable positive urethral closure pressure during stress provo-cation in the absence of detrusor instability. Additional criteria were no episodes of stress or urge incontinence in the 24-h voiding diary and no postvoid residual urine. Finally, the define-tion of cure comprised assessment of subject-tive continence by means of a self-completed detailed urinary incontinence questionnaire and the patient's history.		
Duckett 2006	#4217	TVT	46	6m	Objective cure	Normal detrusor function during filling cystometry	NR	92%
Duckett 2006	#4217	TVT	46	6m	Subjective cure	Information from interview and questionnaire	NR	91%
Ward 2008	#3630	TVT	72	5y	Cure	Negative 1-hour pad test (<1 g change in weight)	With	81%
Ward 2008	#3630	TVT	98	5y	Subjective Cure	Positive response to BFLUTS questionnaire item 7	With	63%
Mostafa 2013	#1538	TVT-O	69	4 to 6m	Success	Patient reported success rate, defined as "very much improved" or "much improved" on the PGI-I	NR	92%
Mostafa 2013	#1538	TVT-O	62	1yr	Success	Patient reported success rate, defined as "very much improved" or "much improved" on the PGI-I	NR	53/62 (85.5%)
Mostafa 2013	#1538	TVT-O	62	1yr	Objectives cure	Negative Cough Stress Test (CST)	NR	51/62 (82.3%)
Mostafa 2013	#1538	TVT-O	57	1yr	Improvement QoL	>= 18 points improvement in total King's Health Questionnaire (KHQ) score	NR	50/57 (87.7%)
Abdel-fattah 2012	#1904	TVT-O	170	3 yr	Success	Based on Patient Global Impression of Improvement response	NR	93/126 (73.8%)
Abdel-fattah 2012	#1904	TVT-O	170	1 yr	Success	Based on Patient Global Impression of Improvement response	NR	81.30%

Study name	Endnote	Intervention	N	Follow -up period	Type of outcome	Outcome description	With or without containment products	% of patients with outcome
Abdel-fattah 2012	#1904	TVT-O	170	1 yr	Improvement QoL	Based on 18-point improvement in KHQ scores as the cut-off value	NR	79.40%
Serati 2013	#1571	TVT-O	181	12 m	Global cure	Patients were defined as 'cured' if they presented a negative stress test, a score reduction of at least 80% on the UDI and a response of 'much better' or 'very much better' on the PGI-I.	NR	165/181 (91.2%)
Serati 2013	#1571	TVT-O	181	12 m	Objective cure	Negative stress test. Defined as the absence of urine leakage during a cough test performed in the lithotomic and upright positions with a full bladder (ultrasonographic measurement of at least 400 mL).	NR	171/181 (94.1%)
Serati 2013	#1571	TVT-O	181	12 m	Subjective cure	International Consultation on Incontinence Questionnaire-short form (ICIQ-SF)	NR	0
Serati 2013	#1571	TVT-O	181	12 m	Subjective cure	'Very much better' or 'much better' on PGI-I,	NR	165/181 (91.1%)
Serati 2013	#1571	TVT-O	181	12 m	Subjective cure	80% reduction in UDI score	NR	165/181 (91.1%)
Tommaselli 2013	#1728	TVT-O	62	5y	Subjective-PGI-I defined	Much or very much improved	NR	79%
Tommaselli 2013	#1728	TVT-O	62	5y	Qol-improvement in quality of life	>= 20 point increase on the total I-QOL score	NR	83.9%
Tommaselli 2013	#1728	TVT-O	66	3 yr	Objective cure	Negative Cough Stress Test (CST)	NR	86.4%
Masata 2012	#9798	TVT-O	68	2y	Subjective cure	Likert scale cured	NR	89.7%
Masata 2012	#9798	TVT-O	68	2y	Subjective improvement	Likert scale improved	NR	7.4%

Study name	Endnote	Intervention	N	Follow -up period	Type of outcome	Outcome description	With or without containment products	% of patients with outcome
Masata 2012	#9798	TVT-O	68	2y	Subjective stress negative	no stress leakage of urine after assessed by ICIQ-UI SH ("Never"/"Urine does not leak" for question 6: "When does urine leak?").	NR	85.3%
Masata 2012	#9798	TVT-O	68	2y	Subjective urgency incontinence cured	ICIQ-UISH response to "Leaks before you can get to the toilet."	NR	14.7%
Masata 2012	#9798	TVT-O	68	2y	Objective cure	Negative stress test	NR	92.6%
Groutz 2011	#13090	TVT-O	61	5y	Cure	Negative stress test, no episodes of SUI, and positive (cure) global satisfaction	NR	74%
Groutz 2011	#13090	TVT-O	61	5y	Improved	Infrequent episodes of SUI	NR	8%
Tommaselli 2013	#1710	TVT-O	52	12m	Objective cure	Absence of any leakage during the CST with a 250 ml bladder filling and the stress test during the filling phase of the cystometry	NR	92.30%
Neuman 2011	#2258	Tension-free vaginal tape–obturator (TVT-O)	69	3y	Cure	There was no leakage at all	NR	87%
Houwert 2010	#2950	TOT TVT-O	85	12m	Cure	Statement of the woman of not experiencing any loss of urine upon physical activity, coughing, or sneezing.	NR	76%
Houwert 2009	#3050	TOT TVT-O	75	12m	Cure	Statement of the woman of not experiencing any loss of urine upon physical exercise.	NR	77%
Houwert 2009	#3050	TOT TVT-O	75	12m	Improvement	NR	NR	15%
Houwert 2009	#3050	TOT TVT-O	75	2-4y	Cure	Statement of the woman of not experiencing any loss of urine upon physical exercise.	NR	72%
Houwert 2009	#3050	TOT TVT-O	75	2-4y	Improvement	NR	NR	12%
Houwert 2009	#10629	TOT TVT-O	85	14m	Cure	Statement of the woman of not experiencing any	NR	77%

Study name	Endnote	Intervention	N	Follow -up period	Type of outcome	Outcome description	With or without containment products	% of patients with outcome
						loss of urine on physical exercise.		
Krofta 2010b	#2953	TVT-O	147	12m	Objective cure	A negative cough stress test with 300 mL of saline solution in the bladder during a multichannel urodynamic examination and a 1-h pad test weight of less than 1 g.	With	88.4%
Krofta 2010b	#2953	TVT-O	147	12m	Objective improvement	A negative cough stress test and a 1-h pad test weight of less than 5 g.	With	9.5%
Krofta 2010b	#2953	TVT-O	147	12m	Subjective cure	No leakage of urine after surgery (tick-box “never” was checked after surgery) in the ICIQ-UI SF questionnaire (scored item 1: assessment of frequency /0–5/).	Without	76.2%
Krofta 2010b	#2953	TVT-O	147	12m	Subjective improvement	If assessment of frequency of urine leakage after the surgery was lower than before in the ICIQ-UI SF questionnaire (scored item 1: assessment of frequency /0–5/).	Without	21.1%
Tommaselli 2013	#1710	Modified TVT-O	45	12m	Objective cure	Absence of any leakage during the CST with a 250 ml bladder filling and the stress test during the filling phase of the cystometry	NR	88.80%
Abdel-fattah 2012	#1904	TOT-Aris	171	3 yr	Success	Based on Patient Global Impression of Improvement response	NR	81/112 (72.3%)
Abdel-fattah 2012	#1904	TOT-Aris	171	1 yr	Success	Same as above	NR	73.10%
Abdel-fattah 2012	#1904	TOT-Aris	171	1 yr	Improvement QoL	Based on 18-point improvement in KHQ scores as the cut-off value	NR	69.60%
Freeman 2011	#2539	Monarc TOT	100	12m	Cure	Absence of SUI	NR	63.4%
Houwert 2010	#2950	TOT MONARC	95	12m	Cure	Statement of the woman of not experiencing any loss of urine upon physical activity, coughing, or sneezing.	NR	74%

Study name	Endnote	Intervention	N	Follow -up period	Type of outcome	Outcome description	With or without containment products	% of patients with outcome
Houwert 2009	#3050	TOT MONARC	86	12m	Cure	Statement of the woman of not experiencing any loss of urine upon physical exercise.	NR	77%
Houwert 2009	#3050	TOT MONARC	86	12m	Improvement	NR	NR	17%
Houwert 2009	#3050	TOT MONARC	86	2-4y	Cure	Statement of the woman of not experiencing any loss of urine upon physical exercise.	NR	65%
Houwert 2009	#3050	TOT MONARC	86	2-4y	Improvement	NR	NR	21%
Houwert 2009	#10629	TOT MONARC	95	14m	Cure	Statement of the woman of not experiencing any loss of urine on physical exercise.	NR	74%
Barry 2008	#3620	Monarc TOT	58	3m	Objective Cure	NR	NR	82.8%
Barry 2008	#3620	Monarc TOT	58	3m	Subjective Cure	NR	NR	72.4%
Schweitzer 2016	#1199	TOT	44	12 m	Objective cure	Objective cure was defined as a negative stress cough test at a bladder volume of at least 300 mL	NR	39/44 (88.6%)
Schweitzer 2016	#1199	TOT	48	12 m	Subjective cure	Subjective cure defined as a negative answer to the Urogenital Distress Inventory question “Do you experience urine leakage related to physical activity, coughing or sneezing?”	NR	35/48 (72.9%)
Schweitzer 2016	#1199	TOT	44	12 m	Improvement	Much or very much improvement on the Patient Global Impression of Improvement scale	NR	40/44 (87%)
Lee 2015	#5274	Transobturator midurethral sling +/- POP repair	95	6 mo	Objective cure	Negative urodynamic stress or cough stress test	NR	86.3%
Lee 2015	#5274	Transobturator midurethral sling +/- POP repair	90	12 mo	Objective cure	Negative urodynamic stress or cough stress test	NR	96.7%
Lee 2015	#5274	Transobturato	107	6 mo	Subjective	No recorded leakage with coughing and exercise	NR	92.5%

Study name	Endnote	Intervention	N	Follow -up period	Type of outcome	Outcome description	With or without containment products	% of patients with outcome
		r midurethral sling +/- POP repair			cure	on questions 3 and 5 of the Internat-ional Consultation on Incontinence Question-naire-Urinary Incontinence Short Form		
Lee 2015	#5274	Transobturator midurethral sling +/- POP repair	103	12 mo	Subjective cure	Same as above	NR	94.2%
Lee 2015	#5274	Transobturator midurethral sling no POP repair	51	6 mo	Objective cure	Negative urodynamic stress or cough stress test	NR	84.3%
Lee 2015	#5274	Transobturator midurethral sling no POP repair	45	12 mo	Objective cure	Negative urodynamic stress or cough stress test	NR	93.3%
Lee 2015	#5274	Transobturator midurethral sling no POP repair	56	6 mo	Subjective cure	No recorded leakage with coughing and exercise on questions 3 and 5 of the Internat-ional Consultation on Incontinence Question-naire-Urinary Incontinence Short Form	NR	92.9%
Lee 2015	#5274	Transobturator midurethral sling no POP repair	57	12 mo	Subjective cure	Same as above	NR	90.7%
Van Leijsen 2013	#9658	Surgery	55	12 mo	Subjective PGI-I cure	Improved, very much and much improved	NR	91%
Van Leijsen 2013	#9658	Surgery	58	12mo	Subjective UDI cure SUI	Negative answer on the Urogenital Distress Inventory question concerning urine leakage related to physical activities	NR	74%
Van Leijsen	#9658	Surgery	58	12mo	Subjective UDI	No mixed UI	NR	71%

Study name	Endnote	Intervention	N	Follow -up period	Type of outcome	Outcome description	With or without containment products	% of patients with outcome
2013					cure MUI			
Van Leijsen 2013	#9658	Surgery	53	12mo	Objective cure	No Leakage on 48 h bladder diary	NR	85%
Van Leijsen 2013	#9658	Individually tailored	57	12 mo	Subjective PGI-I cure	Improved, very much and much improved	NR	91%
Van Leijsen 2013	#9658	Individually tailored	56	12mo	Subjective UDI cure SUI	Negative answer on the Urogenital Distress Inventory question concerning urine leakage related to physical activities	NR	75%
Van Leijsen 2013	#9658	Individually tailored	55	12mo	Subjective UDI cure MUI	No mixed UI	NR	68%
Van Leijsen 2013	#9658	Individually tailored	50	12mo	Objective cure	No Leakage on 48 h bladder diary	NR	82%
Van Leijsen 2013	#9658	Individual according to urodynamic findings	325	12mo	Subjective PGI-I cure	Improved, very much and much improved	NR	92.6%
Van Leijsen 2013	#9658	Individual according to urodynamic findings	335	12mo	Subjective UDI cure SUI	Negative answer on the Urogenital Distress Inventory question concerning urine leakage related to physical activities	NR	72.2%
Van Leijsen 2013	#9658	Individual according to urodynamic findings	335	12mo	Subjective UDI cure MUI	No mixed UI	NR	67%
North 2010	#2955	Minitape-TM	47	6m	Cure	Negative pad test (weight gain of <1 g).	With	27.7%
North 2010	#2955	Minitape-TM	31	12m	Cure	Negative pad test (weight gain of <1 g).	With	32.3%
North 2010	#2955	Minitape-TM	19	24m	Cure	Negative pad test (weight gain of <1 g).	With	31.6%
Primus 2006	#4000	SPARC sling	89	3m	Objective cure	Negative cough stress test and negative pad test	With	83.1%

Study name	Endnote	Intervention	N	Follow -up period	Type of outcome	Outcome description	With or without containment products	% of patients with outcome
						(0–1 g)		
Primus 2006	#4000	SPARC sling	79	6m	Objective cure	Same as above	With	82.3%
Primus 2006	#4000	SPARC sling	64	12m	Objective cure	Same as above	With	84.4%
Primus 2006	#4000	SPARC sling	89	3m	Subjective cure	No urine loss during daily activities/no usage of pads.	With	79.8%
Primus 2006	#4000	SPARC sling	79	6m	Subjective cure	Same as above	With	81%
Primus 2006	#4000	SPARC sling	64	12m	Subjective cure	Same as above	With	75%
Palomba 2014	#4757	r-TVT	117	6mo	Subjective	Proportion of women who reported being either ‘‘dry’’ or ‘‘improved,’	NR	94.9%
Palomba 2014	#4757	r-TVT	115	12mo	Subjective	Same as above	NR	83.5%
Palomba 2014	#4757	r-TVT	111	18mo	Subjective	Same as above	NR	82.9%
Palomba 2014	#4757	r-TVT	106	24mo	Subjective	Same as above	NR	84.0%
Palomba 2014	#4757	r-TVT	117	6mo	Objective cure	No leakage of urine during the stress test	NR	89.7%
Palomba 2014	#4757	r-TVT	115	12mo	Objective cure	No leakage of urine during the stress test	NR	86.1%
Palomba 2014	#4757	r-TVT	111	18mo	Objective cure	No leakage of urine during the stress test	NR	80.1%
Palomba 2014	#4757	r-TVT	106	24mo	Objective cure	No leakage of urine during the stress test	NR	77.4%
Freeman 2011	#2539	Retropubic TVT	92	12m	Cure	Absence of SUI	NR	65.5%
Houwert 2010	#2950	Retropubic TVT	257	12m	Cure	Statement of the woman of not experiencing any loss of urine upon physical activity, coughing, or sneezing.	NR	81%
Houwert 2009	#10629	Retropubic TVT	257	14m	Cure	Statement of the woman of not experiencing any loss of urine on physical exercise.	NR	83%
**Women- Sling**								
Mostafa 2013	#1538	SIMS	68	4 to	Success	Patient reported success rate, defined as “very	NR	85.50%

Study name	Endnote	Intervention	N	Follow -up period	Type of outcome	Outcome description	With or without containment products	% of patients with outcome
				6m		much improved" or "much improved" on the PGI-I		
Mostafa 2013	#1538	SIMS	69	1yr	Success	Patient reported success rate, defined as "very much improved" or "much improved" on the PGI-I	NR	58/69 (84%)
Mostafa 2013	#1538	SIMS	69	1yr	Objectives cure	Negative Cough Stress Test (CST)	NR	56/69 (81.2%)
Mostafa 2013	#1538	SIMS	65	1yr	Improvement QoL	>= 18 points improvement in total King's Health Questionnaire (KHQ) score	NR	50/65 (76.9%)
Schweitzer 2015	#1199	adjustable single-incision sling	87	12 m	Objective cure	Objective cure was defined as a negative stress cough test at a bladder volume of at least 300 mL	NR	79/87 (90.8%)
Schweitzer 2015	#1199	adjustable single-incision sling	92	12 m	Subjective cure	Subjective cure defined as a negative answer to the Urogenital Distress Inventory question "Do you experience urine leakage related to physical activity, coughing or sneezing?"	NR	71/92 (77.2%)
Schweitzer 2015	#1199	adjustable single-incision sling	90	12 m	Improvement	Much or very much improvement on the Patient Global Impression of Improvement scale	NR	81/90 (90%)
NCT01272284 2011	# 14621	Altis® Single Incision Sling System	103	6m	Cure	Negative Cough Stress Test at 6 Months	With pads	92.20%
NCT01272284 2011	# 14621	Altis® Single Incision Sling System	101	12m	Cure	Negative Cough Stress Test at 12 Months	With pads	90.10%
NCT01272284 2011	# 14621	Altis® Single Incision Sling System	103	6m	Improvement	At Least 50% Reduction in 24-hour Pad Weight From Baseline to 6 Months	With pads	85.40%
NCT01272284 2011	# 14621	Altis® Single Incision Sling System	101	12m	Improvement	At Least 50% Reduction in 24-hour Pad Weight From Baseline to 12 Months	With pads	90.10%

Study name	Endnote	Intervention	N	Follow -up period	Type of outcome	Outcome description	With or without containment products	% of patients with outcome
NCT01272284 2011	# 14621	Altis® Single Incision Sling System	92	6m	Improvement	At Least 50% Improvement in Incontinence Via 3-Day Voiding Diary From Baseline to 6 Months	With pads	88.00%
Natale 2014	#4664	Single-Incision-Sling	92	2y	Objectives cure	Negative Cough Stress Test (CST)	NR	83.7%
Natale 2014	#4664	Single-Incision-Sling	92	2y	Subjective on PGI-I	Much or very much improved	NR	81.5%
Palomba 2014	#4757	Single-Incision-Sling	118	6mo	Subjective	Proportion of women who reported being either ‘‘dry’’ or ‘‘improved,’	NR	90.7%
Palomba 2014	#4757	Single-Incision-Sling	113	12mo	Subjective	Same as above	NR	63.7%
Palomba 2014	#4757	Single-Incision-Sling	108	18mo	Subjective	Same as above	NR	56.5%
Palomba 2014	#4757	Single-Incision-Sling	103	24mo	Subjective	Same as above	NR	55.3%
Palomba 2014	#4757	Single-Incision-Sling	118	6mo	Objective cure	No leakage of urine during the stress test	NR	81.3%
Palomba 2014	#4757	Single-Incision-Sling	113	12mo	Objective cure	No leakage of urine during the stress test	NR	63.7%
Palomba 2014	#4757	Single-Incision-Sling	108	18mo	Objective cure	No leakage of urine during the stress test	NR	52.8%
Palomba 2014	#4757	Single-Incision-Sling	103	24mo	Objective cure	No leakage of urine during the stress test	NR	50.5%
Dias 2014	#4991	Single-incision sling	50	1yr	Subjective cure	ICIQ-SF =0	NR	84%
Dias 2014	#4991	Single-incision sling	50	1yr	Subjective improvement	ICIQ-SF lower than the preoperative but >0 AND subjective perception of improvement/ satisfaction	NR	8%
Dias 2014	#4991	Single-incision sling	50	1yr	Objective cure	No leakage of urine during a CST and patient-reported no pad usage	Without	90.2%

Study name	Endnote	Intervention	N	Follow -up period	Type of outcome	Outcome description	With or without containment products	% of patients with outcome
Dias 2014	#4991	Single-incision sling	50	3 mo	Subjective cure	ICIQ-SF =0	NR	88.5%
Dias 2014	#4991	Single-incision sling	50	3 mo	Subjective improvement	ICIQ-SF lower than the preoperative but >0 AND subjective perception of improvement/ satisfaction	NR	5.8%
Dias 2014	#4991	Single-incision sling	50	3mo	Objective cure	No leakage of urine during a CST and patient-reported no pad usage	Without	94.1%
Dias 2014	#4991	Single-incision sling	50	6 mo	Subjective cure	ICIQ-SF =0	NR	82.0%
Dias 2014	#4991	Single-incision sling	50	6 mo	Subjective improvement	ICIQ-SF lower than the preoperative but >0 AND subjective perception of improvement/ satisfaction	NR	12%
Dias 2014	#4991	Single-incision sling	50	6 mo	Objective cure	No leakage of urine during a CST and patient-reported no pad usage	Without	89.8%
Lee 2015	#5274	Single-incision sling	95	6 mo	Objective cure	Negative urodynamic stress or cough stress test	NR	81.1%
Lee 2015	#5274	Single-incision sling	89	12 mo	Objective cure	Negative urodynamic stress or cough stress test	NR	94.4%
Lee 2015	#5274	Single-incision sling	110	6 mo	Subjective cure	No recorded leakage with coughing and exercise on questions 3 and 5 of the Internat-ional Consultation on Incontinence Question-naire-Urinary Incontinence Short Form	NR	95.5%
Lee 2015	#5274	Single-incision sling	103	12 mo	Subjective cure	Same as above	NR	92.2%
Lee 2015	#5274	Single-incision sling no POP repair	58	6 mo	Objective cure	Negative urodynamic stress or cough stress test	NR	81%
Lee 2015	#5274	Single-incision sling no POP repair	51	12 mo	Objective cure	Negative urodynamic stress or cough stress test	NR	92.2%

Study name	Endnote	Intervention	N	Follow-up period	Type of outcome	Outcome description	With or without containment products	% of patients with outcome
Lee 2015	#5274	Single-incision sling no POP repair	66	6 mo	Subjective cure	No recorded leakage with coughing and exercise on questions 3 and 5 of the Internat-ional Consultation on Incontinence Question-naire-Urinary Incontinence Short Form	NR	95.5%
Lee 2015	#5274	Single-incision sling no POP repair	62	12 mo	Subjective cure	Same as above	NR	91.9%
Cornu 2012	#1969	Ajust-TM single incision transobturator sling procedure	95	6m	Success	No pad use or one dry security pad.	Without and with containment products	83%
Cornu 2012	#1969	Ajust-TM single incision transobturator sling procedure	95	12m	Success	No pad use or one dry security pad.	Without and with containment products	80%
Cornu 2012	#1969	Ajust-TM single incision transobturator sling procedure	90	18m	Success	No pad use or one dry security pad.	Without and with containment products	95%
Guerrero 2010	#2702	Autologous fascial sling	79	6m	Cure	Completely dry	NR	44%
Guerrero 2010	#2702	Autologous fascial sling	79	12m	Cure	Completely dry	NR	41%
Guerrero 2010	#2702	Autologous fascial sling	79	6m	Improvement	Either completely dry or who reported that their urinary incontinence was improved at 6 weeks	NR	87%
Guerrero 2010	#2702	Autologous	79	12m	Improvement	Either completely dry or who reported that their	NR	76%

Study name	Endnote	Intervention	N	Follow -up period	Type of outcome	Outcome description	With or without containment products	% of patients with outcome
		fascial sling				urinary incontinence was improved at 6 weeks		
**Women- Colposuspension**								
Carey 2006	#11686	Colposuspension Burch open	92	6mo	Cure objective	no SUI on urodynamics	NR	22%
Carey 2006	#11686	Colposuspension Burch open	92	6mo	Cure objective	no SUI or detrusor overactivity on urodynamics	NR	32%
Carey 2006	#11686	Colposuspension Burch open	90	24 mo	Cure self report	no SUI	NR	70%
Carey 2006	#11686	Colposuspension Burch open	90	24 mo	Cure self report	no urge incontinence	NR	50.6%
Carey 2006	#11686	Colposuspension Burch laparoscopic	83	6mo	Cure objective	no SUI on urodynamics	NR	28%
Carey 2006	#11686	Colposuspension Burch laparoscopic	83	6mo	Cure objective	no SUI or detrusor overactivity on urodynamics	NR	36%
Carey 2006	#11686	Colposuspension Burch laparoscopic	77	24 mo	Cure self report	no SUI	NR	62%
Carey 2006	#11686	Colposuspension Burch laparoscopic	77	24 mo	Cure self report	No urge incontinence	NR	43%
Ward 2008	#3630	Colposuspension	49	5y	Cure	Negative 1-hour pad test (<1 g change in weight)	With	90%
Ward 2008	#3630	Colposuspension	79	5y	Subjective Cure	Positive response to BFLUTS questionnaire item 8	With	70%
**Women- Other**								
Houwert 2009	#10629	All surgeries	437	14m	Cure	Statement of the woman of not experiencing any	NR	79%

Study name	Endnote	Intervention	N	Follow -up period	Type of outcome	Outcome description	With or without containment products	% of patients with outcome
		together				loss of urine on physical exercise.		
**Women – PFMT (Supervised)**								
Labrie 2013	#13133	Physiotherapy	174	12 mo	Subjective cure	Negative response to the question, "Do you experience urine leakage related to physical activity, coughing, or sneezing?"	NR	53.4%
Labrie 2013	#13133	Physiotherapy	160	12 mo	Objective cure	No incontinence during a cough stress test at a bladder volume of at least 300 ml	NR	58.8%
Labrie 2013	#13133	Physiotherapy	174	12 mo	PGI-S cure	No symptoms	NR	65.5%
Labrie 2013	#13133	Physiotherapy	174	12 mo	PGI-I Improvement	Much better or very much better	NR	64.4%
Labrie 2013	#13133	Physiotherapy	189	4mo	PGI-S cure	No symptoms	NR	31.2%
Labrie 2013	#13133	Physiotherapy	190	4mo	PGI-I Improvement	Much better or very much better	NR	31.1%
Labrie 2013	#13133	Physiotherapy	182	6mo	PGI-S cure	No symptoms	NR	41.8%
Labrie 2013	#13133	Physiotherapy	182	6mo	PGI-I Improvement	Much better or very much better	NR	44.5%
Labrie 2013	#13133	Physiotherapy	159	18mo	PGI-S cure	No symptoms	NR	73.6%
Labrie 2013	#13133	Physiotherapy	159	18mo	PGI-I Improvement	Much better or very much better	NR	74.8%
Liebergall-Wischnitzer 2013	#3257	PFMT	79	6m	Subjective Cure	No pad use on telephone interview	With	39.7%
Kondo 2007	#3822	PFMT	112	2y	Subjective treatment success	Self-reported completely cured or more than 50% improved	NR	40%
Kondo 2007	#3822	PFMT	79	8y	Subjective cure	Self-reported completely cured.	NR	17%
Kondo 2007	#3822	PFMT	79	8y	Subjective	Self-reported more than 50% improvement.	NR	23%

Study name	Endnote	Intervention	N	Follow -up period	Type of outcome	Outcome description	With or without containment products	% of patients with outcome
					improvement			
Kondo 2007	#3822	PFMT	79	8y	Subjective treatment success	Self-reported completely cured or more than 50% improved	NR	39%
Williams 2006	#4019	Intensive PFMT	77	3m	Cure	No symptoms	NR	5%
Lee 2013	#1656	PFMT+biofeed back	71	12 wks	Objective cure	2 g or less of leakage with a standardized bladder volume	NR	52.9%
Lee 2013	#1656	PFMT+biofeed back	56	12m	Subjective cure	Questionnaire- if experienced incontinence	NR	26.8%
Liebergall-Wischnitzer 2013	#3257	Paula method	64	6m	Subjective Cure	No pad use on telephone interview	With	37.1%
**Women – PFMT (Unsupervised)**								
Sjostrom 2015	#15548	Internet based PFMT	105	4m	Improvement on PGI-I	Very much or much improved	NR	40.9%
Sjostrom 2015	#15548	Postal based PFMT	113	4m	Improvement on PGI-I	Very much or much improved	NR	26.5%
Sjostrom 2015	#15548	Internet based PFMT	105	4m	Improvement - satisfaction	Good or very good by patient	NR	84.8%
Sjostrom 2015	#15548	Postal based PFMT	113	4m	Improvement - satisfaction	Good or very good by patient	NR	62.9%
Sjostrom 2015	#15548	Internet based PFMT	105	4m	Improvement - aids usage	Stopped using or reduced their usage of UI aids	With pads	59.5%
Sjostrom 2015	#15548	Postal based PFMT	113	4m	Improvement - aids usage	Stopped using or reduced their usage of UI aids	With pads	41.4%
Sjostrom 2015	#15548	Internet based PFMT	88	1y	Improvement on PGI-I	Very much or much improved	NR	31.9%

Study name	Endnote	Intervention	N	Follow-up period	Type of outcome	Outcome description	With or without containment products	% of patients with outcome
Sjostrom 2015	#15548	Postal based PFMT	80	1y	Improvement on PGI-I	Very much or much improved	NR	33.8%
Sjostrom 2015	#15548	Internet based PFMT	86	1y	Improvement - satisfaction	Good or very good by patient	NR	69.8%
Sjostrom 2015	#15548	Postal based PFMT	76	1y	Improvement - satisfaction	Good or very good by patient	NR	60.5%
Sjostrom 2015	#15548	Internet based PFMT	74	2y	Improvement on PGI-I	Very much or much improved	NR	39.2%
Sjostrom 2015	#15548	Postal based PFMT	80	2y	Improvement on PGI-I	Very much or much improved	NR	23.8%
Sjostrom 2015	#15548	Internet based PFMT	74	2y	Improvement - satisfaction	Good or very good by patient	NR	64.9%
Sjostrom 2015	#15548	Postal based PFMT	79	2y	Improvement - satisfaction	Good or very good by patient	NR	58.2%
**Women- Vaginal cone therapy**								
Williams 2006	#4019	Vaginal cone therapy	79	3m	Cure	No symptoms	NR	9%
**Women-Lifestyle Advice (Unsupervised)**								
Williams 2006	#4019	Continued primary behaviour intervention	75	3m	Cure	No symptoms	NR	8%
**Women – Bulking agents**								
Ghoniem 2009	#3326	Bulking agent-Macroplastique	122	12 mo	Success	Stamey scale dry (0)	NR	36.9%
Ghoniem 2009	#3326	Bulking agent-Macroplastiqu	122	12 mo	Improvement	Stamey scale 1 grade	NR	61.5%

Study name	Endnote	Intervention	N	Follow-up period	Type of outcome	Outcome description	With or without containment products	% of patients with outcome
		e						
Ghoniem 2009	#3326	Bulking agent-Macroplastique	102	12 mo	Success subjective patient	Subjective cured	NR	33.3%
Ghoniem 2009	#3326	Bulking agent-Macroplastique	102	12 mo	Improvement subjective patient	Subjective marked improvement	NR	44.1%
Ghoniem 2009	#3326	Bulking agent-Macroplastique	102	12 mo	Improvement subjective patient	Subjective slight improvement	NR	14.7%
Ghoniem 2009	#3326	Bulking agent-Macroplastique	102	12 mo	Success subjective physician	Subjective cured	NR	42.2%
Ghoniem 2009	#3326	Bulking agent-Macroplastique	102	12 mo	Improvement subjective physician	Subjective marked improvement	NR	38.2%
Ghoniem 2009	#3326	Bulking agent-Macroplastique	102	12 mo	Improvement subjective physician	Subjective slight improvement	NR	13.7%
Ghoniem 2009	#3326	Bulking agent-Macroplastique	67	12 mo	Success	Stamey scale dry (0)	NR	57%
Ghoniem 2009	#3326	Bulking agent-Macroplastique	67	12 mo	Improvement	Stamey scale 1 grade	NR	43%
Ghoniem 2009	#3326	Bulking agent-Macroplastique	67	12 mo	Success subjective patient	Subjective cured	NR	43%

Study name	Endnote	Intervention	N	Follow -up period	Type of outcome	Outcome description	With or without containment products	% of patients with outcome
Ghoniem 2009	#3326	Bulking agent-Macroplastique	67	12 mo	Improvement subjective patient	Subjective marked improvement	NR	48%
Ghoniem 2009	#3326	Bulking agent-Macroplastique	67	12 mo	Improvement subjective patient	Subjective slight improvement	NR	9%
Ghoniem 2009	#3326	Bulking agent-Macroplastique	67	12 mo	Objective improvement pad weight	>= 50% decrease in urine loss	NR	81%
Ghoniem 2009	#3326	Bulking agent - Contigen	125	12 mo	Success	Stamey scale dry (0)	NR	24.8%
Ghoniem 2009	#3326	Bulking agent - Contigen	125	12 mo	Improvement	Stamey scale 1 grade	NR	48%
Ghoniem 2009	#3326	Bulking agent - Contigen	94	12 mo	Success subjective patient	Subjective cured	NR	26.6%
Ghoniem 2009	#3326	Bulking agent - Contigen	94	12 mo	Improvement subjective patient	Subjective marked improvement	NR	41.5%
Ghoniem 2009	#3326	Bulking agent - Contigen	94	12 mo	Improvement subjective patient	Subjective slight improvement	NR	20.2%
Ghoniem 2009	#3326	Bulking agent - Contigen	94	12 mo	Success subjective physician	Subjective cured	NR	34%
Ghoniem 2009	#3326	Bulking agent - Contigen	94	12 mo	Improvement subjective physician	Subjective marked improvement	NR	40.4%
Ghoniem 2009	#3326	Bulking agent -	94	12 mo	Improvement	Subjective slight improvement	NR	14.9%

Study name	Endnote	Intervention	N	Follow -up period	Type of outcome	Outcome description	With or without containment products	% of patients with outcome
		Contigen			subjective physician			
**Women- Duloxetine**								
Schagen van Leeuwen 2008	#11523	Duloxetine	126	12 wks	Improvement IEF	Responder IEF: ≥50% reduction in Incontinence Episode Frequency (IEF) from baseline to endpoint.	Without	57%
Schagen van Leeuwen 2008	#11523	Duloxetine	128	12 wks	Improvement I-QoL	Responder I-QoL: increase in the I-QOL total score of ≥6.3 points from baseline to endpoint.	Without	55%
Schagen van Leeuwen 2008	#11523	Duloxetine	129	12 wks	Improvement PGI-I	Very much better or much better PGI-I	Without	53.5%
Viktrup 2007	#10965	Duloxetine	409	12 wks	Improvement	Patients aged <50 years. Better in PGI-I	Without	62.5%
Viktrup 2007	#10965	Duloxetine	549	12 wks	Improvement	Patients aged ≥50 years. Better in PGI-I	Without	66.2%
**MEN**								
**Male Sling**								
Zuckerman 2014	#1334	AdVance male sling	102	12m	Success	Success was defined as a dry safety pad or less per day postoperatively (cured) or both a >50% improvement in pad use and patient satisfaction with the surgical outcome (improved).	with pads	76/102 (74%)
Zuckerman 2014	#1334	AdVance male sling	102	24 m	Success	Same as above	with pads	71/102 (70%)
Zuckerman 2014	#1334	AdVance male sling	102	36.2 m	Overall Success	Final follow up. Success was defined as a dry safety pad or less per day postoperatively (cured) or both a >50% improvement in pad use and patient satisfaction with the surgical outcome (improved).	with pads	63/102 (62%)
Zuckerman 2014	#1334	AdVance male sling	102	12 m	Cure	Cure was defined as a dry safety pad or less per day postoperatively (cured)	with pads	59/102 (58%)
Zuckerman 2014	#1334	AdVance male sling	102	24 m	Cure	Cure was defined as a dry safety pad or less per day postoperatively (cured)	with pads	49/102 (48%)
Zuckerman	#1334	AdVance male	102	36.2 m	Overall Cure	Final follow up. Cure was defined as a dry safety	with pads	41/102

Study name	Endnote	Intervention	N	Follow -up period	Type of outcome	Outcome description	With or without containment products	% of patients with outcome
2014		sling				pad or less per day postoperatively (cured)		(40%)
Zuckerman 2014	#1334	AdVance male sling	102	12 m	Improvement	A >50% improvement in pad use and patient satisfaction with the surgical outcome (improved).	with pads	22/102 (22%)
Zuckerman 2014	#1334	AdVance male sling	102	24 m	Improvement	Same as above	with pads	22/102 (22%)
Zuckerman 2014	#1334	AdVance male sling	102	36.2 m	Overall Improvement	Final follow up. A >50% improvement in pad use and patient satisfaction with the surgical outcome (improved).	with pads	22/102 (22%)
Serra 2013	#9689	Male sling	61	3 mo	Objective cure	No pad use	Without	80%
Serra 2013	#9689	Male sling	61	3 mo	Objective improvement	≥50% reduction of 24 h pad weight	NR	8%
Bauer 2009	#2979	AdVance sling	113	6m	Cure	No pad use or one pad that was used for security reasons and that stayed dry during the day.	With	55.8%
Bauer 2009	#2979	AdVance sling	113	6m	Improvement	One to two wet pads or a reduction of pads 50%.	With	27.4%
Bauer 2009	#2979	AdVance sling	70	12m	Cure	No pad use or one pad that was used for security reasons and that stayed dry during the day.	With	51.4%
Bauer 2009	#2979	AdVance sling	70	12m	Improvement	One to two wet pads or a reduction of pads 50%.	With	25.7%
Rehder 2012	#2053	AdVance sling	85	12m	Cure	No pad or one dry pad for security reason	Without and with containment products	53.80%
Rehder 2012	#2053	AdVance sling	151	3y	Cure	No pad or one dry pad for security reason	Without and with containment products	53%
Bochove-Overgaauw 2011	#2490	Argus Sling	95	27m	Cure	0-1 security pad per 24h	With	54%

Study name	Endnote	Intervention	N	Follow -up period	Type of outcome	Outcome description	With or without containment products	% of patients with outcome
Bochove-Overgaauw 2011	#2490	Argus Sling	95	27m	Improvement	1-2 pads per 24h and >50% pad reduction	With	18%
Lanoe 2009	#10419	Sub-urethral sling	84	20 m	Cure	Dry patients	without pads	38/84 (45.2%)
Lanoe 2009	#10419	Sub-urethral sling	84	20 m	Improvement	Patients reporting fewer pad usage than before.	With pads	22/84 (26.2%)
Lanoe 2009	#10419	Sub-urethral sling	84	20 m	Failure	No improvement or condition worsened	With pads	24/84 (28.6%)
**Men – PFMT (Supervised)**								
Filocamo 2007	#11513	PFMT	52	16 wks	Cure	Dry - the use of zero pads	Without	51.9%
Filocamo 2007	#11513	PFMT	52	20 wks	Cure	Dry - the use of zero pads	Without	73%
Filocamo 2007	#11513	PFMT	52	24 wks	Cure	dry - the use of zero pads	Without	78%
Marchiori 2010	#11411	PFMT guided+biofeedback+electrical stimulation	166	1 yr	Success self report	Self report for the recovery of continence = no use of pads or a mild leakage needing 2 mini-pads a day	With	100%
**Men – PFMT (Unsupervised)**								
Marchiori 2010	#11411	PFMT	166	1 yr	Success self report	Same as above	With	100%
**Men – PFMT (Supervised) + duloxetine**								
Filocamo 2007	#11513	PFMT+duloxetine	50	16 wks	Cure	dry - the use of zero pads	Without	78%
Filocamo 2007	#11513	PFMT+duloxetine	50	20 wks	Cure	dry - the use of zero pads	Without	46%
Filocamo 2007	#11513	PFMT+duloxetine	50	24 wks	Cure	dry - the use of zero pads	Without	62%

Table S6: Study characteristics for patients with UUI

Study name	Endnote	N	Inclusion criteria	Mean/ Median age (yrs)	% of males	RoB No. Low *
**WOMEN**						
Chapple 2013[100]	#11434	2382	More than 18 yr of age with symptoms of OAB (urinary frequency and urgency with or without urgency incontinence) >= 3 mo were eligible for the placebo run-in." "patients were eligible for randomization if they met the following criteria during the 3-d micturition diary period: average micturition frequency eight or more times per 24 h and three or more episodes of urgency (grade 3 or 4 using the Patient Perception of Intensity of Urgency Scale) with or without incontinence.	59.2/60.1 /59.6	26%	18
Visco 2012[102]	#15547	119	Women; >=5 episodes of urgency urinary incontinence, as recorded in a 3-day diary, and urgency-predominant urinary incontinence; not previously received anticholinergic drugs or had previously received up to two anticholinergic medications other than solifenacin, darifenacin, or trospium chloride; Ability to perform bladder catheterization by women or caregiver.	56.7	0%	18
Nitti 2013[103]	#9683	280	>=18 years; idiopathic OAB who experienced 3 or more urgency UI episodes in a 3-day period and an average of 8 or more micturitions per day were enrolled in the study; inadequately treated with prior anticholinergic therapy due to inadequate efficacy or intolerable side effects; a PVR of 100 ml or less and willing to perform CIC, if required.	61.7	10.8%	17
Yamaguchi 2007[116]	#11732	713	Men and women aged ≥20 years and with symptoms of OAB reported for ≥6 months were eligible for screening and study enrolment. To be eligible for randomization after the 2-week placebo run-in period, patients had to report a mean number of voids/24 h of ≥8, ≥3 episodes of urgency and/or ≥ 3 episodes of urgency incontinence during a 3-day voiding-diary period.	60	15.6%	16
Chapple 2014[106]	#15555	1955	Men and women aged ≥18 years with self-reported OAB symptoms for ≥6 months before screening; a mean of ≥8 micturitions and ≥2 and ≤15 UUI episodes/24 h (Urinary Sensation Scale [USS] rating of 5) captured in a 3-day diary at baseline who reported that their bladder caused at least some moderate problems on the Patient Perception of Bladder Condition (PPBC); able to complete micturition diaries and study related questionnaires and comply with study procedures.	59	19%	15
Herschorn 2013[108]	#15552	511	>=18 years; OAB symptoms for >= 3 months for run-in and over a 3-day micturition diary period: an average >=8 micturitions per 24 hours and >= 3 urgency episodes (grade 3 or 4 on the 5-point Patient Perception of Intensity of Urgency Scale), with or without incontinence.	60	31.7%	15
Rovner 2011[104]	#11452	269	Males and females; age 18–85 years; >=50 kg weight; symptoms of idiopathic OAB with UUI for >=6 months immediately prior to screening; 8 UUI episodes/week (with no more than 1	60	7.8%	15

Study name	Endnote	N	Inclusion criteria	Mean/ Median age (yrs)	% of males	RoB No. Low *
			incontinence-free day/week); urinary frequency (defined as an average of 8 micturitions/day); not adequately managed with >=1 anticholinergic drug (defined as an inadequate response to or intolerable side effects after >= 1 month of therapy on an optimized dose), in the opinion of the investigator.			
Dmochowski 2008[117]	#10812	267	>=18 years or older with OAB of >=6 months with symptoms of urinary frequency on 30day diary (a mean of >= 10 toilet voids per day), urgency (>=1 episodes of severe urgency associated with a toilet void), and UUI (a mean of 1 or more UUI episodes per day)	61.2	17.9%	15
Tincello 2012[91]	#11438	86	OAB symptoms and DO on urodynamics <=2 yr; refractory to treatment (8wk of treatment with any anticholinergic drug); at least 8 voids and 2 moderate or severe urgency per 24 h on 3-d urinary diary data after stopping oral medication	60.7 (Med)	0	15
Kaplan 2011[118]	#11473	1834	Men and women; ≥ 18 years; self-reported OAB symptoms for ≥3 months and a mean of >= UUI episode and ≥8 micturitions per 24 h in 3-day bladder diaries.	58	15.5%	14
Staskin 2007[119]	#15553	292	Men and women; >=18 years; symptoms of OAB for >= 6 months; Symptoms of urgency (>=1 “severe” urgency severity rating per 3 days, as measured using a validated urgency severity scale (Indevus Urgency Severity Scale); minimum urinary frequency of >= 30 toilet voids per 3 days with an average of >=1 UUI episode/day; Average total volume voided 3,000 ml or less per day and 250 ml or less per void.	59.6	14.8%	14
Rogers 2009[96]	#11496	161	≥18 years; OAB symptoms for ≥3 months; mean of ≥8 micturitions per 24 h, including ≥0.6 UUI episodes and ≥3 OAB micturitions in 5-day bladder diaries; stable sexually active relationship with a male partner for ≥6 months; “some moderate problems” related to their bladder condition on the Patient Perception of Bladder Condition questionnaire	49.5	0	14
Liao 2013[26]	#15559	42	Patients younger than 65 years, diagnosed with urodynamic IDO refractory to previous antimuscarinics for more than 3 months.	44.6	35.7%	14
Herschorn 2010[105]	#11509	1277	Men and women; age ≥18 years; symptoms of OAB (self-assessed) for ≥3 months before screening; a mean of ≥1 UUI episode/24 h and ≥8 voids/24 h in 3-day bladder diaries completed at baseline.	58	17.7%	13
Wyndaele 2009[115]	#10579	474	Adults with OAB for ≥3 months, ≥8 micturitions per 24 h, mean number of urgency episedoes ≥3 per 24 h in a 5-day diary; rating bladder condition as ≥"some moderate problems" on PPBAC who were disatisfied with previous tolterodine or tolterodine ER therapy.	60	23%	13

Study name	Endnote	N	Inclusion criteria	Mean/ Median age (yrs)	% of males	RoB No. Low *
NCT00090584 2013[94]	#13577	307	Female; urge predominant incontinence; incontinent > 3 month; available for 8 months of follow up	56.9	0	13
Staskin 2011[113]	#10091	168	Men and Women (≥18 years) self-reporting OAB symptoms for ≥3 months before screening, including a mean of ≥8 micturitions and ≥3 urgency episodes per 24 hr in a 3-day bladder diary at baseline, who rated their bladder condition at baseline as causing at least “some moderate problems” using the Patient Perception of Bladder Condition (PPBC) questionnaire.	58.8	18%	13
Groen 2011[95]	#2358	60	UUI refractory to conservative management; PNE revealed at least a 50% decrease in the number of incontinence episodes or pads used daily	48 (Med)	0	13
Amundsen 2005[92]	#4293	55	All patients failed previous conservative treatment. All patients met criteria for permanent implantation: a positive response during test stimulation - ≥50% reduction in incontinence episodes	60	7%	13
Duckett 2006[98]	#4217	51	Fifty-one women, who had symptoms of both stress and urge incontinence and were proven to have idiopathic DO and USI on urodynamics, undergo regular review.	52 (Med)	0	13
Chapple 2005[109]	#11576	1177	Men and women; >= 18 years; OAB symptoms (including urinary frequency, urgency or urge incontinence) for >= 3 months; being treated as outpatients; After 2-wk run-in criteria during the 3-day voiding diary period: an average of >=8 micturitions per 24 hours; an average of >= 1 incontinence episode per 24 hours, or an average of 1 urgency episode per 24 hours.	56.5	13%	12
Chapple 2007b[110]	#10668	824	OAB symptoms with urinary urgency for >= 6 mo; >=18 yrs;>= 8 micturitions per 24 h and either >=6 urgency episodes or >= 3 UUI episodes per 24 h; on a Likert scale that the condition caused them at least moderate problems.	57	20%	12
Surwitt 2009[97]	#10558	256	Urge incontinence or mixed incontinence; all received conservative treatment	56 (Med)	0	12
Siegel 2015[93]	#4552	147	Diagnosis of OAB on a 3-day voiding diary with >=8 voids/day and/or >=2 involuntary leaking episodes in 72 hr; age >=18. Failed or are not a candidate for more conservative treatment; Failed or could not tolerate at least one anticholinergic or antimuscarinic medication AND have at least one anticholinergic or antimuscarinic medication not yet attempted; On current regimen of OAB medications or have not been on any OAB medications, for at least 4 weeks prior to beginning the baseline voiding diary	60.4	7%	12
Karram 2009[114]	#10655	229	Ambulatory men and women, age 18 or older, were eligible to participate. For this study, OAB was defined as having at least 1 urgency episode/24 h (on average), with or without urge incontinence, and usually accompanied by frequency (at least 8 micturitions/24 h), nocturia, or	57	15.8%	11

Study name	Endnote	N	Inclusion criteria	Mean/ Median age (yrs)	% of males	RoB No. Low *
			both for at least 3 months, as documented in a 3-day diary before baseline.			
Haab 2006[90]	#11577	466	Patients with overactive bladder (OAB) who completed two 12-week randomized, double-blind, placebo-controlled ‘feeder’ studies; able to complete patient diaries independently and capable of independent toileting	57.3	14.9%	10
Janssen Korea 2013[111]	#14046	309	Participants who fulfilled all of the following criteria in their micturition charts completed for 3 days prior to visit 2 (Baseline): mean voiding frequency greater than or equal to 8 times per 24 hours and mean frequency of urinary urgency greater than or equal to 2 times per 24 hours (urgency means sudden and strong urge to urinate and a urinary sensation scale score greater than or equal to 3 in the micturition chart). Participants with overactive bladder symptoms lasting for 3 months or longer prior to study initiation.	54.9	17.2%	10
Anderson 2006[107]	#11120	790	"Women with OAB aged 18 years and older who documented 21 to 60 UUI episodes per week and an average of ten or more voids per 24 h were recruited for the study. The patients with mixed incontinence (both urge and stress incontinence) were eligible if urge episodes predominated."	60	0%	9
Sand 2012[112]	#6213	280	Idiopathic OAB with ≥3 urgency UI episodes over a 3-day period, and an average of ≥8 micturitions/day. All had been inadequately managed by prior anticholinergic therapy.	61.3	10.8%	7
Staskin 2006[40]	#11558	535	Men and women; >=18 years; symptoms of OAB (including urinary frequency, urgency, or urge incontinence) for 3 months;>= 8 micturitions per 24 hours AND >= 1 urgency episode per 24 hours or >=1 incontinence episode per 24 hours; For extension: had to have completed treatment in the previous double-blind studies <=14 days prior to extension-study entry.	56.4	22%	6
**MEN**						
Glazener 2011[99]	#847	788	Between January, 2005, and September, 2008, we identified men having prostate surgery in 34 UK centres and invited them to receive a screening questionnaire 3 weeks after surgery. Those who reported urinary incontinence in this questionnaire were invited for random assignment into our study groups.	62.4/62.3/68.2/67.9	100%	18
Drake 2015[101]	#15546	1004	Men aged 45 yr, with International Prostate Symptom Score (IPSS) 13, a maximum urinary flow rate (Qmax) of 4.0–12.0 ml/s, ultrasound-estimated prostate size <75 ml, two or more urgency episodes per 24 h (Patient Perception of Intensity of Urgency Scale [PPIUS] grade 3 or 4) and eight or more micturitions per 24 h	65.1	100%	13

*) Number of low risk items in the Risk of Bias assessment (out of 20 items in total – See Table 5)

Table S7: Treatment characteristics in studies for patients with UUI

Study name	Endnote	Treatment	Further details of intervention	Duration of treatment
**WOMEN**				
**Women – Antimuscarinic**				
Siegel 2015	#4552	Antimuscarinic	another antimuscarinic medication according to physician discretion, or restarted the discontinued medication	6 months
Haab 2006	#11577	Darifenacin	darifenacin CR 7.5 mg for 2 weeks, then individualized dosing: permitted to increase dose to 15 mg if required. Thereafter doses could be increased from 7.5 to 15 mg or decreased from 15 to 7.5 mg, as needed	24 months
Staskin 2011	#10091	Fesoterodine	Oral daily 4mg for 2 weeks and 8mg for 10 weeks	12 wks
Wyndaele 2009	#10579	Fesoterodine	All enrolled subjects were treated for 4 weeks with fesoterodine 4 mg qd, taken in the morning. At week 4, dosage could either be maintained at fesoterodine 4 mg qd or increased to 8 mg qd for the remaining 8 weeks of the study.	12 wks
Chapple 2007b	#10668	Fesoterodine	4 mg once daily in the morning	12 wks
Chapple 2007b	#10668	Fesoterodine	8 mg once daily in the morning	12 wks
Kaplan 2011	#11473	Fesoterodine	4mg for the first week followed by fesoterodine 8 mg for the next 11 weeks	12 wks
Herschorn 2010	#11509	Fesoterodine	8mg	12 wks
Chapple 2014	#15555	Fesoterodine	4mg	12 wks
Chapple 2014	#15555	Fesoterodine	8mg	12 wks
Anderson 2006	#11120	Oxybutynine	extended release 10 mg; no prior anticholinergic therapy	12 wks
Anderson 2006	#11120	Oxybutynine	extended release 10 mg; prior anticholinergic therapy	12 wks
Janssen Korea 2013	#14046	Oxybutynin	Starting dose of 10mg orally once daily. Maximum alowed dose will be 30 mg per day.	12 wks
Visco 2012	#15547	Solifenacin	Initiated treatment with solifenacin at a dose of 5 mg daily. Dose escalation was allowed at months 2 and 4 if adequate control of symptoms. With dose escalation at month 2, the dose of solifenacin was increased to 10 mg; if inadequate control of symptoms continued at month 4, the drug was changed to trospium XR at a dose of	6 months

			60 mg.	
Staskin 2006	#11558	Solifenacin	5 mg or 10 mg once daily	40-52 wks
Karram 2009	#10655	Solifenacin	Solifenacin 5 mg for the first 4 weeks. At week 4, dose could be maintained at 5 mg or increased to 10 mg. At week 8, dose could be maintained, increased from 5 to 10 mg, or decreased from 10 to 5 mg.	12 wks
Chapple 2005	#11576	Solifenacin	5 mg OD, after 4 wks allowed to increase to 10 mg	12 wks
Yamaguchi 2007	#11732	Solifenacin	5mg once daily	12 wks
Yamaguchi 2007	#11732	Solifenacin	10mg once daily	12 wks
NCT00090584 2013	#13577	Tolterodine	4mg/d for 10 weeks. Could be reduced to 2mg/d for managing side effects.	10 wks
Rogers 2009	#11496	Tolterodine	tolterodine ER 4 mg, once daily within 4 h of bedtime	24 wks
Chapple 2013	#11434	Tolterodine ER 4 mg	4 mg once daily	12 months
Chapple 2007b	#10668	Tolterodine ER	once daily in the morning	12 wks
Anderson 2006	#11120	Tolterodine	extended release 4 mg; no prior anticholinergic therapy	12 wks
Anderson 2006	#11120	Tolterodine	extended release 4 mg; prior anticholinergic therapy	12 wks
Kaplan 2011	#11473	Tolterodine ER	4 mg	12 wks
Herschorn 2010	#11509	Tolterodine ER	4mg	12 wks
Chapple 2005	#11576	Tolterodine ER	4 mg OD	12 wks
NCT00090584 2013	#13577	Tolterodine+ PFMT	4mg/d for 10 weeks. Could be reduced to 2mg/d for managing side effects and training in pelvic floor muscle control and exercises; behavioural strategies to diminish urgency, suppress bladder contractions and prevent incontinence; delayed voiding; and individualized fluid management.	10 wks
Dmochowski 2008	#10812	Trospium	60 mg QD oral capsules	12 wks
Staskin 2007	#15553	Trospium	60 mg QD	12 wks

**Women – Adrenergic**				
Chapple 2013	#11434	Mirabegron 50 mg	50 mg once daily	12 months
Chapple 2013	#11434	Mirabegron 100 mg	100 mg once daily	12 months
Herschorn 2013	#15552	Mirabegron 25 mg	25 mg once daily	12 wks
Herschorn 2013	#15552	Mirabegron 50 mg	50 mg once daily	12 wks
**Women – Neuromodulation**				
Amundsen 2005	#4293	Sacral neuromodulation	None provided	29 months
Siegel 2015	#4552	SNM	For SNM InterStim1 Therapy systema which required 14 day test stimulation period. If >= 50% improvement from baseline in average leaks/day or voids/day or a return to normal voiding (<8 voids/day)] implantation according to previously described procedure.	6 months
Groen 2011	#2358	Sacral neuromodulation	All leads were implanted via a small mid sacral incision. An INS was placed abdominally in most of the earlier cases and at the buttock site in later cases. Postoperatively the lead position was assessed radiologically.	Surgery (NR)
Surwitt 2009	#10558	Neuromodulation+ PFMRehabilitation (training, biofeedback and stimulation)	percutaneous tibial nerve neuromodulation (Urgent PC, Uroplasty Inc., Minneapolis, MN, USA) and pelvic floor muscle rehabilitation (Evadri System, Hollister Inc., Libertyville, IL, USA)	NR
**Women – TVT**				
Ducket 2006	#4217	TVT	The TVT procedure was performed as described by Ulmsten et al. and spinal anaesthesia was used.	Surgery (NR)
**Women – Botulinum toxin**				
Tincello 2012	#11438	Botulinum toxin	200 IU of onaBoNTA injected in 20 sites	Single procedure
Liao 2013	#15559	Botulinum toxin	OnabotulinumtoxinA 100U	Single procedure
Sand 2012	#6213	OnabotulinumtoxinA	OnabotulinumtoxinA 100 U administered cystoscopically as 20 intradetrusor	Single

			injections of 0.5 mL, sparing the trigone	procedure
Nitti 2013	#9683	OnabotulinumtoxinA	100 U (Botox®) reconstituted with 10 ml normal saline or placebo (10 ml normal saline)	Single procedure
Rovner 2011	#11452	OnabotulinumtoxinA 50U	50 U; Treatment was administered via flexible or rigid cystoscope under local anaesthesia (with or without sedation as per local practice) as 20 intradetrusor injections of 0.5 ml, avoiding the trigone and dome.	Single procedure
Rovner 2011	#11452	OnabotulinumtoxinA 100U	100U; Same	Single procedure
Rovner 2011	#11452	OnabotulinumtoxinA 150 U	150U; Same	Single procedure
Rovner 2011	#11452	OnabotulinumtoxinA 200U	200U; Same	Single procedure
Rovner 2011	#11452	OnabotulinumtoxinA 300 U	300U; Same	Single procedure
**MEN**				
**Men – Antimuscarinic**				
Drake 2015	#15546	Solifenacin and Tamsulosin	One fixed dose combination tablet per day, starting with 4 wk of Soli 6 mg plus Tamsulosin (oral controlled absorption system formulation - TOCAS) 0.4 mg (6 mg FDC) followed by either 6 mg FDC or Soli 9 mg plus TOCAS (9 mg FDC)	52 wks
**MEN - PFMT (Supervised)**				
Glazener 2011	#847	PFMT (after RP)	Four one-to-one sessions held over 3 months with a therapist and received a supplementary MAPS pelvic-floor exercise leaflet, aimed at establishing a home exercise regimen. The therapists were either specialist continence physiotherapists or specialist continence or urology nurses	3m
Glazener 2011	#847	PFMT (after TURP)	Four one-to-one sessions held over 3 months – Same as above	3m
**MEN - Lifestyle Advice (Unsupervised)**				
Glazener 2011	#847	Lifestyle advice (after RP)	Lifestyle advice leaflet that described the influence of fluid intake, caffeine, diet, constipation, fitness, lifting, chest problems, and urinary tract infections on continence. No information was provided in the leaflet about pelvic-floor exercises or techniques for dealing with urgency symptoms. Men having radical prostatectomy are commonly told about pelvic-floor exercises by health-care professionals and information is also widely available in the public domain—eg, via the internet.	3m

Glazener 2011	#847	Lifestyle advice (after TURP)	Lifestyle advice leaflet – Same as above	3m

Table S8: Results of interventions for patients with UUI

Study name	Endnote	Intervention	N	Follow-up period	Type of outcome	Outcome description	With or without containment products	% of patients with outcome
**WOMEN**								
**Women – Antimuscarinic**								
Siegel 2015	#4552	Antimuscarinic	77	6 mo	OAB therapeutic success rate	a 50% improvement in average leaks/day or voids/day from baseline or a return to normal voiding frequency (<8 voids/day)	NR	42%
Siegel 2015	#4552	Antimuscarinic	77	6 mo	OAB therapeutic success rate	Complete continence	NR	21%
Siegel 2015	#4552	Antimuscarinic	74	6 mo	Qol- Urinary symptom interference	International Consultation on Incontinence improved or greatly improved Modular Questionnaire (ICIQ)-OABqol including a single item on urinary symptom interference	NR	44%
Haab 2006	#11577	Antimuscarinic-darifenacin	600	3mo	Improvement - responder 50%	reductions of ≥ 50%, from feeder-study baseline in incontinence episodes/week	NR	76%
Haab 2006	#11577	Antimuscarinic-darifenacin	543	6mo	Improvement - responder 50%	Same as above	NR	78%
Haab 2006	#11577	Antimuscarinic-darifenacin	497	12mo	Improvement - responder 50%	Same as above	NR	79%
Haab 2006	#11577	Antimuscarinic-darifenacin	466	24 mo	Improvement - responder 50%	Same as above	NR	76%

Study name	Endnote	Intervention	N	Follow-up period	Type of outcome	Outcome description	With or without containment products	% of patients with outcome
Haab 2006	#11577	Antimuscarinic-darifenacin	600	3 mo	Improvement - responder 70%	reductions o ≥ 70% from feeder-study baseline in incontinence episodes/week	NR	59%
Haab 2006	#11577	Antimuscarinic-darifenacin	543	6mo	Improvement - responder 70%	Same as above	NR	61.5%
Haab 2006	#11577	Antimuscarinic-darifenacin	497	12 mo	Improvement - responder 70%	Same as above	NR	65%
Haab 2006	#11577	Antimuscarinic-darifenacin	466	24 mo	Improvement - responder 70%	Same as above	NR	62.3%
Haab 2006	#11577	Antimuscarinic-darifenacin	600	3mo	Improvement - responder 90%	reductions of ≥ 90% from feeder-study baseline in incontinence episodes/week	NR	38%
Haab 2006	#11577	Antimuscarinic-darifenacin	543	6mo	Improvement - responder 90%	Same as above	NR	41%
Haab 2006	#11577	Antimuscarinic-darifenacin	497	12mo	Improvement - responder 90%	Same as above	NR	42%
Haab 2006	#11577	Antimuscarinic-darifenacin	466	24mo	Improvement - responder 90%	Same as above	NR	43.8%
Staskin 2011	#10091	Fesoterodine	168	12 wks	Cure	3-Day Diary-Dry rate	Without	63%
Wyndaele 2009	#10579	Fesoterodine	463	12 wks	Improvement TSQ treatment	Somewhat satisfied or very satisfied	Without	79.8%

Study name	Endnote	Intervention	N	Follow-up period	Type of outcome	Outcome description	With or without containment products	% of patients with outcome
					satisfaction			
Wyndaele 2009	#10579	Fesoterodine	463	12 wks	Improvement TSQ treatment satisfaction	Very satisfied	Without	38.4%
Wyndaele 2009	#10579	Fesoterodine	463	12 wks	Improvement PPBC	Improvement	Without	83%
Wyndaele 2009	#10579	Fesoterodine	463	12 wks	Improvement PPBC	Improvement >=2 points in PPBC	Without	59%
Chapple 2007b	#10668	Fesoterodine 4 mg	265	12 wks	Improvement	Greatly improved or improved on 4-point scale	Without	75%
Chapple 2007b	#10668	Fesoterodine 8 mg	276	12 wks	Improvement	Greatly improved or improved on 4-point scale	Without	79%
Chapple 2007b	#10738	Fesoterodine 8 mg	232	12 wks	Improvement	Greatly improved or improved on 4-point scale	Without	82%
Kaplan 2011	#11473	Fesoterodine	908	12 wks	Cure	Dry rate - no UUI episodes in 3 day diary in patientw tih UUI at b-line	Without	62%
Herschorn 2010	#11509	Fesoterodine	619	12 wks	Cure	Diary dry rate	Without	0.64
Herschorn 2010	#11509	Fesoterodine	636	12 wks	Improvement	Improvement on UPS score	NR	0.46
Chapple 2014	# 15555	Fesoterodine 4 mg	790	12 wks	Cure	Diary dry rate	Without	49.2%
Chapple 2014	# 15555	Fesoterodine 8 mg	779	12 wks	Cure	Diary dry rate	Without	57.8%
Chapple 2014	# 15555	Fesoterodine 4 mg	700	12 wks	Improvement	Improvement in Urgency Perception Scale	Without	43%
Chapple 2014	# 15555	Fesoterodine 8 mg	677	12 wks	Improvement	Improvement in Urgency Perception Scale	Without	50.5%

Study name	Endnote	Intervention	N	Follow-up period	Type of outcome	Outcome description	With or without containment products	% of patients with outcome
Anderson 2006	#11120	Oxybutynine, prior	163	12 wks	Cure	No UUI episodes	Without	25.2%
Anderson 2006	#11120	Oxybutynine, prior	163	12 wks	Cure	No incontinence episodes of any type	Without	20%
Janssen Korea 2013	#14046	Oxybutynin	276	12 wks	Improvement	Number of Participants With Change From Baseline in Response to Primary Overactive Bladder (OAB) Symptom Questionnaire (POSQ): Baseline strongly or very strongly and Week 12 Not bothered or slightly bothered.	Without	24.6%
Janssen Korea 2013	#14046	Oxybutynin	277	12 wks	Improvement: Benefit	Strong benefit from treatment at 12 weeks	Without	45.8%
Janssen Korea 2013	#14046	Oxybutynin	277	12 wks	Improvement: Satisfaction	Very satisfactory treatment at 12 weeks	Without	31.4%
Visco 2012	#15547	Solifenacin	119	6 mo	Cure Urgency	Complete resolution of urgency urinary incontinence	NR	13%
Visco 2012	#15547	Solifenacin	119	6 mo	Cure any incontinence	Complete resolution of all incontinence	NR	11%
Visco 2012	#15547	Solifenacin	119	6 mo	Improvement diary	>75% reduction in episodes of urgency urinary incontinence	NR	40%
Visco 2012	#15547	Solifenacin	116	3 mo	Improvement PGI-I	Very much or much improved	NR	51%
Visco 2012	#15547	Solifenacin	116	6 mo	Improvement PGI-I	Very much or much improved	NR	58%
Staskin 2006	#11558	Solifenacin 5 or 10 mg	535	40 to 52 wks	Cure	Achieved continence	NR	58%
Staskin 2006	#11558	Solifenacin 5 or 10 mg	397	40 to 52 wks	Cure	Achieved continence	NR	60%
Staskin 2006	#11558	Solifenacin 5 or 10	925	44 wks	Improvement	Satisfactory improvement (efficacy)	NR	68%

Study name	Endnote	Intervention	N	Follow-up period	Type of outcome	Outcome description	With or without containment products	% of patients with outcome
		mg						
Staskin 2006	#11558	Solifenacin 5 or 10 mg	925	68 wks	Improvement	Satisfactory improvement (efficacy)	NR	74%
Karram 2009	#10655	Solifenacin	229	12 wks	Cure	Proportion of incontinent patients who reported no episodes of incontinence at the end of study.	Without	58%
Chapple 2005	#11576	Solifenacin	NR	12 wks	Cure	Continent (in those incontinent at b-line)	Without	59%
Chapple 2005	#11576	Solifenacin	NR	12 wks	Improvement (in those incontinent at b-line)	Reduction of at least 50% in their incontinence episodes by the end of the study	Without	74%
Yamaguchi 2007	#11732	Solifenacin 5mg	274	12 wks	Cure	Continent at endpoint	Without	56.2%
Yamaguchi 2007	#11732	Solifenacin 10mg	270	12 wks	Cure	Continent at endpoint	Without	59.6%
NCT00090584 2013	#13577	Tolterodine	119	8 m	Success	Not taking drug or receiving other urge UI therapy and not taking a tricyclic antidepressant or duloxetine at 8 months; and a >70% reduction in number of incontinence episodes as compared to baseline.	NR	41/119 (34.5%)
NCT00090584 2013	#13577	Tolterodine	119	8 m	Improvement	Symptom Improvement defined as the number of women who responded "much better" or "better" to question: "Overall, do you feel that you are much better, better, about the same, worse or much worse?"	NR	54/125 (43.2%)

Study name	Endnote	Intervention	N	Follow-up period	Type of outcome	Outcome description	With or without containment products	% of patients with outcome
Rogers 2009	#11496	Tolterodine	161	24 wks	Cure	No UUI in 5-day diaries	NR	70%
Chapple 2013	#11434	Tolterodine ER 4 mg	791	12mo	Improvement	More than 50% decrease from baseline in the mean number of incontinence episodes per 24 h	NR	66.8%
Chapple 2013	#11434	Tolterodine ER 4 mg	791	12mo	Cure	No incontinence episodes (Dry)	NR	45.1%
Chapple 2007b	#10668	Tolterodine ER	283	12 wks	Improvement	Greatly improved or improved on 4-point scale	Without	72%
Chapple 2007b	#10738	Tolterodine ER	229	12 wks	Improvement	Greatly improved or improved on 4-point scale	Without	70%
Anderson 2006	#11120	Tolterodine, prior	177	12 wks	Cure	No UUI episodes	Without	16.4%
Anderson 2006	#11120	Tolterodine, prior	177	12 wks	Cure	No incontinence episodes of any type	Without	13%
Kaplan 2011	#11473	Tolterodine ER	926	12 wks	Cure	Dry rate - no UUI episodes in 3 day diary in patientw tih UUI at b-line	Without	56%
Herschorn 2010	#11509	Tolterodine	626	12 wks	Cure	Diary dry rate	Without	57.2%
Herschorn 2010	#11509	Tolterodine	641	12 wks	Improvement	Improvement on UPS score	NR	0.4
Chapple 2005	#11576	Tolterodine ER	NR	12 wks	Cure	Continent (in those incontinent at b-line)	Without	49%
Chapple 2005	#11576	Tolterodine ER	NR	12 wks	Improvement (in those incontinent at b-line)	Reduction of at least 50% in their incontinence episodes by the end of the study	Without	67%
NCT00090584 2013	#13577	Tolterodine+ PFMT	118	8 m	Success	Same as above	NR	43/118 (36.4%)
NCT00090584 2013	#13577	Tolterodine+ PFMT	118	8 m	Improvement	Same as above	NR	80/166 (69%)

Study name	Endnote	Intervention	N	Follow-up period	Type of outcome	Outcome description	With or without containment products	% of patients with outcome
Dmochowski 2008	#10812	Triospium	267	12 wks	Cure	Dry rate - no UUI episodes in 3 day diary	Without	35.6%
Staskin 2007	#15553	Triospium	292	12 wks	Normalization (Cure)	No UUI episodes and voiding frequency of 8 or fewer voids per day	Without	20.5%
**Women – Adrenergic**								
Chapple 2013	#11434	Mirabegron 50 mg	789	12mo	Cure	No incontinence episodes (Dry)	NR	43.4%
Chapple 2013	#11434	Mirabegron 100 mg	802	12mo	Cure	No incontinence episodes (Dry)	NR	45.8%
Chapple 2013	#11434	Mirabegron 50 mg	789	12mo	Improvement	More than 50% decrease from baseline in the mean number of incontinence episodes per 24 h	NR	63.7%
Chapple 2013	#11434	Mirabegron 100 mg	802	12mo	Improvement	More than 50% decrease from baseline in the mean number of incontinence episodes per 24 h	NR	66.3%
Herschorn 2013	#15552	Mirabegron 50 mg	257	12 wks	Cure	No Incontinence episodes - 100% dry rate in 3-day diary	Without	47.1%
Herschorn 2013	#15552	Mirabegron 25 mg	254	12 wks	Improvement	≥ 50% reduction in incontinence episodese in 3-day diary	Without	72.8%
Herschorn 2013	#15552	Mirabegron 50 mg	257	12 wks	Improvement	≥ 50% reduction in incontinence episodese in 3-day diary	Without	70%
**Women – Neuromodulation**								
Amundsen 2005	#4293	Sacral neuromodulation	55	29 mo	Subjective	No daily leakage on diary	NR	45.5%
Siegel 2015	#4552	SNM	70	6 mo	OAB therapeutic success rate	A 50% improvement in average leaks/day or voids/day from baseline or a return to normal voiding frequency (<8 voids/day)	NR	61%
Siegel 2015	#4552	SNM	70	6 mo	OAB	Complete continence	NR	39%

Study name	Endnote	Intervention	N	Follow-up period	Type of outcome	Outcome description	With or without containment products	% of patients with outcome
					therapeutic success rate			
Siegel 2015	#4552	SNM	51	6 mo	Qol- Urinary symptom interference	International Consultation on Incontinence improved or greatly improved Modular Questionnaire (ICIQ)-OABqol including a single item on urinary symptom interference	NR	86%
Groen 2011	#2358	Sacral neuromodulation	60	5y	Cure	Complete continence	Without	15
Groen 2011	#2358	Sacral neuromodulation	60	5y	Success rate	Decrease of at least 50% in the number of incontinence episodes or pads used daily	With	30
Groen 2011	#2358	Sacral neuromodulation	60	5y	Excellent response	Decrease of at least 90% in the number of incontinence episodes or pads used daily	With	32
Groen 2011	#2358	Sacral neuromodulation	41	10y	Cure	Complete continence	Without	17
Groen 2011	#2358	Sacral neuromodulation	41	10y	Success rate	Decrease of at least 50% in the number of incontinence episodes or pads used daily	With	37
Groen 2011	#2358	Sacral neuromodulation	41	10y	Excellent response	Decrease of at least 90% in the number of incontinence episodes or pads used daily	With	29
Surwitt 2009	#10558	PFMR+Neuromodulation	256	3mo	Success	An absence of incontinent episodes (dry) and an OAB-V8 score <8, indicating no OAB	NR	93%
Surwitt 2009	#10558	PFMR+Neuromodulation	256	3 mo	Improvement	Improvement in incontinence episodes	NR	7%

Study name	Endnote	Intervention	N	Follow-up period	Type of outcome	Outcome description	With or without containment products	% of patients with outcome
Surwitt 2009	#10558	PFMR+Neuromodulation	133	3mo	Success	An absence of incontinent episodes (dry) and an OAB-V8 score <8, indicating no OAB	NR	94%
**Women – TVT**								
Duckett 2006	#4217	TVT	46	6m	Objective cure	Normal detrusor function during filling cystometry	NR	92%
Duckett 2006	#4217	TVT	46	6m	Subjective cure	Information from interview and questionnaire	NR	91%
**Women – Botulinum toxin**								
Tincello 2012	#11438	Botulinum toxin	86	3mo	Cure	Continent on 3 day diary	NR	35%
Tincello 2012	#11438	Botulinum toxin	100	6 mo	Cure	Continent on 3 day diary	NR	31.3%
Liao 2013	#15559	OnabotulinumtoxinA	42	3m	Success	Success rate- A Patient Perception of Bladder Condition decrease of 2 points was considered successful	Without	88.9%
Liao 2013	#15559	OnabotulinumtoxinA	42	6m	Success	Same as above	Without	49.4%
Liao 2013	#15559	OnabotulinumtoxinA	42	12m	Success	Same as above	Without	23.1%
Sand 2012	#6213	OnabotulinumtoxinA	280	12 wks	Improvement	Improvement in Incontinence quality of life (IQoL) total summary scores	Without	60.2%
Nitti 2013	#9683	OnabotulinumtoxinA	280	12 wks	Cure	Continent - 100% reduction in UI episodes	NR	22.9%
Nitti 2013	#9683	OnabotulinumtoxinA	280	12 wks	Improvement in UI episodes	≥50% reduction in UI episodes	NR	57.5%
Nitti 2013	#9683	OnabotulinumtoxinA	280	12 wks	Improvement TBS	Greatly improved or improved on Treatment Benefit Scale	NR	60.8%
Rovner 2011	#11452	OnabotulinumtoxinA 50U	57	12 wks	Cure self report	No UUI episodes on diary (incontinence-free)	NR	15.9%
Rovner 2011	#11452	OnabotulinumtoxinA	54	12 wks	Cure self	No UUI episodes on	NR	29.8%

Study name	Endnote	Intervention	N	Follow-up period	Type of outcome	Outcome description	With or without containment products	% of patients with outcome
		100U			report	diary (incontinence-free)		
Rovner 2011	#11452	OnabotulinumtoxinA 150 U	49	12 wks	Cure self report	No UUI episodes on diary (incontinence-free)	NR	37.0%
Rovner 2011	#11452	OnabotulinumtoxinA 200U	53	12 wks	Cure self report	No UUI episodes on diary (incontinence-free)	NR	40.8%
Rovner 2011	#11452	OnabotulinumtoxinA 300 U	56	12 wks	Cure self report	No UUI episodes on diary (incontinence-free)	NR	50.9%
Rovner 2011	#9646	OnabotulinumtoxinA	277	12 wks	Subjective improvement	Treatment benefit scale according to the patients greatly improved or improved	Without	62.8%
**MEN**								
**Men – Antimuscarinic**								
Drake 2015	#15546	Solifenacin and Tamsulosin	1004	12 mo	Improvement	IPSS Qol ≤3	NR	85.3%
Drake 2015	#15546	Solifenacin and Tamsulosin	1009	12 mo	Improvement	Patients satisfied with efficacy	NR	82.9%
**Men – PFMT (Supervised)**								
Glazener 2011	#847	PFMT (after RP)	196	12m	Cure	No incontinence - no positive response to either “how often do you leak urine?” or “how much urine do you usually leak (whether you wear protection or not)	NR	24%
Glazener 2011	#847	PFMT (after TURP)	194	12m	Cure	No incontinence - no positive response to either “how often do you leak urine?” or “how much urine do you usually leak (whether you wear protection or not)	NR	35%

Study name	Endnote	Intervention	N	Follow-up period	Type of outcome	Outcome description	With or without containment products	% of patients with outcome
**Men – Lifestyle Advice (Unsupervised)**								
Glazener 2011	#847	Lifestyle advice (after RP)	195	12m	Cure	No incontinence - no positive response to either "how often do you leak urine?" or "how much urine do you usually leak (whether you wear protection or not)	NR	23%
Glazener 2011	#847	Lifestyle advice (after TURP)	203	12m	Cure	No incontinence - no positive response to either "how often do you leak urine?" or "how much urine do you usually leak (whether you wear protection or not)	NR	38%

Table S9: Study characteristics for patients with MUI

Study name	Endnote	N	Inclusion criteria	Mean/ Median age (yrs)	% of males	RoB No. Low*
**WOMEN**						
Palomba 2014[86]	#4757	209	p. 304: the diagnosis of SUI/MUI confirmed by urodynamic assessment; incontinent after conservative management, in MUI -persistent, clinically significant SUI under oral antimuscarinic therapy	64.1 /63.8	0	17
Schagen van Leeuwen 2008[88]	#11523	126	Community-dwelling women of ≥65 years with symptoms of SUI or S-MUI for≥3 consecutive months and ≥7 incontinence episodes per week as determined by the stress/urge incontinence questionnaire S/UIQ were eligible. For patients in the S-MUI group, ≥50% of incontinence episodes had to be due to stress.	70.6	0	17
Williams 2006[84]	#4019	238	Women with a urodynamic diagnosis of USI or mixed UI and DO who had already had an 8-week primary-care intervention.	57	0	16
Williams 2005[44]	#11505	224 0	>=1 of the symptoms: "incontinence several times per month or more, or several times a year plus reported impact of symptoms on quality of life; frequency, hourly or more, or 2-hourly plus impact; nocturia, three times per night, or twice a night plus impact; or urgency, very strong or overwhelming, or strong with impact."	NR	38%	15
Natale 2014[85]	#4664	92	Females with primary stress urinary incontinence (SUI) or mixed urinary incontinence with predominant SUI, diagnosed using both the cough stress test and urodynamics. All had unsuccessfully undergone prior pelvic floor training	58	0	15
Glazener 2014[41]	#13156	230	Urinary incontinence 3 months after 'index' delivery	30.2	0	14
Primus 2006[83]	#4000	103	Women who presented with urinary incontinence symptoms, including genuine, recurrent or mixed stress. All patients had unsuccessfully attempted treating their symptoms using pelvic floor exercises. Patients who indicated that these symptoms represented a significant negative impact on their life and who expressed a strong desire to have the operation were included.	60.2	0	14
Dias 2014[87]	#4991	50	age >18 years; a history of clinically pure SUI or MUi with predominantly bothersome SUI symptoms; a positive cough stress test (CST) with clinical hypermobility of the urethra; previous failed or denied conservative therapy	53.2±10.9	0	14
Surwitt 2009[97]	#10558	256	Urge incontinence or mixed incontinence; all received conservative treatment	56 (Med)	0	12
Kondo 2007[82]	#3822	79	In the present investigation a long-term assessment was performed through a questionnaire that	53.2	0	12

Study name	Endnote	N	Inclusion criteria	Mean/ Median age (yrs)	% of males	RoB No. Low*
			was dispatched to 112 women who had been evaluated at the second, medium-term assessment.			
Abdel-Hady 2005[46]	#4495	454	Women with SUI as a main complaint in whom conservative measures, including pelvic floor exercises, or in case of UUI anticholinergics, failed	57 (Med)	0	10
Staskin 2006[40]	#11558	319	A subgroup of 1041 patients with a history of MUI (patients responded 'yes' when asked if they had a history of mixed stress and urge symptoms) were identified for post hoc analysis. In original studies: Outpatient men and women aged ≥ 18 years were included in the study. Patients were required to have a mean of ≥ 8 voids/24 h, and a mean of ≥ 1 UI episode/24 h or a mean of ≥ 1 urgency episode/24 h during the baseline 3-day voiding diary period, to be eligible for study participation	NR	20%	6
**MEN**						
Glazener 2011[99]	#847	788	Between January, 2005, and September, 2008, we identified men having prostate surgery in 34 UK centres and invited them to receive a screening questionnaire 3 weeks after surgery. Those who reported urinary incontinence in this questionnaire were invited for random assignment into our study groups.	62.4/62.3 /68.2/67. 9	100%	18
Manassero 2007[43]	#11477	54	Objectively confirmed urinary incontinence (>2 g of urine loss on 24 hr Pad test); good general condition	66.8	100%	17
Astellas Pharma 2014[45]	#13488	313	Ambulatory; Willing and able to complete study questionnaires; Has not used any medication for over-active bladder symptoms for at least 14 days prior to enrolment; Diagnosed with prostate cancer, treated by Robotic Assisted Radical Prostatectomy; voiding spontaneously; urinary incontinence one week after removal of the indwelling catheter which requires management with 2 to 10 pads inclusive per day (24 hour days) for 7 consecutive days.	60.5	100%	13
Moore 2008[42]	#11474	166	English- or French-speaking men; booked for RRP; could attend weekly PFMT sessions or maintain weekly contact with the research nurse	NR	100%	13

*) Number of low risk items in the Risk of Bias assessment (out of 20 items in total – See Table 5)

Table S10: Treatment characteristics in studies for patients with MUI

Study name	Endnote	Treatment	Further details of intervention	Duration of treatment
**WOMEN – TVT**				
Abdel-Hady 2005	#4495	TVT	TVT performed under spinal anaesthetic according to previously described technique	Single procedure
Palomba 2014	#4757	retropubic TVT	SPARC system (Tegea for AMS)	Single procedure
Primus 2006	#4000	SPARC sling system	The SPARC Female Sling System uses a suprapubic surgical approach to position the sling under the midurethra, employing 2 thin, curved stainless steel needles advanced via two small incisions above the pubic bone to a vaginal incision below the urethra.	Surgery (42min)
**WOMEN – Sling**				
Natale 2014	#4664	Single-Incision-Sling	Single Incision Sling (AjustTM C.R. Bard Inc., New Providence, NJ, USA)	Single procedure
Palomba 2014	#4757	single-incision mini-slings	Ajust (Bard SpA, Rome, Italy), MiniArc (Tegea for AMS, Bologna, Italy), or TVT Secur System (Johnson & Johnson, Rome, Italy)	Single procedure
Dias 2014	#4991	single-incision sling (SIS)	Altis® (Coloplast)	Single procedure
**WOMEN – PFMT (Supervised)**				
Williams 2006	#4019	Intensive PFMT	Training in PFMT was provided by specially trained nurses, after an initial digital assessment and perineometry. Women were taught how to do correct PF contractions and an individualized exercise regimen was given.	3 months
Kondo 2007	#3822	Pelvic floor muscle training (PFMT)	Conducted for 8 consecutive weeks. Essentially, the training comprised repeated muscle contractions of the pelvic floor and the rapid locking of the perineum just before the abdominal strain. In the training session experienced nurses and doctors supervised the exercises, and they taught how continence was maintained or lost and the treatment rationale was also explained. At home women were instructed to practice the exercise 30 times a day.	8 weeks (60–90 min once a week)
Surwitt 2009	#10558	Tibial Nerve Neuromodulation+	Percutaneous tibial nerve neuromodulation (Urgent PC, Uroplasty	NR

Study name	Endnote	Treatment	Further details of intervention	Duration of treatment
		PFMRehabili-tation (training, biofeedback and stimulation)	Inc., Minneapolis, MN, USA) and pelvic floor muscle rehabilitation (Evadri System, Hollister Inc., Libertyville, IL, USA)	
Williams 2005	#11505	continence service - behavioural and lifestyle/control	4 planned visits over 8 wks, advice on diet and fluids; bladder training; pelvic floor awareness and healthy eating; and treatment for candida and urinary tract infection where indicated.	8 wks
**WOMEN – Vaginal cone therapy**				
Williams 2006	#4019	Vaginal cone therapy	At the first clinical visit after randomization women were instructed how to insert the cones, supervised by the nurse; during this consultation the appropriate weight of cone for starting treatment was identified (Femina, Urohealth Systems Inc., Newport Beach, CA, USA).	3 months
**WOMEN – PFMT for pregnant women with urinary incontinence (3 months after 'index' delivery)**				
Glazener 2014	#13156	Conservative treatment enhanced - mainly PFMT	one-to-one instruction in PFMT, with bladder training if indicated, 3 visits after birth	9 months
**WOMEN – Lifestyle Advice (Supervised)**				
Williams 2006	#4019	Continued primary behaviour intervention	Advice on fluid intake, caffeine intake, bladder re-education, PF awareness (PFA) and weight loss. This was continuation of an 8-week period for all three arms.	3 months
**WOMEN – Antimuscarinics**				
Staskin 2006	#11558	Antimuscarinic-solifenacin	5 or 10 mg	up to 12
**Women- Duloxetine**				
Schagen van Leeuwen 2008	#11523	Duloxetine	Oral 20mg bid for 2 weeks and 40mg bid for 10 weeks	12 wks
**MEN**				
**MEN – Antimuscarinics**				
Astellas Pharma 2014	#13488	Antimuscarinic-solifenacin	5mg	12 wks

Study name	Endnote	Treatment	Further details of intervention	Duration of treatment
**MEN - PFMT (Supervised)**				
Glazener 2011	#847	PFMT (after RP)	Four one-to-one sessions held over 3 months with a therapist and received a supplementary MAPS pelvic-floor exercise leaflet, aimed at establishing a home exercise regimen. The therapists were either specialist continence physiotherapists or specialist continence or urology nurses.	3m
Glazener 2011	#847	PFMT (after TURP)	Four one-to-one sessions held over 3 months with a therapist and received a supplementary MAPS pelvic-floor exercise leaflet, aimed at establishing a home exercise regimen. The therapists were either specialist continence physiotherapists or specialist continence or urology nurses.	3m
Moore 2008	#11474	PFMT guided	standardized daily home routine, weekly 30-minute biofeedback-assisted PFMT (InCare PRS9500, Ontario, Canada)	5.5 mo
Manassero 2007	#11477	PFMT	p. 986: pelvic floor re-education programme; Verbal feedback of the contraction; Initial home practice 45 contractions (3 sessions of 15) per day at home, progressively increasing the until 90 per day.	until incontinence or 1 yr
**MEN – PFMT (Unsupervised)**				
Moore 2008	#11474	PFMT written handout	handout and telephone contact	5.5 mo
**MEN – Lifestyle Advice (Unsupervised)**				
Glazener 2011	#847	Lifestyle advice (after RP)	Lifestyle advice leaflet that described the influence of fluid intake, caffeine, diet, constipation, fitness, lifting, chest problems, and urinary tract infections on continence. No information was provided in the leaflet about pelvic-floor exercises or techniques for dealing with urgency symptoms. Men having radical prostatectomy are commonly told about pelvic-floor exercises by health-care professionals and information is also widely available in the public domain—eg, via the internet.	3m
Glazener 2011	#847	Lifestyle advice (after TURP)	Lifestyle advice leaflet that described the influence of fluid intake,	3m

Study name	Endnote	Treatment	Further details of intervention	Duration of treatment
			caffeine, diet, constipation, fitness, lifting, chest problems, and urinary tract infections on continence. No information was provided in the leaflet about pelvic-floor exercises or techniques for dealing with urgency symptoms. Men having radical prostatectomy are commonly told about pelvic-floor exercises by health-care professionals and information is also widely available in the public domain—eg, via the internet.	

Table S11: Results of interventions for patients with MUI

Study name	Endnote	Intervention	N	Follow-up period	Type of outcome	Outcome description	With or without containment products	% of patients with outcome
**WOMEN – TVT**								
Abdel-Hady 2005	#4495	TVT	454	6 mo	Subjective	Subjective perception of cure	NR	91%
Abdel-Hady 2005	#4495	TVT	454	6 mo	Subjective	Subjective perception improved >50%	NR	8%
Palomba 2014	#4757	r-TVT	117	6mo	Subjective	Proportion of women who reported being either ‘‘dry’’ or ‘‘improved’	NR	94.9%
Palomba 2014	#4757	r-TVT	115	12mo	Subjective	Same as above	NR	83.5%
Palomba 2014	#4757	r-TVT	111	18mo	Subjective	Same as above	NR	82.9%
Palomba 2014	#4757	r-TVT	106	24mo	Subjective	Same as above	NR	84.0%
Palomba 2014	#4757	r-TVT	117	6mo	Objective cure	No leakage of urine during the stress test	NR	89.7%
Palomba 2014	#4757	r-TVT	115	12mo	Objective cure	Same as above	NR	86.1%
Palomba 2014	#4757	r-TVT	111	18mo	Objective cure	Same as above	NR	80.1%
Palomba 2014	#4757	r-TVT	106	24mo	Objective cure	Same as above	NR	77.4%
Primus 2006	#4000	SPARC sling	89	3m	Objective cure	Negative cough stress test and negative pad test (0–1 g)	With	83.10%
Primus 2006	#4000	SPARC sling	79	6m	Objective cure	Same as above	With	82.30%
Primus 2006	#4000	SPARC sling	64	12m	Objective cure	Same as above	With	84.40%
Primus 2006	#4000	SPARC sling	89	3m	Subjective cure	No urine loss during daily activities/no usage of pads.	With	79.80%
Primus 2006	#4000	SPARC sling	79	6m	Subjective cure	Same as above	With	81%
Primus 2006	#4000	SPARC sling	64	12m	Subjective cure	Same as above	With	75%

Study name	Endnote	Intervention	N	Follow-up period	Type of outcome	Outcome description	With or without containment products	% of patients with outcome
**WOMEN – Sling**								
Natale 2014	#4664	Single-Incision-Sling	92	2y	Objectives cure	Negative Cough Stress Test (CST)	NR	83.7%
Natale 2014	#4664	Single-Incision-Sling	92	2y	Subjective on PGI-I	Much or very much improved	NR	81.5%
Palomba 2014	#4757	Single-Incision-Sling	118	6mo	Subjective	Proportion of women who reported being either ‘‘dry’’ or ‘‘improved’	NR	90.7%
Palomba 2014	#4757	Single-Incision-Sling	113	12mo	Subjective	Same as above	NR	63.7%
Palomba 2014	#4757	Single-Incision-Sling	108	18mo	Subjective	Same as above	NR	56.5%
Palomba 2014	#4757	Single-Incision-Sling	103	24mo	Subjective	Same as above	NR	55.3%
Palomba 2014	#4757	Single-Incision-Sling	118	6mo	Objective cure	No leakage of urine during the stress test	NR	81.3%
Palomba 2014	#4757	Single-Incision-Sling	113	12mo	Objective cure	Same as above	NR	63.7%
Palomba 2014	#4757	Single-Incision-Sling	108	18mo	Objective cure	Same as above	NR	52.8%
Palomba 2014	#4757	Single-Incision-Sling	103	24mo	Objective cure	Same as above	NR	50.5%
Dias 2014	#4991	Single-incision sling	50	1yr	Subjective cure	ICIQ-SF =0	NR	84%
Dias 2014	#4991	Single-incision sling	50	1yr	Subjective improvement	ICIQ-SF lower than the preoperative but >0 AND subjective perception of improvement/satisfaction	NR	8%
Dias 2014	#4991	Single-incision sling	50	1yr	Objective cure	No leakage of urine during a CST and patient-reported no pad usage	Without	90.2%
Dias 2014	#4991	Single-incision sling	50	3 mo	Subjective cure	ICIQ-SF =0	NR	88.5%
Dias 2014	#4991	Single-incision sling	50	3 mo	Subjective improvement	ICIQ-SF lower than the preoperative but >0 AND subjective perception of improvement/satisfaction	NR	5.8%

Study name	Endnote	Intervention	N	Follow-up period	Type of outcome	Outcome description	With or without containment products	% of patients with outcome
Dias 2014	#4991	Single-incision sling	50	3mo	Objective cure	No leakage of urine during a CST and patient-reported no pad usage	Without	94.1%
Dias 2014	#4991	Single-incision sling	50	6 mo	Subjective cure	ICIQ-SF =0	NR	82.0%
Dias 2014	#4991	Single-incision sling	50	6 mo	Subjective improvement	ICIQ-SF lower than the preoperative but >0 AND subjective perception of improvement/satisfaction	NR	12%
Dias 2014	#4991	Single-incision sling	50	6 mo	Objective cure	No leakage of urine during a CST and patient-reported no pad usage	Without	89.8%
**WOMEN – PFMT (Supervised)**								
Williams 2006	#4019	Intensive PFMT	77	3m	Cure	No symptoms	NR	5
Kondo 2007	#3822	Pelvic floor muscle training (PFMT)	112	2y	Subjective treatment success	Self-reported completely cured or more than 50% improved	NR	40%
Kondo 2007	#3822	Pelvic floor muscle training (PFMT)	79	8y	Subjective cure	Self-reported completely cured.	NR	17%
Kondo 2007	#3822	Pelvic floor muscle training (PFMT)	79	8y	Subjective improvement	Self-reported more than 50% improvement.	NR	23%
Kondo 2007	#3822	Pelvic floor muscle training (PFMT)	79	8y	Subjective treatment success	Self-reported completely cured or more than 50% improved	NR	39%
Surwitt 2009	#10558	PFMR+Neuromodulation	256	3mo	Success	An absence of incontinent episodes (dry) and an OAB-V8 score <8, indicating no OAB	NR	93%
Surwitt 2009	#10558	PFMR+Neuromodulation	256	3 mo	Improvement	Improvement in incontinence episodes	NR	7%

Study name	Endnote	Intervention	N	Follow-up period	Type of outcome	Outcome description	With or without containment products	% of patients with outcome
Surwitt 2009	#10558	PFMR+Neuromodulation	123	3mo	Success	An absence of incontinent episodes (dry) and an OAB-V8 score <8, indicating no OAB	NR	91%
Surwitt 2009	#10558	PFMR+Neuromodulation	133	3mo	Success	Same as above	NR	94%
Williams 2005	#11505	continence service - behavioural and lifestyle/control	2378	3 mo	CUre	No symptoms	NR	25%
Williams 2005	#11505	continence service - behavioural and lifestyle/control	2201	6 mo	Cure	No symptoms	NR	28%
Williams 2005	#11505	continence service - behavioural and lifestyle/control	2378	3 mo	Improvement	Improvement in one or more symptoms assessed using validated symptom severity quesstions: of incontinence, urgency, frequency and nocturia	NR	60%
Williams 2005	#11505	continence service - behavioural and lifestyle/control	2201	6 mo	Improvement	Same as above	NR	62%
Williams 2005	#11505	continence service - behavioural and lifestyle/control	2468	3 mo	Improvement subjective perception	Self reporting mild problem or no problem	NR	74%
Williams 2005	#11505	continence service - behavioural and lifestyle/control	2181	6 mo	Improvement subjective perception	Same as above	NR	79%
**WOMEN – Vaginal cone therapy**								
Williams 2006	#4019	Vaginal cone therapy	79	3m	Cure	No symptoms	NR	9
**WOMEN – PFMT for pregnant women with urinary incontinence (3 months after 'index' delivery)**								
Glazener 2014	#13156	Conservative, mainly	230	12 yrs	Cure	No urinary incontinence 12 y after	NR	17%

Study name	Endnote	Intervention	N	Follow-up period	Type of outcome	Outcome description	With or without containment products	% of patients with outcome
		PFMT				intervention		
**WOMEN – Lifestyle Advice (Supervised)**								
Williams 2006	#4019	Continued primary behaviour intervention	75	3m	Cure	No symptoms	NR	8
**WOMEN – Antimuscarinics**								
Staskin 2006	#11558	Antimuscarinic-solifenacin	319	52 wks	Cure	no episodes of UI or symptomatic urgency during their final 3-day diary period	NR	52%
**WOMEN- Duloxetine**								
Schagen van Leeuwen 2008	#11523	Duloxetine	126	12 wks	Improvement IEF	Responder IEF: ≥50% reduction in Incontinence Episode Frequency (IEF) from baseline to endpoint.	Without	57%
Schagen van Leeuwen 2008	#11523	Duloxetine	128	12 wks	Improvement I-QoL	Responder I-QoL: increase in the I-QOL total score of ≥6.3 points from baseline to endpoint.	Without	55%
Schagen van Leeuwen 2008	#11523	Duloxetine	129	12 wks	Improvement PGI-I	Very much better or much better PGI-I	Without	53.5%
**MEN**								
**MEN – Antimuscarinics**								
Astellas Pharma 2014	#13488	Solifenacin	313	12 wks	Cure	Gained continence -completely dry	Without	26.5 %
**MEN – PFMT (Supervised)**								
Glazener 2011	#847	PFMT (after RP)	196	12m	Cure	No incontinence - no positive response to either “how often do you leak urine?” or “how much urine do you usually leak (whether you wear protection or not)	NR	24%

Study name	Endnote	Intervention	N	Follow-up period	Type of outcome	Outcome description	With or without containment products	% of patients with outcome
Glazener 2011	#847	PFMT (after TURP)	194	12m	Cure	No incontinence – same as above	NR	35%
Manassero 2007	#11477	PFMT	54	12 mo	Cure	Not incontinent on pad test	NR	83.4%
Manassero 2007	#11477	PFMT	54	6mo	Cure	Same as above	NR	66.7%
Manassero 2007	#11477	PFMT	54	3 mo	Cure	Same as above	NR	46.3%
Moore 2008	#11474	PFMT guided	94	16 wk	Cure	Continent -8 g or less of urine loss on a 24-hour pad test	NR	44%
Moore 2008	#11474	PFMT guided	87	28 wk	Cure	Same as above	NR	47%
Moore 2008	#11474	PFMT guided	89	1 yr	Cure	Same as above	NR	60%
**MEN – PFMT (Unsupervised)**								
Moore 2008	#11474	PFMT handout	80	16 wk	Cure	Same as above	NR	40%
Moore 2008	#11474	PFMT handout	74	28 wk	Cure	Same as above	NR	50%
Moore 2008	#11474	PFMT handout	77	1 yr	Cure	Same as above	NR	64%
**MEN – Lifestyle Advice (Unsupervised)**								
Glazener 2011	#847	Lifestyle advice (after RP)	195	12m	Cure	No incontinence - no positive response to either “how often do you leak urine?” or “how much urine do you usually leak (whether you wear protection or not)	NR	23%
Glazener 2011	#847	Lifestyle advice (after TURP)	203	12m	Cure	No incontinence – same as above	NR	38%

Table S12: Study characteristics for patients with FI

Study name	Endnote	N	Inclusion criteria	Mean/ Median age (yrs)	% of males	RoB No. Low*
**WOMEN**						
Leroi 2012[16]	#9773	66	Patients with FI>3 months; failure of conservative treatments; not a candidate for conservative treatments; ≥ 18 years of age	60 (med)	7%	18
Damon 2014[34]	#1373	157	All patients aged between 18 and 85 years who attended the outpatient clinics of these departments for AI were invited to participate in the trial. Symptoms had to have been present for at least the last 6 months (i.e. at least a gas or liquid leak once a week for at least 6 months with impact on quality of life), and patients had to have a Cleveland Clinic Faecal Incontinence score (CCFI, Jorge & Wexner score) ≥5	61	23%	17
Graf 2011[29]	#10132	125	Age 18–75 years; FI by CCFIS (Cleveleand CLinic Florida Incontinence score) >=9; at least four recorded incontinence episodes in 2 weeks; symptoms >=least 12 months; failure of medically supervised conservative treatment	61.8 (med)	10%	17
Byrne 2007[32]	#3856	513	Confirmed mild-to-moderate neuropathic faecal incontinence.	61.9	11%	14
Wexner 2010[27]	#2918	67	Chronic FI > 6 months or >12 months after vaginal childbirth; >2 incontinent episodes on average per week, not just staining; failure or not candidates for more conservative treatments; ≥18 yrs	60.5	8%	12
Boyle 2011[36]	#2320	50	Faecal incontinence unresponsive to conservative management; symptoms that were refractory to maximal conservative treatment (including biofeedback), and had undergone a detailed clinical history and investigation to exclude any obvious organic cause for their incontinence.	55	6%	12
Tijandra 2008[33]	#11675	53	Involuntary passage of solid or liquid stool ≥1/wk; refractory to medical therapy and pelvic floor exercises; age 35 to 86 years	63.4	8%	10
Paquette 2014[28]	#6392	111	Patients with >2 episodes of FI per week;failure of conservative measures; for chronic implantation >50% improvement of symptoms during stage 1 test stimulation	67 (med)	NR	9
Sze 2009[31]	#3161	59	Women who had faecal incontinence at least once a week regardless of the amount of loss, for at least six months. Those who were incontinent of liquid stool, were referred for evaluation and colonoscopy, and were included in the study if they had benign findings.	58.6	0	9
Uludag 2011[35]	#2304	50	Patients, in whom conservative treatment had failed, underwent permanent SNM when a decrease of at least 50% in the number of incontinence episodes was seen during peripheral nerve evaluation (PNE).	54.3 (med)	10%	8

Study name	Endnote	N	Inclusion criteria	Mean/ Median age (yrs)	% of males	RoB No. Low*
**MEN**						
Lacima 2010[30]	#2779	79	Patients with presence of involuntary faecal leakage to formed stool with the following question: Do you ever have involuntary leakage of solid or liquid stools?	60.6	85%	11

*) Number of low risk items in the Risk of Bias assessment (out of 20 items in total – See Table 5)

Table S13: Treatment characteristics in studies for patients with FI

Study name	Endnote	Treatment	Further details of intervention	Duration of treatment
**WOMEN**				
**WOMEN – Biofeedback**				
Byrne 2007	#3856	Biofeedback	They were then scheduled to have six 20-minute biofeedback exercise treatment sessions by the same nurse at monthly intervals.	6 months
Damon 2014	#1373	Biofeedback: Perineal retraining, including biofeedback, in addition to standard treatments.	These 20 sessions included 5 initial sessions dealing with education, optimization of rectal evacuation (anal relaxation, body position, and gentle squeezing pressure), and rectal sensitivity (rectal isovolumic distension testing in order to decrease the first sensation threshold into normal ranges). The retraining of anal voluntary squeeze and abdominal and pelvic coordination completed them. The following 15 sessions aimed at acquiring competence at anal voluntary squeeze and at abdominal and pelvic coordination (manual techniques, BFB with anal device, and abdominal breathing). The squeezing exercises combined long-duration contractions and short-duration contractions using a biofeedback device. Each series of contractions was separated from the other by a resting period of which the duration was twice as long as the squeezing period. These sessions were completed by daily home-based anal exercises combining short and long contractions.	20 sessions of 30 min with the physiotherapist, all performed within a 4-month period at an initial rate of 2–3 sessions per week for the first 4 weeks, and 1–2 sessions per week thereafter.
**WOMEN – Sacral nerve/Neuromodulation**				
Boyle 2011	#2320	Sacral nerve stimulation	Patients underwent unilateral PNE with the percutaneous insertion of a stimulating electrode (3065USC; Medtronic, Minneapolis, MN) into the sacral foramen under general or local anaesthesia. Those who experienced significantly reduced FI with temporary SNS subsequently underwent permanent stimulation with a unilateral tined lead and pulse generator.	NR
Paquette 2014	#6392	Sacral nerve stimulation	InterStim system (Medtronic: Minneapolis, MN)	12 months
Tijandra 2008	#11675	Sacral nerve stimulation	Medtronic Interstim™ model 3625, Minneapolis, MN; a; procedure of implantation	12 months
Wexner 2010	#2918	Sacral nerve stimulation	Interstim system (Medtronic: Minneapolis, MN); procedure and programming was described	28 months
Uludag 2011	#2304	Sacral	No details provided, only referred to reference 14.	NR

Study name	Endnote	Treatment	Further details of intervention	Duration of treatment
		neuromodulation		
**WOMEN – Periferal Stimulation**				
Leroi 2012	#9773	TENS	Self-adhesive surface stimulation electrodes connected to a TENS Eco Program P3 stimulator (Schwa Medico, Ehringshausen, Germany); 20-min stimulation sessions twice daily for 3 months	3 months
**WOMEN – Drug treatments**				
Sze 2009	#3161	Methylcellulose (Citrucel) and loperamide (Imodium)	One heaping tablespoon of methylcellulose twice a day, which was increased to two heaping tablespoons twice a day as needed. If the incontinence persisted after taking maximum dose of methylcellulose for two weeks, an antidiarrhoeal agent, loperamide, one capsule twice a day was added, which was increased to two capsules three times a day as needed. If faecal urgency and incontinence resolved, the therapy was continued for a three-month treatment period. If faecal urgency and/or incontinence recurred during the three-month treatment period, methylcellulose was increased and loperamide was added or increased as needed until faecal urgency and/or incontinence resolved or the maximum dose of fibre and antidiarrhoeal agent was reached.	3 months
**WOMEN – Standard conservative treatment**				
Damon 2014	#1373	Standard conservative treatment	Not described	NR
**WOMEN – Bulking Agents**				
Graf 2011	#10132	Biomaterial injection	Dextranomer in stabilised hyaluronic acid (NASHA Dx)	NR
**MEN**				
**MEN – Biofeedback**				
Lacima 2010	#2779	Biofeedback	Training sessions consisting of strength training, sensory training, and coordination training. Each session took 45 min and the number of sessions each patient received was five. Additional sessions were given in selected cases. The first three were for every two weeks and subsequent sessions were for every month.	NR

Table S14: Results of interventions for patients with FI

Study name	Endnote	Intervention	N	Follow-up period	Type of outcome	Outcome description	With or without containment products	% of patients with outcome
**WOMEN – Biofeedback**								
Byrne 2007	#3856	Biofeedback	385	6m	Much improved (objective)	NR	NR	30%
Byrne 2007	#3856	Biofeedback	385	6m	Improved (Objective)	NR	NR	38%
Byrne 2007	#3856	Biofeedback	385	6m	Improved (Subjective)	NR	NR	78%
Damon 2014	#1373	Biofeedback: Perineal retraining, including biofeedback, in addition to standard treatments	67	4m	Success	Self-evaluated improvement was the primary outcome measure. A score ≥3 (in an improvement scale from −5 to +5) defined success	NR	57%
**WOMEN – Sacral nerve/Neuromodulation**								
Boyle 2011	#2320	Sacral nerve stimulation	50	3m	Success	Decrease of at least 50% in the number of incontinence episodes seen during peripheral nerve evaluation (PNE)	NR	54.0%
Boyle 2011	#2320	Sacral nerve stimulation	50	17m	Success	Decrease of at least 50% in the number of incontinence episodes seen during peripheral nerve evaluation (PNE)	NR	54.0%
Boyle 2011	#2320	Sacral nerve stimulation	50	17m	Cure	No episodes of Faecal Incontinence during the 2-week monitoring period	NR	26.0%
Paquette 2014	#6392	Sacral nerve stimulation	111	12m	Subjective Wexner score	75-100% improvement	NR	70.0%
Paquette 2014	#6392	Sacral nerve stimulation	111	12 m	Subjective Wexner score	>50 improvement	NR	93.0%

Study name	Endnote	Intervention	N	Follow-up period	Type of outcome	Outcome description	With or without containment products	% of patients with outcome
Tijandra 2008	#11675	Sacral nerve stimulation	53	3m	Improvement - 100%	100% improvement in incontinence episodes per week	NR	38.6%
Tijandra 2008	#11675	Sacral nerve stimulation	53	6m	Improvement - 100%	100% improvement in incontinence episodes per week	NR	31.7%
Tijandra 2008	#11675	Sacral nerve stimulation	53	12m	Improvement - 100%	100% improvement in incontinence episodes per week	NR	41.5%
Tijandra 2008	#11675	Sacral nerve stimulation	53	3m	Improvement - 75-99%	reductions of 75-99%, from baseline in incontinence episodes/week	NR	11.4%
Tijandra 2008	#11675	Sacral nerve stimulation	53	6m	Improvement - 75-99%	reductions of 75-99%, from baseline in incontinence episodes/week	NR	26.8%
Tijandra 2008	#11675	Sacral nerve stimulation	53	12m	Improvement - 75-99%	reductions of 75-99%, from baseline in incontinence episodes/week	NR	24.4%
Tijandra 2008	#11675	Sacral nerve stimulation	53	3m	Improvement - 50-74%	reductions of 50-74%, from baseline in incontinence episodes/week	NR	22.7%
Tijandra 2008	#11675	Sacral nerve stimulation	53	6m	Improvement - 50-74%	reductions of 50-74%, from baseline in incontinence episodes/week	NR	17.1%
Tijandra 2008	#11675	Sacral nerve stimulation	53	12m	Improvement - 50-74%	reductions of 50-74%, from baseline in incontinence episodes/week	NR	4.9%
Tijandra 2008	#11675	Sacral nerve stimulation	53	3m	Improvement - <50%	reductions of <50%, from baseline in incontinence episodes/week	NR	27.3%
Tijandra 2008	#11675	Sacral nerve stimulation	53	6m	Improvement - <50%	reductions of <50%, from baseline in incontinence episodes/week	NR	24.4%
Tijandra 2008	#11675	Sacral nerve stimulation	53	12m	Improvement - <50%	reductions of <50%, from baseline in incontinence episodes/week	NR	29.3%
Tijandra 2008	#11675	Sacral nerve stimulation	53	3m	Improvement - 100%	100% improvement in days with incontinence per week	NR	51.1%

Study name	Endnote	Intervention	N	Follow-up period	Type of outcome	Outcome description	With or without containment products	% of patients with outcome
Tijandra 2008	#11675	Sacral nerve stimulation	53	6m	Improvement - 100%	100% improvement in days with incontinence per week	NR	40.9%
Tijandra 2008	#11675	Sacral nerve stimulation	53	12m	Improvement - 100%	100% improvement in days with incontinence per week	NR	38.1%
Tijandra 2008	#11675	Sacral nerve stimulation	53	3m	Improvement - 75-99%	reductions of 75-99%, from baseline in days with incontinence per week	NR	8.9%
Tijandra 2008	#11675	Sacral nerve stimulation	53	6m	Improvement - 75-99%	reductions of 75-99%, from baseline in days with incontinence per week	NR	15.9%
Tijandra 2008	#11675	Sacral nerve stimulation	53	12m	Improvement - 75-99%	reductions of 75-99%, from baseline in days with incontinence per week	NR	16.7%
Tijandra 2008	#11675	Sacral nerve stimulation	53	3m	Improvement - 50-74%	reductions of 50-74%, from baseline in days with incontinence per week	NR	8.9%
Tijandra 2008	#11675	Sacral nerve stimulation	53	6m	Improvement - 50-74%	reductions of 50-74%, from baseline in days with incontinence per week	NR	11.4%
Tijandra 2008	#11675	Sacral nerve stimulation	53	12m	Improvement - 50-74%	Reductions of 50-74%, from baseline in days with incontinence per week	NR	9.5%
Tijandra 2008	#11675	Sacral nerve stimulation	53	3m	Improvement - <50%	reductions of <50%, from baseline in days with incontinence per week	NR	31.1%
Tijandra 2008	#11675	Sacral nerve stimulation	53	6m	Improvement - <50%	reductions of <50%, from baseline in days with incontinence per week	NR	31.8%
Tijandra 2008	#11675	Sacral nerve stimulation	53	12m	Improvement - <50%	reductions of <50%, from baseline in days with incontinence per week	NR	35.7%
Tijandra 2008	#11675	Sacral nerve stimulation	53	12 m	Cure	Continence	NR	47.2%

Study name	Endnote	Intervention	N	Follow-up period	Type of outcome	Outcome description	With or without containment products	% of patients with outcome
Wexner 2010	#2918	Sacral nerve stimulation	113	3m	Cure	Total continence (FI episodes per week)	NR	38.9%
Wexner 2010	#2918	Sacral nerve stimulation	107	6m	Cure	Total continence (FI episodes per week)	NR	39.3%
Wexner 2010	#2918	Sacral nerve stimulation	106	12m	Cure	Total continence (FI episodes per week)	NR	40.6%
Wexner 2010	#2918	Sacral nerve stimulation	67	24m	Cure	Total continence (FI episodes per week)	NR	37.3%
Wexner 2010	#2918	Sacral nerve stimulation	113	3m	Improvement 75% to 100%	Improvement in FI episodes per week	NR	29.2%
Wexner 2010	#2918	Sacral nerve stimulation	107	6m	Improvement 75% to 100%	Improvement in FI episodes per week	NR	31.8%
Wexner 2010	#2918	Sacral nerve stimulation	106	12m	Improvement 75% to 100%	Improvement in FI episodes per week	NR	28.3%
Wexner 2010	#2918	Sacral nerve stimulation	67	24m	Improvement 75% to 100%	Improvement in FI episodes per week	NR	34.3%
Wexner 2010	#2918	Sacral nerve stimulation	113	3m	Improvement 50% to 75%	Improvement in FI episodes per week	NR	16.8%
Wexner 2010	#2918	Sacral nerve stimulation	107	6m	Improvement 50% to 75%	Improvement in FI episodes per week	NR	17.8%
Wexner 2010	#2918	Sacral nerve stimulation	106	12m	Improvement 50% to 75%	Improvement in FI episodes per week	NR	14.2%
Wexner 2010	#2918	Sacral nerve stimulation	67	24m	Improvement 50% to 75%	Improvement in FI episodes per week	NR	13.4%

Study name	Endnote	Intervention	N	Follow-up period	Type of outcome	Outcome description	With or without containment products	% of patients with outcome
Wexner 2010	#2918	Sacral nerve stimulation	113	3m	Improvement 0% to 50%	Improvement in FI episodes per week	NR	8.9%
Wexner 2010	#2918	Sacral nerve stimulation	107	6m	Improvement 0% to 50%	Improvement in FI episodes per week	NR	7.5%
Wexner 2010	#2918	Sacral nerve stimulation	106	12m	Improvement 0% to 50%	Improvement in FI episodes per week	NR	9.4%
Wexner 2010	#2918	Sacral nerve stimulation	67	24m	Improvement 0% to 50%	Improvement in FI episodes per week	NR	7.5%
Wexner 2010	#2918	Sacral nerve stimulation	113	3m	Improvement >50%	Weekly incontinent episodes	NR	85.0%
Wexner 2010	#2918	Sacral nerve stimulation	107	6m	Improvement >50%	Weekly incontinent episodes	NR	89.0%
Wexner 2010	#2918	Sacral nerve stimulation	106	12m	Improvement >50%	Weekly incontinent episodes	NR	83.0%
Wexner 2010	#2918	Sacral nerve stimulation	67	24m	Improvement >50%	Weekly incontinent episodes	NR	85.0%
Wexner 2010	#2918	Sacral nerve stimulation	113	3m	Improvement >50%	Weekly urgent incontinent episodes	NR	80.0%
Wexner 2010	#2918	Sacral nerve stimulation	107	6m	Improvement >50%	Weekly urgent incontinent episodes	NR	83.0%
Wexner 2010	#2918	Sacral nerve stimulation	106	12m	Improvement >50%	Weekly urgent incontinent episodes	NR	80.0%
Wexner 2010	#2918	Sacral nerve stimulation	67	24m	Improvement >50%	Weekly urgent incontinent episodes	NR	79.0%
Wexner 2010	#1791	Sacral nerve stimulation	72	3m	Success rate	At least a 50% improvement from baseline in weekly incontinent episodes in bowel diary data.	NR	90.0%

Study name	Endnote	Intervention	N	Follow-up period	Type of outcome	Outcome description	With or without containment products	% of patients with outcome
Wexner 2010	#1791	Sacral nerve stimulation	72	3m	Cure	Achievement of total continence	NR	43.1%
Wexner 2010	#1791	Sacral nerve stimulation	71	6m	Success rate	At least a 50% improvement from baseline in weekly incontinent episodes in bowel diary data.	NR	96.0%
Wexner 2010	#1791	Sacral nerve stimulation	71	6m	Cure	Achievement of total continence	NR	45.1%
Wexner 2010	#1791	Sacral nerve stimulation	73	1y	Success rate	At least a 50% improvement from baseline in weekly incontinent episodes in bowel diary data.	NR	89.0%
Wexner 2010	#1791	Sacral nerve stimulation	73	1y	Cure	Achievement of total continence	NR	45.1%
Wexner 2010	#1791	Sacral nerve stimulation	66	2y	Success rate	At least a 50% improvement from baseline in weekly incontinent episodes in bowel diary data.	NR	91.0%
Wexner 2010	#1791	Sacral nerve stimulation	66	2y	Cure	Achievement of total continence	NR	39.4%
Wexner 2010	#1791	Sacral nerve stimulation	72	3y	Success rate	At least a 50% improvement from baseline in weekly incontinent episodes in bowel diary data.	NR	89.0%
Wexner 2010	#1791	Sacral nerve stimulation	72	3y	Cure	Achievement of total continence	NR	41.7%
Wexner 2010	#1791	Sacral nerve stimulation	73	4y	Success rate	At least a 50% improvement from baseline in weekly incontinent episodes in bowel diary data.	NR	88.0%
Wexner 2010	#1791	Sacral nerve stimulation	73	4y	Cure	Achievement of total continence	NR	35.6%
Wexner 2010	#1791	Sacral nerve stimulation	72	5y	Success rate	At least a 50% improvement from baseline in weekly incontinent episodes in bowel diary data.	NR	89.0%
Wexner 2010	#1791	Sacral nerve stimulation	72	5y	Cure	Achievement of total continence	NR	36.1%

Study name	Endnote	Intervention	N	Follow-up period	Type of outcome	Outcome description	With or without containment products	% of patients with outcome
Uludag 2011	#2304	Sacral neuromodulation	50	7y	Improvement	Decrease of at least 50% in the number of incontinence episodes seen during peripheral nerve evaluation (PNE)	NR	84.0%
**WOMEN – Periferal Stimulation**								
Leroi 2012	#9773	TENS	66	3 mo	Subjective cure	Cleveland Clinic Continence Scoring System score 0	NR	6%
Leroi 2012	#9773	TENS	66	3 mo	Subjective improvement	> 30 % decrease in CCS score	NR	47%
**WOMEN – Drug treatments**								
Sze 2009	#3161	Methylcellulose (Citrucel) and loperamide (Imodium)	59	3m	Cure	Patient stated that her incontinence was cured, had zero Pescatori incontinence point, resolution of faecal urgency, did not use a perineal pad for protection, and her incontinence did not have any effect on her emotional, social, occupational, and physical functions during the treatment or observation period.	Without	46%
**WOMEN – Standard conservative treatment**								
Damon 2014	#1373	Standard conservative treatment (control)	75	4m	Success	Self-evaluated improvement was the primary outcome measure. A score ≥3 (in an improvement scale from −5 to +5) defined success	NR	37%
**WOMEN – Bulking Agents**								
Graf 2011	#10132	Biomaterial injection	132	6m	Improvement (response)	A reduction in number of episodes by 50% or more compared with baseline	NR	52.7%
Graf 2011	#10132	Biomaterial injection	125	12m	Improvement (response)	A reduction in number of episodes by 25% or more compared with baseline	NR	69.1%

Study name	Endnote	Intervention	N	Follow-up period	Type of outcome	Outcome description	With or without containment products	% of patients with outcome
**MEN**								
**MEN – Biofeedback**								
Lacima 2010	#2779	Biofeedback	53	6m	Cure	Continence fully recovered	NR	40.80%
Lacima 2010	#2779	Biofeedback	53	6m	Improvement	Reduction in the number of episodes of incontinence greater than 75%	NR	47.90%
Lacima 2010	#2779	Biofeedback	53	3y	Cure	Continence fully recovered	NR	35.80%
Lacima 2010	#2779	Biofeedback	31	3y	Improvement	Reduction in the number of episodes of incontinence greater than 75%	NR	47.20%
Lacima 2010	#2779	Biofeedback	31	5y	Cure	Continence fully recovered	NR	29%
Lacima 2010	#2779	Biofeedback	31	5y	Improvement	Reduction in the number of episodes of incontinence greater than 75%	NR	61.30%

Table S15: Study characteristics for patients with neurological problems or diseases

Study name	Endnote	N	Inclusion criteria	Mean/ Median age (yrs)	% of males	RoB No. Low*
**WOMEN**						
Leroi 2012[16]	#9773	66	Patients with FI>3 months; failure of conservative treatments; not a candidate for conservative treatments; ≥ 18 years of age	60 (med)	7%	18
Sussman 2013[39]	#11682	166	18 to 80 years of age with NDO (due to MS or SCI) and 14 UI episodes/week who were not adequately managed by anticholinergics (defined as an inadequate response or intolerable side effects after at least 1 month of an optimized dose).	45	42.1%	15
Thomas 2014[38]	#15557	330	"Stroke services: specialist acute and rehabilitation services (either separate or combined units). Patients: >= 18 years; a diagnosis of stroke based on the World Health Organisation (WHO) criteria; UI as defined by the International Continence Society as 'involuntary loss of urine' and classified as stress UI (any response other than 'never' to the Leicester Urinary Symptom questionnaire (LUSQ) question 'Do you ever leak when you do any of the following?'), urge UI (the response 'most of the time', 'sometimes' or 'occasionally' to the LUSQ question 'When you get the urge to pass urine, does any leak before you get to the toilet?'), mixed UI (both stress and urge UI) or 'functional' UI, defined as mobility or balance restrictions stopping patients reaching the toilet on time or presence of indwelling catheter in the acute phase of the stroke; Conscious (defined as either 'alert' or 'drowsy' on the 'Clinical Status on Admission' item of the European Stroke Database); Medically stable as judged by the clinical team"	79 (Med)	46%	9
Faaborg 2009[37]	#13213	211	Neurological bowel disease; failed conservative treatment	49 (Med)	45%	9

*) Number of low risk items in the Risk of Bias assessment (out of 20 items in total – See Table 5)

Table S16: Treatment characteristics in studies for patients with neurological problems or diseases

Study name	Endnote	Treatment	Further details of intervention	Duration of treatment
**WOMEN**				
**WOMEN – Botulinum Toxin**				
Sussman 2013	#11682	Onabotulinumtoxin A 200U	Administered via cystoscopy, avoiding the trigone	12 wks
Sussman 2013	#11682	Onabotulinumtoxin A 300U	Administered via cystoscopy, avoiding the trigone	12 wks
**WOMEN – Periferal Stimulation**				
Leroi 2012	#9773	TENS	Self-adhesive surface stimulation electrodes connected to a TENS Eco Program P3 stimulator (Schwa Medico, Ehringshausen, Germany); 20-min stimulation sessions twice daily for 3 months	3 months
**WOMEN – Transanal irrigation**				
Faaborg 2009	#13213	Transanal irrigation	Instruction and then telephone contact; no other details	19 months
**WOMEN – Voiding programme**				
Thomas 2014	#15557	Systematic voiding programme (SVP)	detailed description on p. 3: Patients cognitively able received bladder training and those with cognitive impairment received prompted voiding	12 wks
Thomas 2014	#15557	SVP and supported implementation	detailed description pn p. 4: SVP introduced using an implementation strategy, facilitation, to assist the process of embedding into practice	12 ws
**WOMEN – Other**				
Thomas 2014	#15557	Usual continence care	checking for urinary tract infection; checking for overflow incontinence; containment using a variety of products (for example absorbent pads) with regular changes; and some form of toileting schedule	12 wks

Table S17: Results of interventions for patients with neurological problems or diseases

Study name	Endnote	Intervention	N	Follow-up period	Type of outcome	Outcome description	With or without containment products	% of patients with outcome
**WOMEN – Botulinum Toxin**								
Sussman 2013	#11682	OnabotulinumtoxinA 200U	85	12 wks	Improvement I-QoL	Proportion of patients who achieved a >=11point increase from baseline on I-QoL scores, which is clinically meaningful.	Without	69.4%
Sussman 2013	#11682	OnabotulinumtoxinA 300U	81	12 wks	Improvement I-QoL	Same as above	Without	65.4%
Sussman 2013	#11682	OnabotulinumtoxinA 200U	85	12 wks	Improvement OAB-PSTQ Satisfaction	Treatment satisfaction: proportion of patients who reported feeling very or somewhat satisfied.	Without	70.6%
Sussman 2013	#11682	OnabotulinumtoxinA 300U	77	12 wks	Improvement OAB-PSTQ Satisfaction	Same as above	Without	70.1%
Sussman 2013	#11682	OnabotulinumtoxinA 200U	84	12 wks	Improvement OAB-PSTQ Achievement of treatment goals	Achievement of treatment goals: proportion of patients who rated their progress as ‘‘significant’’ or ‘‘complete’’	Without	57.1%
Sussman 2013	#11682	OnabotulinumtoxinA 300U	78	12 wks	Improvement OAB-PSTQ Achievement of treatment goals	Same as above	Without	57.7%
Sussman 2013	#11682	OnabotulinumtoxinA 200U	84	12 wks	Improvement OAB-PSTQ Met treatment expectations	Meeting treatment expectations: proportion of patients rating their progress as ‘‘significantly met’’ or ‘‘exceeded’’ expectations.	Without	57.1%
Sussman 2013	#11682	OnabotulinumtoxinA 300U	78	12 wks	Improvement OAB-PSTQ Met treatment	Same as above	Without	57.7%

Study name	Endnote	Intervention	N	Follow-up period	Type of outcome	Outcome description	With or without containment products	% of patients with outcome
					expectations			
Sussman 2013	#11682	OnabotulinumtoxinA 200U	85	12 wks	Improvement in HRQol	Qol on PGA	NR	45%
Sussman 2013	#11682	OnabotulinumtoxinA 300U	78	12 wks	Improvement in HRQol	Qol on PGA	NR	50%
**WOMEN – Periferal Stimulation**								
Leroi 2012	#9773	TENS	66	3 mo	Subjective cure	Cleveland Clinic Continence Scoring System score 0	NR	6%
Leroi 2012	#9773	TENS	66	3 mo	Subjective improvement	> 30 % decrease in CCS score	NR	47%
**WOMEN – Transanal irrigation**								
Faaborg 2009	#13213	Transanal irrigation	211	19m	Cure	No longer need transanal irrigation	NR	9%
**WOMEN – Voiding programme**								
Thomas 2014	#15557	systematic voiding programme (SVP)	132	12 wks	Cure	Absence of incontinence was defined as the response 'never' to question three, 'How often do you leak urine?	Without	41%
Thomas 2014	#15557	SVP and supported implementation	100	12 wks	Cure	Same as above	Without	31%
**WOMEN – Other**								
Thomas 2014	#15557	usual continence care	98	12 wks	Cure	Same as above	Without	30%

Table S18: Study characteristics for elderly or cognitively impaired patients with incontinence

Study name	Endnote	N	Inclusion criteria	Mean age (yrs)	% of males	RoB No. Low*
Chapple 2007a[24]	#11920	266	Men and women; age ≥65 yrs; symptoms of OAB ≥ 6mo, recruited from various types of practices; capable of independent toileting and able to complete diary; completed at least 5 days of the 7-day diary during b-line period; OAB symptoms, including an average of >=1 urge UI/day and >=10 micrutritions/day.	72	22.6%	15
Dubeau 2014[25]	# 15558	562	Eligible men or women were 65 years old or older with self-reported UUI symptoms for >= 3 months, a mean of 2 to 15 UUI episodes, 8 or more micturitions per 24 hours on baseline 3-day bladder diary, and at least some moderate bladder related problem on the PPBC (Patient Perception of Bladder Condition) who were determined to be vulnerable (at risk of deteriorating health) by a score of 3 or more on the VES-13 at screening.	75	20%	16
Liao 2013[26]	#15559	61	Frail elderly (>65y) patients diagnosed with urodynamic IDO refractory to previous antimuscarinics for more than 3 months.	76	56%	14
Liao 2013[26]	#15559	63	Elderly (>65y) patients without frailty, diagnosed with urodynamic IDO refractory to previous antimuscarinics for more than 3 months.	76	70%	14
Wagg 2014[23]	#15549	305	Male or female subjects >=65 years old with OAB symptoms (subject-reported) for >=3 months prior to Screening visit according to ICS guidelines, mean urinary frequency of >=8 micturitions per 24 hours as verified by the micturition diary prior to Randomization/ Baseline visit, and mean number of urgency episodes per 24 hours as verified by the Screening micturition diary prior to Baseline (urgency episodes were defined as those with Urinary Sensation Scale [USS] rating >=3).	72.3	46%	10

*) Number of low risk items in the Risk of Bias assessment (out of 20 items in total – See Table 5)

Table S19: Treatment characteristics in studies for elderly or cognitively impaired patients with incontinence

Study name	Endnote	Treatment	Further details of intervention	Duration of treatment
**Elderly – Antimuscarinics**				
Wagg 2014	#15549	Fesoterodine SR 4 mg or 8 mg tablets	Patients started on a dose of fesoterodine SR 4 mg or equivalent placebo QD. After 4 weeks patients were permitted a one-time dose increase to 8 mg, if needed, in order to further optimize their treatment. If they remained on the 4 mg dose after 4 weeks of treatment, they were permitted to increase their dose to 8 mg after 8 weeks of treatment if necessary.	24 weeks
Dubeau 2014	# 15558	Fesoterodine	Fesoterodine 4mg dose. At week 4 visit could be increased to 8mg depending on efficacy and tolerability.	12 wks
Chapple 2007a	#11920	Darifenacin	7.5 up-titrated to 15 mg	12 wks
**Elderly – Botulinum toxin**				
Liao 2013	#15559	Botulinum toxin	OnabotulinumtoxinA 100U	Single procedure

Table S20: Results of interventions for elderly or cognitively impaired patients with incontinence

Study name	Endnote	Intervention	N	Follow -up period	Type of outcome	Outcome description	With or without containment products	% of patients with outcome
**Antimuscarinics**								
Wagg 2014	#15549	Fesoterodine Flexible Dose	299	24w	Improvement	Patient Treatment Benefit Scale (PTBS) – % of subjects with improvement (responders) - Proportion of subjects who reported their treatment has improved (1= greatly improved, 2= improved).	NR	78.30%
Dubeau 2014	# 15558	Fesoterodine	256	12 wks	Cure	Diary dry rate- proportion of subjects with 1 or more UUI episodes on baseline diary and no UUI episodes on posttreatment diary	Without	50.8%
Chapple 2007a	#11920	Darifenacin	266	12 wks	Cure	3 day dry rate	Without	48.5%
Chapple 2007a	#11920	Darifenacin	266	12 wks	Cure	7 day dry rate	Without	32.7%
Chapple 2007a	#11920	Darifenacin	266	12 wks	Improvement ≥90%	Response - improvement in mean number of UUIE per week	Without	46%
Chapple 2007a	#11920	Darifenacin	266	12 wks	Improvement ≥70%	Response - improvement in mean number of UUIE per week	Without	58%
Chapple 2007a	#11920	Darifenacin	266	12 wks	Improvement ≥50%	Response - improvement in mean number of UUIE per week	Without	70%
Chapple 2007a	#11920	Darifenacin	266	12 wks	Improvement ≥30%	Response - improvement in mean number of UUIE per week	Without	76%
**Botulinum toxin**								
Liao 2013	#15559	OnabotulinumtoxinA	61	3m	Success	Success rate- A Patient Perception of Bladder Condition decrease of 2 points was considered successful	Without	83.4%
Liao 2013	#15559	OnabotulinumtoxinA	61	6m	Success	Same as above	Without	44.9%
Liao 2013	#15559	OnabotulinumtoxinA	61	12m	Success	Same as above	Without	6.82%

Liao 2013	#15559	OnabotulinumtoxinA	63	3m	Success	Same as above	Without	91.2%
Liao 2013	#15559	OnabotulinumtoxinA	63	6m	Success	Same as above	Without	52.1%
Liao 2013	#15559	OnabotulinumtoxinA	63	12m	Success	Same as above	Without	22.3%
